# Planar aromatic anchors control the electrical conductance of gold|molecule|graphene junctions†

**DOI:** 10.1039/d2na00873d

**Published:** 2023-03-27

**Authors:** Luke J. O'Driscoll, Michael Jay, Benjamin J. Robinson, Hatef Sadeghi, Xintai Wang, Becky Penhale-Jones, Martin R. Bryce, Colin J. Lambert

**Affiliations:** a Dept. of Chemistry, Durham University Durham DH1 3LE UK m.r.bryce@durham.ac.uk; b Dept. of Physics, Lancaster University Lancaster LA1 4YB UK c.lambert@lancaster.ac.uk; c Dept. of Engineering, Warwick University Coventry CV4 7AL UK; d School of Information Science and Technology, Dalian Maritime University Dalian China

## Abstract

The synthesis of a family of alkanethiol molecules with planar aromatic head groups, designed to anchor molecules effectively to graphene electrodes, is reported. Characterisation of self-assembled monolayers of these molecules on a gold surface *via* conductive atomic force microscopy shows that when an aromatic head group is present, the conductance *G*_graphene_ obtained using a graphene coated probe is higher than the conductance *G*_Pt_ obtained using a platinum (Pt) probe. For Pt probe and graphene probe junctions, the tunnelling decay constant of benzyl ether derivatives with an alkanethiol molecular backbone is determined as *β* = 5.6 nm^−1^ and 3.5 nm^−1^, respectively. The conductance ratio *G*_graphene_/*G*_Pt_ increases as the number of rings present in the aromatic head unit, *n*, increases. However, as the number of rings increases, the conductance path length increases because the planar head groups lie at an angle to the plane of the electrodes. This means that overall conductance decreases as *n* increases. Density functional theory-based charge transport calculations support these experimental findings. This study confirms that planar aromatic head groups can function as effective anchoring units for graphene electrodes in large area molecular junctions. However, the results also indicate that the size and geometry of these head groups must be considered in order to produce effective molecular designs.

## Abstract 2

Alkanethiol molecules with planar aromatic head groups were synthesised and assembled on gold surfaces. The conductance properties of the resulting monolayers were measured using conductive atomic force microscopy with Pt and graphene coated tips.

## Graphical abstract

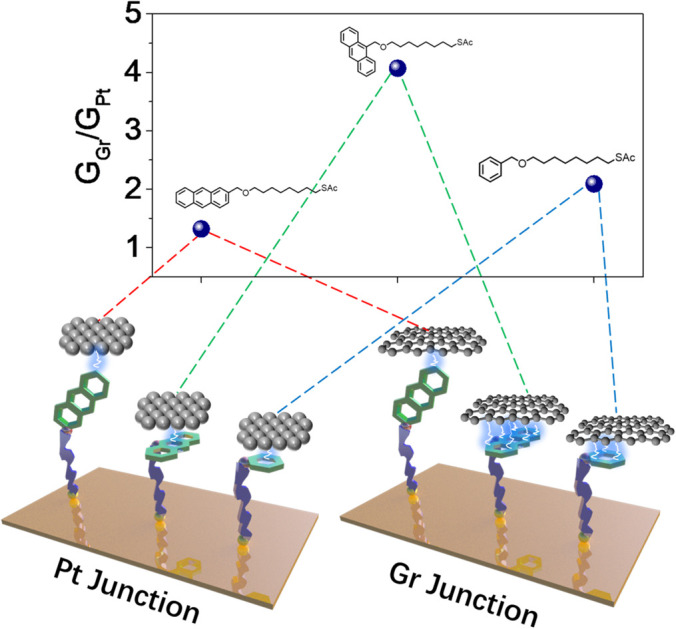

## Introduction

Molecular junctions, in which one or more molecules bridge the gap between a source and drain electrode, have been intensely studied during the past two decades.^1–17^ A variety of methods for engineering the transport properties of molecules have been developed, based on manipulating conformation,^18–22^ anchor groups,^8,23–27^ electrodes,^28–31^ quantum interference,^32–42^ and heteroatom substitution,^43–48^ both in single-molecule junctions and large-area junctions comprising self-assembled monolayers (SAMs) of organic molecules.^29,49–51^ Typically in single-molecule experiments using mechanically controllable break junction (MCBJ)^1^ or scanning tunnelling microscope (STM)^2^ contacting methodologies, gold is the electrode material of choice. However, there is a growing interest in the study of molecular junctions formed using graphene electrodes. For this purpose, planar aromatic moieties are desirable terminal groups for anchoring molecules to graphene electrodes owing to the strong π–π interactions between their planar π systems and graphitic surfaces.^25,28,30,52^ Methods used to determine the conductance of larger area junctions, such as conductive atomic force microscopy (cAFM), are well suited to investigations using different electrode materials.^53,54^

A major goal of molecular-scale electronics is the control of transport properties by systematically varying structural features of the molecule.^55^ Studies of polycyclic aromatic hydrocarbons on graphene surfaces have shown an increase in the binding energy^52^ as the number of fused rings increases. Additionally, in molecular junctions where a polycyclic aromatic hydrocarbon lies parallel to both electrodes, the “cross-plane conductance”^56^ (*i.e.* the conductance perpendicular to the plane of the electrodes and substrate) increases with the number of fused rings. This present study investigates whether these trends persist when planar aromatics are used as the top contact groups in Au|SAM|Pt and Au|SAM|graphene junctions. Firstly, the synthesis of a family of alkanethiol molecules with planar aromatic head groups of differing sizes is presented. The preparation and characterisation of SAMs of these molecules on Au substrates is reported, followed by cAFM studies of the SAMs in which the effect on conductance of using either a Pt tip or graphene-coated Pt tip was investigated. The experimental results are then compared with charge transport calculations, based on density functional theory (DFT) and molecular dynamics (MD) simulations.

## Results and discussion

### Molecular design and synthesis

**Fig. 1:**
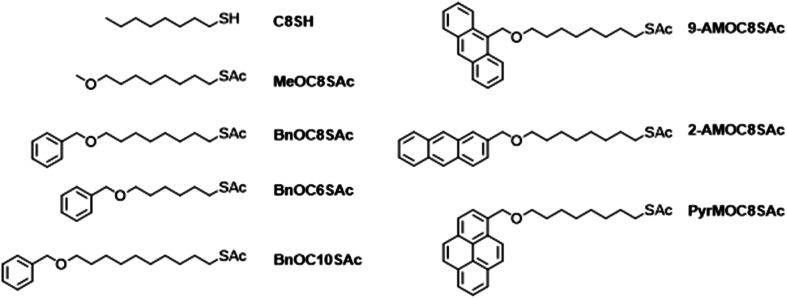
Structures of the molecules investigated in this study. The acetyl protecting group cleaves *in situ* during assembly on Au surfaces.

The investigated molecules are shown in Fig. 1. With the exception of commercial octanethiol (C8SH), each molecule is based on an *α*,*ω*-disubstituted linear alkyl chain, bearing an ether functionality at one end and an acetyl-protected thiol at the other. The following naming convention is used: the nature of the ether-linked head group (if present), is stated (using standard chemical nomenclature where possible; AM and PyrM refer to anthracenemethylene and pyrenemethylene groups, respectively), followed by the length of the alkyl chain as number of carbons (C*X*) then the terminal functionality (SAc for protected species, S for assembled molecules, presumed to be thiolates). For example, BnOC8SAc is an octyl chain substituted at each terminus; at one with a benzyl ether and at the other with a thioacetate functionality.

The (protected) thiol is designed to assemble on Au surfaces through S–Au bonds (after *in situ* loss of the acetyl protecting group), forming a SAM in which the alkyl or aryl head group is available to interact with an AFM tip. Alkyl chains are known to be less conductive than conjugated backbones and are uncommon in molecules designed for modern single-molecule conductance studies,^7^ although they are still useful benchmark compounds^57^ and were widely used in earlier studies.^3,58–60^ However, low molecular conductance is less problematic when studying large area molecular junctions as many molecules are measured in parallel. Alkyl backbones are synthetically convenient and were anticipated to result in dense, reasonably ordered SAMs as observed for simple alkanethiols and structurally similar species.^29,61^ Furthermore, it was anticipated that the increased flexibility of alkyl chains compared to widely-used, rigid, conjugated backbones such as oligo(phenylene ethynylene)s^16^ would afford more conformational freedom to the planar head groups, potentially permitting more efficient contact between the SAMs and the top electrode.

The studied compounds (Fig. 1) comprise two homologous series. In the first, the alkyl chain has a constant length (C8, *i.e.*, 1,8-disubstituted octanes) and the nature of the head group varies through the series: none, MeO, BnO, 9-AMO, 2-AMO or PyrMO. In the second series the alkyl chain length is varied (C6, C8, C10), while the head group remains the same (BnO, *i.e.*, benzyl ether). The different head groups were selected to investigate the effect of the presence, size and geometry of an aromatic anchor group on molecular conductance. The species bearing aryl ethers were prepared by statistical desymmetrisation of *α*,*ω*-alkanediols^62^ by reaction with the appropriate benzylic bromide,^63^ activation of the remaining alcohol as a mesylate and finally a nucleophilic substitution using potassium thioacetate. MeOC8SAc was prepared instead by desymmetrisation of 1,8-dibromooctane. Full details of the synthesis and characterisation of these molecules and their precursors are given in the Section S1 of the ESI.†

### SAM preparation and characterisation

The molecules were deposited onto a template stripped Au substrate (Au^TS^) by self-assembly as described in Section S2.1 of the ESI.† SAM characterisation is also described in Section S2.1 of the ESI,† together with representative images. In brief, the deposition of the thin film was monitored by a quartz crystal microbalance (QCM) to confirm SAM formation by determining the density of molecules adsorbed on the Au^TS^ surface. Atomic force microscopy (AFM) was used to determine the thickness of the molecular film *via* a nano-scratching method.^64,65^ This provided an indicator of the binding geometry of the molecule on the surface, as detailed in Table S1 in the ESI.† All the measured molecules were determined to have a tilt angle of 35°–55° with respect to the normal to the Au substrate. This is consistent with previous reports; the typical value for alkanethiols is 30°–35°,^61^ and the coexistence of regions with a tilt angle of around 50° has also been reported.^66^ SAM quality was further investigated by AFM topography. The roughness of the measured samples was in the range of 1.2 to 2.1 Å, which is comparable with the roughness of a clean Au^TS^ substrate (*ca.* 1.5 Å), indicating that the contour of the molecular layer followed the underlying gold surface.

### Electrical measurements

**Fig. 2:**
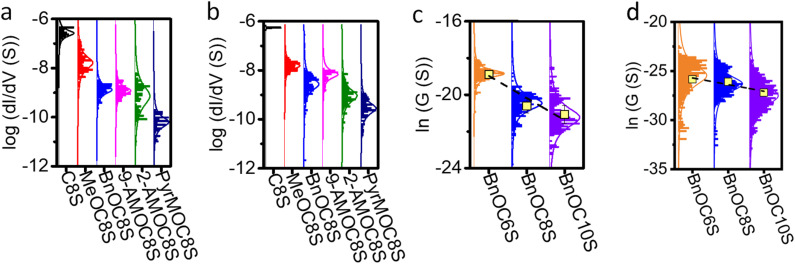
Experimental results for the electrical conductances of different SAMs. Conductance distribution of SAMs obtained using (a) a Pt coated probe and (b) a graphene coated probe. The distribution of conductances *versus* the number of alkyl units, obtained using (c) a Pt coated probe and (d) a graphene coated probe for molecules with a benzyl head group (average conductance is indicated by the yellow squares).

The electron transport properties of the SAMs were characterised using cAFM (see Section S2.2 of the ESI†). The Au^TS^ and an AFM probe coated with a conductive layer were used as source and drain respectively. d*I*/d*V* curves were obtained *via* mathematic differentiation of *IV* curves collected at a constant normal force of 2 nN shown to provide good electrical contact but negligible compression of the molecular film.^67^ Histograms shown in Fig. 2 represent d*I*/d*V* at near 0 bias (±10 mV), a region which has been shown to exhibit the strongest dependence on the quantum transport properties of the molecular junction.^68^ Previous reports have shown that electrical transport in SAMs is strongly dependent on the mechanically induced molecular geometry of the probe-sample junction^69^ arising from either a change in the number of molecules in the junction or a change in the tilt angle of the molecules relative to the substrate.^70–72^ The precise nature of possible mechanical deformation of the SAMs reported here is beyond the scope of this study. However, we have ensured consistent compression of the SAMs by performing all measurements at the same low applied force, as determined by equipment integrated thermal calibration of the AFM probe and force curve calibrations of the junction.

Both Pt and graphene^54^ were used as the conductive coating layer for the AFM probe. To estimate the number of molecules present in each junction (Table S2†), and hence the average conductance per molecule in a given SAM, the contact area between the Au^TS^ and probe was estimated by the JRK^72–74^ model (as detailed in Section S2.2 of the ESI†).

The resulting histograms of molecular conductance are shown in Fig. 2a and b. The statistically most probable conductance of each species is listed in Table 1. Fig. 2c shows the conductance decay of SAMs with benzyl head groups as a function of the number of alkyl units in the molecular backbone in Au|SAM|Pt junctions. This follows the characteristic relationship for conductance decay in molecular junctions: *G* ∝ *e*^−*βL*^ where *G* is molecular conductance, *L* is molecular length obtained from density functional theory (DFT) calculations, and *β* is the tunnelling decay constant of the molecular backbone. For the Pt probe junctions, we measure *β* = 5.6 nm^−1^, slightly below the typical range for alkanethiols and alkanedithiols in metallic junctions (*ca.* 8–10 nm^−1^).^75,76^ In comparison, for the graphene probe junctions we measure *β* = 3.5 nm^−1^ (Fig. 2d) which is consistent with recent studies that have shown that metal|molecule|graphene junction architectures afford lower *β* values than metal|molecule|metal junction architectures for alkane derivatives including alkanedithiols.^76,77^

**Table 1:** The statistically most probable conductance values per molecule from SAMs measured using Pt and graphene coated AFM probes, derived from the histograms in Fig. 2a and b, and the ratio of the most probable conductance values measured using the two different probes

Molecule	log(*G*_Pt_(*S*))	log(*G*_graphene_(*S*))	*G*_graphene_/*G*_Pt_
C8S	−6.58	−6.31	1.86
MeOC8S	−7.81	−7.82	0.98
BnOC8S	−8.94	−8.62	2.09
9-AMOC8S	−8.89	−8.28	4.07
2-AMOC8S	−9.25	−9.13	1.32
PyrMOC8S	−10.29	−9.58	5.13

### Transport calculations

Charge transport simulations of molecular junctions were carried out using the SIESTA^78^ density functional theory (DFT) code, combined with the Green's function transport code Gollum^79^ (see Section S3 of ESI†). In the simulations, the thiol terminal group is bound to a Au bottom electrode and the other end of the molecule (usually an ether-linked head group) is in contact with a top Au electrode. Further details are provided in Section S3.1 of the ESI.† Where head groups are present, the angle *θ* between the plane of the aromatic head group and the plane of the top electrode must be considered. The following discussion will use the formalism that *θ* = 0° when these planes are parallel and *θ* = 90° when these planes are perpendicular.

Molecular dynamics simulations modelling a SAM of BnOC8S showed that in the absence of a top graphene electrode, the headgroups tend to be positioned in an upright manner, with *θ* close to 90° (Fig. S46†). This is unsurprising as this conformation would be expected to minimise steric clash between neighbouring molecules. However, in the presence of a graphene top contact, the distribution of angles is centred at *θ* ≈ 55° (Fig. S46†). This rearrangement implies a shorter conductance pathway than would be expected for the fully extended molecule, which is likely to result in increased conductance. A more detailed discussion can be found in Section S3.2 of the ESI.†

**Fig. 3:**
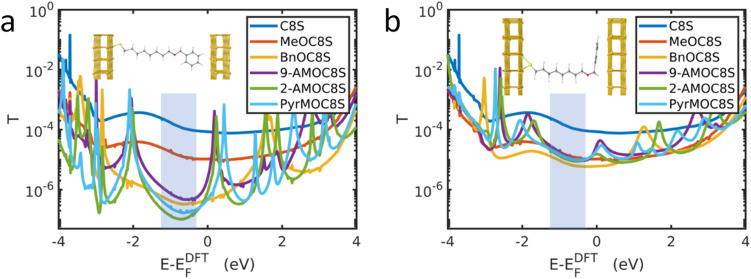
DFT-based transmission functions for Au|molecule|Au junctions. Where aromatic head groups are present (a) shows aromatic head groups approximately perpendicular to the top Au lead (*i.e.*, *θ* ≈ 90°, see inset) and (b) shows aromatic head groups approximately parallel to the top Au lead (*i.e.*, *θ* ≈ 0°, see inset). *E*^DFT^_F_ is the DFT-predicted Fermi energy. In practice, *E*^DFT^_F_ may not coincide with the experimental value *E*_F_, which typically lies near the middle of the HOMO–LUMO gap, indicated by the shaded regions.

The MD simulations do not represent the distribution of angles in the cAFM measurements described above, which will depend upon additional factors, such as surface roughness and molecular packing in the SAM. Due to the computational expense of carrying out DFT simulations on large distributions of angles, the two extremes of angle *θ* were studied for each molecule. Fig. 3a shows the simulated transmission functions *T*(*E*) for the molecular junctions in which the aromatic head groups are approximately perpendicular to the top electrode (*θ* ≈ 90°), while Fig. 3b shows the equivalent data for approximately parallel head groups (*θ* ≈ 0°). The electrical conductance of such junctions is approximately *G* = *G*_0_*T*(*E*_F_), where *E*_F_ is the Fermi energy and *G*_0_ ≈ 77 μS is the conductance quantum.

Past comparisons between experiment and theory suggest that *E*_F_ typically lies near the middle of the HOMO–LUMO gap,^44,80,81^ indicated by the shaded regions in Fig. 3. The trends in *T*(*E*_F_) observed in Fig. 3a are in qualitative agreement with the experimentally measured conductances. Starting from the relatively high *T*(*E*_F_) of the alkanethiol C8S, the addition of the methoxy group causes the transmission function in the shaded region to fall by around an order of magnitude. Fig. 3a shows that for fully extended, near-perpendicular conformations (*θ* ≈ 90°), *T*(*E*_F_) is lowered further by the addition of the aromatic head groups, with the head groups which increase the molecular length the most (PyrM, 2-AM) having the lowest transmission. Fig. 3b shows that when an aromatic head group is present, near-parallel configurations (*θ* ≈ 0°) are more conductive than those in which the head group is perpendicular to the Au lead. In the parallel configuration, the transmission of all ether-functionalised species is similar in the HOMO–LUMO gap. The implication is that in the experimental system it is unlikely that the aromatic head groups lie perpendicular to the top electrode surface. Rather, as supported by the MD simulations discussed above, the head groups present in the experimental junctions may display a distribution of angles between the limits of parallel and perpendicular configurations. Realistic transmission curves are therefore expected to lie between those shown in Fig. 3a and b. However, the intensive calculations required to afford such data are beyond the scope of the present study.

## Discussion

When considering the C8 alkyl chain series, two trends are apparent in the experimental data as the number *n* of fused rings in the aromatic head group changes. The first trend is that the conductance tends to decrease as the size of the aromatic head group increases. This result is seemingly in contrast to previous studies. It has been reported that the electrical conductance of planar aromatic molecules adsorbed on planar electrodes increases with their size, when current flows perpendicular to the plane of the molecules.^56^ Additionally, the binding energy between an aromatic head group and a planar electrode increases with the size of the head group.^52,82^ However, these studies are concerned with aromatic systems that are coplanar to the electrode surface. The MD simulations, the agreement between experimental data and trends observed in Fig. 3a, and SAM thickness analysis (Fig. S41 and S42†) indicate that in the present work the aromatic head groups are tilted with respect to the probe. A consequence of this tilting is that the current pathway is longer for species with larger head groups. The observation that conductance falls with increasing head group size (and therefore molecular length) supports the presence of tilted aromatic units. In contrast, 9-AMOC8S has a similar (Pt probe) or lower (graphene probe) conductance than BnOC8S, despite its larger head group. In this case, the position of the additional fused rings in the former system is such that the effective length of the molecule remains similar to the latter. The head group of 9-AMOC8S effectively becomes wider rather than longer and a reduction in conductance is not observed.

The second trend is the increase in the ratio between the conductance of Au|SAM|graphene junctions and the conductance of Au|SAM|Pt junctions (Table 1) as the number of fused rings in the ether-linked head group, *n*, increases. For MeOC8S (*n* = 0), BnOC8S (*n* = 1), 9-AMOC8S (*n* = 3) and PyrMOC8S (*n* = 4), the ratio *G*_graphene_/*G*_Pt_ is approximately equal to *n* + 1, indicating that the larger aromatics are more optimised to graphene contacts than Pt contacts. This observation can be attributed to π–π interactions,^52,82^ which are expected between aromatic species and graphene but not Pt. The presence of π–π interactions when a graphene electrode is used would favour a smaller average value of *θ* than for Pt, which the computational studies above indicate would result in increased conductance. For unsubstituted polyacenes adsorbed directly on graphene, the calculated binding energy increases linearly with the number of fused rings.^82^ Therefore, in the present systems, the strength of π–π interactions, and the concomitant reduction of *θ*, would be expected to increase as *n* increases. This accounts for the observed trend in *G*_graphene_/*G*_Pt_. Additionally, the atomically smooth graphene surface may accommodate conformational changes in the SAM more readily than the sharper Pt probe.

Two of the studied molecules do not follow the observed *G*_graphene_/*G*_Pt_ = *n* + 1 pattern. C8S is shorter than the other species and does not contain an ether linker, limiting its possible conformations in a SAM. Therefore, it is not unreasonable that C8S behaves differently to the other molecules. 2-AMOC8S shows a much smaller *G*_graphene_/*G*_Pt_ ratio (1.32) than would be expected based on observations for the remainder of the ether-linked series (Table 1). This may relate to the connectivity of the anthracene unit in 2-AMOC8S where in certain conformations the additional rings have a much larger effect on molecular length *versus*BnOC8S in comparison to 9-AMOC8S or PyrMOC8S. This geometric difference could impact SAM packing and the energy barrier associated with deviating from *θ* ≈ 90° in the presence of a top electrode. It is possible that related conformational effects are responsible for the relatively poor fit of the conductance data for 2-AMOC8S to a Gaussian curve in comparison to that of the other molecules in Fig. 2a.

The above observations indicate that polycyclic aromatic anchoring units have potential as graphene contacts in Au|SAM|graphene molecular junctions. The model systems studied in this work serve as a proof of concept and will inform design strategies for future SAM based devices. In order to best utilise the favourable interactions between aromatic species and graphene, more complex molecular designs are necessary. Achieving high molecular conductance will require consideration of factors such as: (i) how best to include large aromatic systems without significantly increasing molecular length; (ii) how to minimise *θ* while accounting for geometric requirements at the Au electrode and the desirability of conjugated molecular backbones. To minimise *θ*, it may prove important to reduce steric clash between neighbouring aromatic head groups. Methods to achieve this could include the use of large-footprint anchoring units^54,83^ on the gold surface to increase head group spacing, or the use of mixed monolayers containing functional species bearing aromatic contact units alongside simple “spacer” molecules.^84^

## Conclusions

The self-assembly and conductance properties of a series of alkanethiol derivatives bearing ether-linked aromatic head groups, and reference compounds, were investigated using AFM imaging, QCM studies and cAFM experiments. DFT and MD studies support the experimental observations. A sub-series of species with increasing alkyl linker length were observed to show an expected exponential conductance decay with increasing molecular length, with a tunnelling decay constant of *β* = 5.6 nm^−1^ in Au|SAM|Pt junctions and *β* = 3.5 nm^−1^ in Au|SAM|graphene junctions. Using both Pt and graphene top electrodes, conductance was observed to decrease as the size of the aromatic head group increased. This was attributed to an increasing junction length caused by the head groups being tilted with respect to the planar electrode, as larger head groups have a greater impact on the length of an extended molecule. The ratio of the conductances measured using graphene and Pt top electrodes, *G*_graphene_/*G*_Pt_, generally increased with the number of aromatic rings in the head group. As π–π interactions are known to increase in strength for progressively larger aromatic systems, this effect is attributed to an increasing tendency towards coplanarity with the graphene electrode as the size of the aromatic head group increases. These results indicate the effectiveness of aromatic head groups as contacts for graphene electrodes in asymmetric metal|SAM|graphene molecular junctions. Furthermore, they highlight some important considerations that must be made when designing optimised molecules for such junctions. The geometry of the head group is key; to maximise conductance it should lie near parallel to the graphene electrode in a molecular junction and have minimal impact on the length of the conductance pathway. These observations contribute to the design of the next generation of molecules for use in hybrid nanodevices.

## Author contributions

L. J. O. synthesised the molecules. X. W. and B. P.-J. performed device fabrication and measurements. M. J. carried out the calculations with assistance from H. S. The funding for this work was obtained by B. J. R., M. R. B. and C. J. L. who supervised the research and contributed to interpreting the results. L. J. O. and M. R. B. coordinated the writing of the manuscript, with contributions from all co-authors.

## Conflicts of interest

There are no conflicts to declare.

## Supplementary Material

NA-005-D2NA00873D-s001

## References

[cit1] Reed M. A., Zhou C., Muller C. J., Burgin T. P., Tour J. M. (1997). Science.

[cit2] Xu B., Tao N. J. (2003). Science.

[cit3] Li X., He J., Hihath J., Xu B., Lindsay S. M., Tao N. (2006). J. Am. Chem. Soc..

[cit4] Choi S. H., Kim B., Frisbie C. D. (2008). Science.

[cit5] Sun L., Diaz-Fernandez Y. A., Gschneidtner T. A., Westerlund F., Lara-Avila S., Moth-Poulsen K. (2014). Chem. Soc. Rev..

[cit6] Lambert C. J. (2015). Chem. Soc. Rev..

[cit7] Su T. A., Neupane M., Steigerwald M. L., Venkataraman L., Nuckolls C. (2016). Nat. Rev. Mater..

[cit8] Leary E., La Rosa A., González M. T., Rubio-Bollinger G., Agraït N., Martín N. (2015). Chem. Soc. Rev..

[cit9] Xiang D., Wang X., Jia C., Lee T., Guo X. (2016). Chem. Rev..

[cit10] Rincón-García L., Evangeli C., Rubio-Bollinger G., Agraït N. (2016). Chem. Soc. Rev..

[cit11] Wang K., Xu B. (2017). Top. Curr. Chem..

[cit12] Cui L., Miao R., Jiang C., Meyhofer E., Reddy P. (2017). J. Chem. Phys..

[cit13] Gehring P., Thijssen J. M., van der Zant H. S. J. (2019). Nat. Rev. Phys..

[cit14] Xin N., Guan J., Zhou C., Chen X., Gu C., Li Y., Ratner M. A., Nitzan A., Stoddart J. F., Guo X. (2019). Nat. Rev. Phys..

[cit15] Evers F., Korytár R., Tewari S., van Ruitenbeek J. M. (2020). Rev. Mod. Phys..

[cit16] O'Driscoll L. J., Bryce M. R. (2021). Nanoscale.

[cit17] Lambert C. J. , Quantum Transport in Nanostructures and Molecules, IOP Publishing, 2021

[cit18] Venkataraman L., Klare J. E., Nuckolls C., Hybertsen M. S., Steigerwald M. L. (2006). Nature.

[cit19] Haiss W., Wang C., Grace I., Batsanov A. S., Schiffrin D. J., Higgins S. J., Bryce M. R., Lambert C. J., Nichols R. J. (2006). Nat. Mater..

[cit20] Stefani D., Weiland K. J., Skripnik M., Hsu C., Perrin M. L., Mayor M., Pauly F., van der Zant H. S. J. (2018). Nano Lett..

[cit21] Jiang F., Trupp D. I., Algethami N., Zheng H., He W., Alqorashi A., Zhu C., Tang C., Li R., Liu J., Sadeghi H., Shi J., Davidson R., Korb M., Sobolev A. N., Naher M., Sangtarash S., Low P. J., Hong W., Lambert C. J. (2019). Angew. Chem., Int. Ed..

[cit22] Tang C., Tang Y., Ye Y., Yan Z., Chen Z., Chen L., Zhang L., Liu J., Shi J., Xia H., Hong W. (2020). Chem.

[cit23] Gorenskaia E., Naher M., Daukiya L., Moggach S. A., Costa Milan D., Vezzoli A., Lambert C. J., Nichols R. J., Becker T., Low P. J. (2021). Aust. J. Chem..

[cit24] Hong W., Manrique D. Z., Moreno-García P., Gulcur M., Mishchenko A., Lambert C. J., Bryce M. R., Wandlowski T. (2012). J. Am. Chem. Soc..

[cit25] Limburg B., Thomas J. O., Holloway G., Sadeghi H., Sangtarash S., Hou I. C.-Y., Cremers J., Narita A., Müllen K., Lambert C. J., Briggs G. A. D., Mol J. A., Anderson H. L. (2018). Adv. Funct. Mater..

[cit26] Herrer I. L., Ismael A. K., Milán D. C., Vezzoli A., Martín S., González-Orive A., Grace I., Lambert C., Serrano J. L., Nichols R. J., Cea P. (2018). J. Phys. Chem. Lett..

[cit27] Huang C., Chen S., Baruël Ørnsø K., Reber D., Baghernejad M., Fu Y., Wandlowski T., Decurtins S., Hong W., Thygesen K. S., Liu S.-X. (2015). Angew. Chem., Int. Ed..

[cit28] Wang G., Kim Y., Choe M., Kim T.-W., Lee T. (2011). Adv. Mater..

[cit29] Nijhuis C. A., Reus W. F., Whitesides G. M. (2009). J. Am. Chem. Soc..

[cit30] Gehring P., Sowa J. K., Cremers J., Wu Q., Sadeghi H., Sheng Y., Warner J. H., Lambert C. J., Briggs G. A. D., Mol J. A. (2017). ACS Nano.

[cit31] Planje I. J., Davidson R. J., Vezzoli A., Daaoub A., Sangtarash S., Sadeghi H., Martín S., Cea P., Lambert C. J., Beeby A., Higgins S. J., Nichols R. J. (2021). ACS Sens..

[cit32] Solomon G. C., Andrews D. Q., Hansen T., Goldsmith R. H., Wasielewski M. R., Van Duyne R. P., Ratner M. A. (2008). J. Chem. Phys..

[cit33] Yoshizawa K., Tada T., Staykov A. (2008). J. Am. Chem. Soc..

[cit34] Markussen T., Stadler R., Thygesen K. S. (2010). Nano Lett..

[cit35] Guédon C. M., Valkenier H., Markussen T., Thygesen K. S., Hummelen J. C., van der Molen S. J. (2012). Nat. Nanotechnol..

[cit36] Vazquez H., Skouta R., Schneebeli S., Kamenetska M., Breslow R., Venkataraman L., Hybertsen M. S. (2012). Nat. Nanotechnol..

[cit37] Arroyo C. R., Tarkuc S., Frisenda R., Seldenthuis J. S., Woerde C. H. M., Eelkema R., Grozema F. C., van der Zant H. S. J. (2013). Angew. Chem., Int. Ed..

[cit38] Manrique D. Z., Huang C., Baghernejad M., Zhao X., Al-Owaedi O. A., Sadeghi H., Kaliginedi V., Hong W., Gulcur M., Wandlowski T., Bryce M. R., Lambert C. J. (2015). Nat. Commun..

[cit39] O'Driscoll L. J., Bryce M. R. (2021). Nanoscale.

[cit40] O'Driscoll L. J., Sangtarash S., Xu W., Daaoub A., Hong W., Sadeghi H., Bryce M. R. (2021). J. Phys. Chem. C.

[cit41] Greenwald J. E., Cameron J., Findlay N. J., Fu T., Gunasekaran S., Skabara P. J., Venkataraman L. (2021). Nat. Nanotechnol..

[cit42] Chen H., Hou S., Wu Q., Jiang F., Zhou P., Zhang L., Jiao Y., Song B., Guo Q.-H., Chen X.-Y., Hong W., Lambert C. J., Stoddart J. F. (2021). Matter.

[cit43] Sangtarash S., Sadeghi H., Lambert C. J. (2018). Phys. Chem. Chem. Phys..

[cit44] Liu X., Sangtarash S., Reber D., Zhang D., Sadeghi H., Shi J., Xiao Z.-Y., Hong W., Lambert C. J., Liu S.-X. (2017). Angew. Chem., Int. Ed..

[cit45] Yang Y., Gantenbein M., Alqorashi A., Wei J., Sangtarash S., Hu D., Sadeghi H., Zhang R., Pi J., Chen L., Huang X., Li R., Liu J., Shi J., Hong W., Lambert C. J., Bryce M. R. (2018). J. Phys. Chem. C.

[cit46] Gantenbein M., Wang L., Al-jobory A. A., Ismael A. K., Lambert C. J., Hong W., Bryce M. R. (2017). Sci. Rep..

[cit47] Palomino-Ruiz L., Rodríguez-González S., Fallaque J. G., Márquez I. R., Agraït N., Díaz C., Leary E., Cuerva J. M., Campaña A. G., Martín F., Millán A., González M. T. (2021). Angew. Chem., Int. Ed..

[cit48] Grace I. M., Olsen G., Hurtado-Gallego J., Rincón-García L., Rubio-Bollinger G., Bryce M. R., Agraït N., Lambert C. J. (2020). Nanoscale.

[cit49] Jia C., Famili M., Carlotti M., Liu Y., Wang P., Grace I. M., Feng Z., Wang Y., Zhao Z., Ding M., Xu X., Wang C., Lee S.-J., Huang Y., Chiechi R. C., Lambert C. J., Duan X. (2018). Sci. Adv..

[cit50] Famili M., Jia C., Liu X., Wang P., Grace I. M., Guo J., Liu Y., Feng Z., Wang Y., Zhao Z., Decurtins S., Häner R., Huang Y., Liu S.-X., Lambert C. J., Duan X. (2019). Chem.

[cit51] Wang X., Bennett T. L. R., Ismael A., Wilkinson L. A., Hamill J., White A. J. P., Grace I. M., Kolosov O. V., Albrecht T., Robinson B. J., Long N. J., Cohen L. F., Lambert C. J. (2020). J. Am. Chem. Soc..

[cit52] Bailey S., Visontai D., Lambert C. J., Bryce M. R., Frampton H., Chappell D. (2014). J. Chem. Phys..

[cit53] Wei Z., Hansen T., Santella M., Wang X., Parker C. R., Jiang X., Li T., Glyvradal M., Jennum K., Glibstrup E., Bovet N., Wang X., Hu W., Solomon G. C., Nielsen M. B., Qiu X., Bjørnholm T., Nørgaard K., Laursen B. W. (2015). Adv. Funct. Mater..

[cit54] O'Driscoll L. J., Wang X., Jay M., Batsanov A. S., Sadeghi H., Lambert C. J., Robinson B. J., Bryce M. R. (2020). Angew. Chem., Int. Ed..

[cit55] Hybertsen M. S., Venkataraman L. (2016). Acc. Chem. Res..

[cit56] Zhao S., Wu Q., Pi J., Liu J., Zheng J., Hou S., Wei J., Li R., Sadeghi H., Yang Y., Shi J., Chen Z., Xiao Z., Lambert C., Hong W. (2020). Sci. Adv..

[cit57] van Veen F. H., Ornago L., van der Zant H. S. J., El Abbassi M. (2022). J. Phys. Chem. C.

[cit58] Mann B., Kuhn H. (1971). J. Appl. Phys..

[cit59] Ulman A., Eilers J. E., Tillman N. (1989). Langmuir.

[cit60] Haag R., Rampi M. A., Holmlin R. E., Whitesides G. M. (1999). J. Am. Chem. Soc..

[cit61] Vericat C., Vela M. E., Benitez G., Carro P., Salvarezza R. C. (2010). Chem. Soc. Rev..

[cit62] Subba Reddy B. V., Anusha B., Subba Reddy U. V., Yadav J. S., Suresh Reddy C. (2013). Helv. Chim. Acta.

[cit63] O'Driscoll L. J., Welsh D. J., Bailey S. W. D., Visontai D., Frampton H., Bryce M. R., Lambert C. J. (2015). Chem.–Eur. J..

[cit64] Garcia R., Martinez R. V., Martinez J. (2006). Chem. Soc. Rev..

[cit65] Amro N. A., Xu S., Liu G. Y. (2000). Langmuir.

[cit66] Barrena E., Palacios-Lidón E., Munuera C., Torrelles X., Ferrer S., Jonas U., Salmeron M., Ocal C. (2004). J. Am. Chem. Soc..

[cit67] Wei Z., Li T., Jennum K., Santella M., Bovet N., Hu W., Nielsen M. B., Bjørnholm T., Solomon G. C., Laursen B. W., Nørgaard K. (2012). Langmuir.

[cit68] Ning J., Li R., Shen X., Qian Z., Hou S., Rocha A. R., Sanvito S. (2007). Nanotechnology.

[cit69] Wang X., Ismael A., Almutlg A., Alshammari M., Al-Jobory A., Alshehab A., Bennett T. L. R., Wilkinson L. A., Cohen L. F., Long N. J., Robinson B. J., Lambert C. (2021). Chem. Sci..

[cit70] Frederiksen T., Munuera C., Ocal C., Brandbyge M., Paulsson M., Sanchez-Portal D., Arnau A. (2009). ACS Nano.

[cit71] Qi Y., Ratera I., Park J. Y., Ashby P. D., Quek S. Y., Neaton J. B., Salmeron M. (2008). Langmuir.

[cit72] Song H., Lee H., Lee T. (2007). J. Am. Chem. Soc..

[cit73] Burnham N. A., Colton R. J., Pollock H. M. (1992). Phys. Rev. Lett..

[cit74] Johnson K. L., Kendall K., Roberts A. D., Tabor D. (1971). Proc. R. Soc. London, Ser. A.

[cit75] Engelkes V. B., Beebe J. M., Frisbie C. D. (2004). J. Am. Chem. Soc..

[cit76] He C., Zhang Q., Gao T., Liu C., Chen Z., Zhao C., Zhao C., Nichols R. J., Dappe Y. J., Yang L. (2020). Phys. Chem. Chem. Phys..

[cit77] Zhang Q., Liu L., Tao S., Wang C., Zhao C., González C., Dappe Y. J., Nichols R. J., Yang L. (2016). Nano Lett..

[cit78] Soler J. M., Artacho E., Gale J. D., García A., Junquera J., Ordejón P., Sánchez-Portal D. (2002). J. Phys.: Condens. Matter.

[cit79] Ferrer J., Lambert C. J., García-Suárez V. M., Manrique D. Z., Visontai D., Oroszlany L., Rodríguez-Ferradás R., Grace I., Bailey S. W. D., Gillemot K., Sadeghi H., Algharagholy L. A. (2014). New J. Phys..

[cit80] Sangtarash S., Huang C., Sadeghi H., Sorohhov G., Hauser J., Wandlowski T., Hong W., Decurtins S., Liu S.-X., Lambert C. J. (2015). J. Am. Chem. Soc..

[cit81] Bai J., Daaoub A., Sangtarash S., Li X., Tang Y., Zou Q., Sadeghi H., Liu S., Huang X., Tan Z., Liu J., Yang Y., Shi J., Mészáros G., Chen W., Lambert C., Hong W. (2019). Nat. Mater..

[cit82] Li Y., Tu X., Wang H., Sanvito S., Hou S. (2015). J. Chem. Phys..

[cit83] Valášek M., Mayor M. (2017). Chem.–Eur. J..

[cit84] Kumar S., van Herpt J. T., Gengler R. Y. N., Feringa B. L., Rudolf P., Chiechi R. C. (2016). J. Am. Chem. Soc..

