# Supplementary material for: Planar aromatic anchors control the electrical conductance of gold|molecule|graphene junctions
Source: Nanoscale Adv. 2023 Mar 27;5(8):2299–306. doi: 10.1039/d2na00873d (PMC10089101; doi:10.1039/d2na00873d)
Supplement: NA-005-D2NA00873D-s001 [file NA-005-D2NA00873D-s001.pdf]

Electronic Supplementary Material (ESI) for Nanoscale Advances.
This journal is © The Royal Society of Chemistry 2023

Supplementary Information

Planar aromatic anchors control the electrical conductance of gold|molecule|graphene junctions

Luke J. O'Driscoll, Michael Jay, Benjamin J. Robinson, Hatef Sadeghi, Xintai Wang, Becky Penhale-Jones, Martin R. Bryce, and Colin J. Lambert

S1. Chemical Synthesis

S1.1 General Experimental Details

Reagents were purchased commercially from Sigma Aldrich, Fluorochem or TCI and used as received unless otherwise stated. Anhydrous solvents were prepared using an Innovative Technology solvent purification system and stored in ampoules under argon, and anhydrous triethylamine was purchased commercially and used as received. Thin-layer chromatography (TLC) analysis was carried out using Merck silica gel 60 F_{254} TLC plates and spots were visualised using a UV lamp emitting at 365 or 254 nm. Column chromatography was performed using silica gel 60A (40-63 µm) purchased from Fluorochem. ^{1}H and ^{13}C NMR spectroscopy was carried out on a Bruker AV400 NMR spectrometer. For ^{1}H NMR spectra, chemical shifts are reported relative to the residual solvent peak (7.26 ppm for $CHCl_3$) and for ^{13}C NMR spectra, chemical shifts are reported relative to the solvent peak (77.16 ppm for $CDCl_3$). These, and any other residual solvent peaks were referenced to values reported in the literature.[1] All NMR spectra were processed using MestReNova V12. ESI mass spectrometry was carried out using a TQD mass spectrometer (Waters Ltd, UK) in flow injection analysis mode with acetonitrile as the mobile phase, subsequent accurate mass measurements used a QtoF Premier mass spectrometer (Waters Ltd, UK). ASAP mass spectrometry (including accurate mass measurements) was carried out using an LCT Premier XE mass spectrometer (Waters Ltd, UK) using TOF detection. Exact mass measurements were processed using Elemental Composition 4.0 embedded within MassLynx 4.1 (Waters Ltd, UK). Melting points were determined using a Stuart SMP40 machine with a ramping rate of 3 °C min^{-1}. The videos produced by the machine were analysed manually to determine the melting point.

Unless otherwise stated, reactions were conducted under an argon atmosphere. Glassware was first dried under vacuum using a heat gun, then filled directly with argon. Solvents and liquid reagents were added by syringe or cannula, and solid reagents were added under a positive pressure of argon. Degassing was conducted by bubbling argon through the reaction mixture using an argon-filled balloon fitted with a syringe needle. All mixed solvents in this work were prepared as v/v mixtures.

S1.2 Nomenclature

The naming scheme described in the main text is extended here to synthetic intermediates. Compound names begin with the (proto-)head group (using AM and PyrM to refer to anthracenemethylene and pyrenemethylene groups, respectively), followed by its substituent. With the exception of benzylic bromides prepared at the beginning of the synthetic route, the substituents are ether-linked alkyl chains bearing a terminal functionality, and are described in the format "OC*n*X,"

where O represents the ether linkage, C*n* indicates the number of carbons in the alkyl chain, and X is the terminal functionality (e.g. OH, OMs, SAc). For example, 2-AMOC8OMs is an octyl chain substituted at each terminus, at one with a 2-(anthracenemethyl) ether and the other with a mesylate functionality.

S1.3 Synthetic Schemes

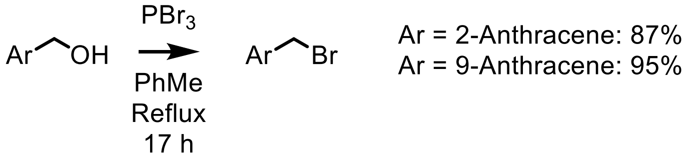

Scheme S1. Preparation of isomeric anthracenemethyl bromides.

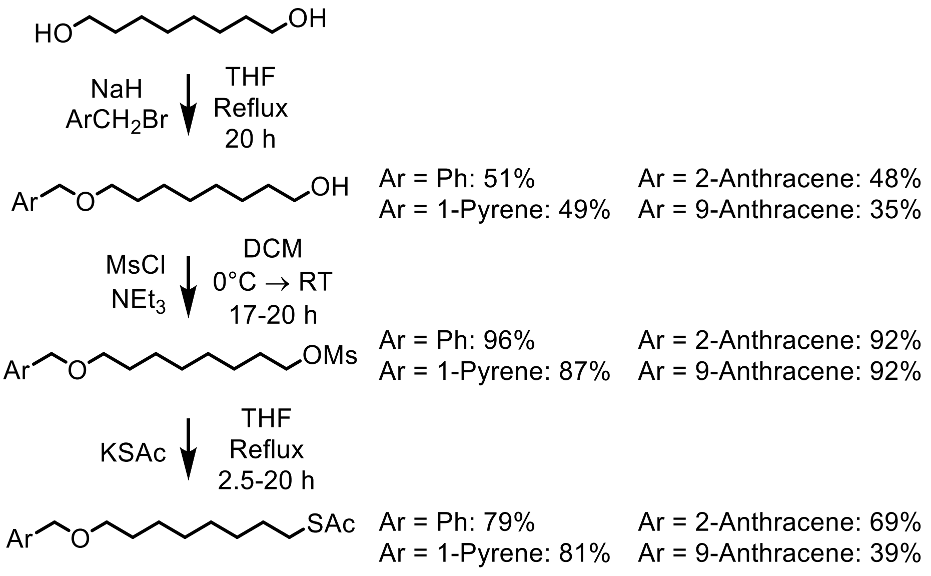

Scheme S2. Preparation of protected octanethiol derivatives with arylmethyl ether head groups.

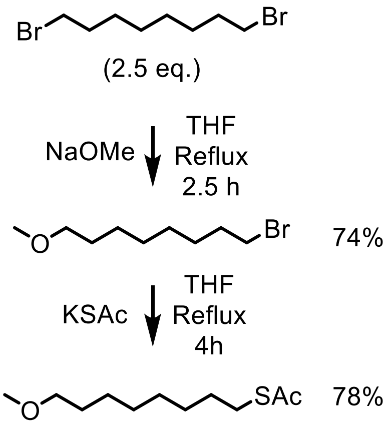

Scheme S3. Preparation of protected octanethiol derivative with methoxy head group.

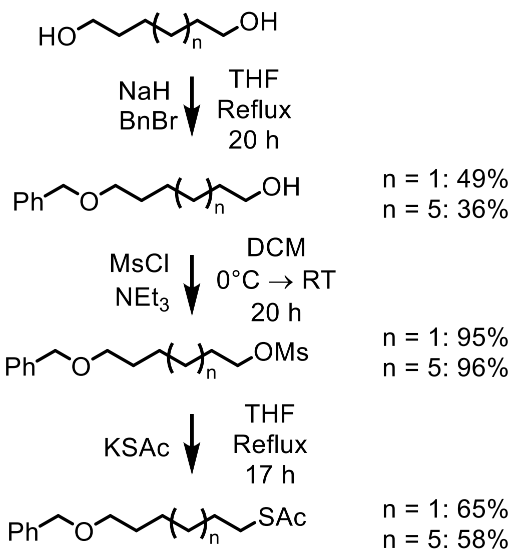

Scheme S4. Preparation of hexyl and decyl analogues of protected alkanethiols with a benzyl ether head group.

S1.4 Synthetic Procedures

S1.4.1 – Preparation of Arylmethyl Bromides

1-pyrenemethyl bromide was prepared according to a literature procedure.[2]

2-AMBr

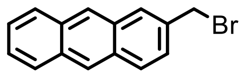

This synthesis was based on a literature synthesis of 1-pyrenemethyl bromide.[2]

A solution of 2-anthracenemethanol (550 mg, 2.64 mmol, 1 eq.) in anhydrous toluene (40 mL) was prepared in a dry flask under argon at RT. Phosphorus tribromide (0.1 mL, 1.06 mmol, 0.4 eq.) was added to the stirred solution and the reaction was then heated at reflux for 17 h. After cooling to RT the reaction was diluted with Et_2O (50 mL) and washed with deionized water (3 × 50 mL). Some insoluble material remained in the aqueous layers, so these were combined and extracted with DCM (2 × 25 mL). These organic layers were combined with the Et_2O/toluene layer then dried ($MgSO_4$) before the solvent was removed *in vacuo* to afford **2-AMBr** as a yellow solid of sufficient purity for the next reaction* (625 mg, 87%). The ^{1}H NMR spectrum was in agreement with a previous report.[3]

^{1}H NMR (400 MHz, $CDCl_3$) δ 8.40 (d, *J* = 5.9 Hz, 2H), 8.03 – 7.95 (m, 4H), 7.52 – 7.46 (m, 3H), 4.71 (s, 2H).

^{13}C NMR (101 MHz, $CDCl_3$) δ 134.6, 132.3, 132.2, 131.3, 131.2, 129.4, 128.34, 128.32, 128.0, 126.7, 126.43, 126.38, 125.9, 125.8, 34.7.

---

* No further purification was attempted as instability had been observed for the pyrene analogue.

MS: ASAP m/z: 273.0 $[M+H]^+$ (^{81}Br), 271.0 $[M+H]^+$ (^{79}Br), 191.1 $[C_{15}H_{11}]^{+*}$

HRMS: m/z $[M^+]^{+\cdot}$ calculated for $C_{15}H_{11}{}^{79}Br$, 270.0044; found, 270.0067

9-AMBr

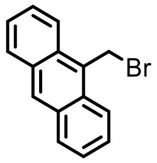

Prepared according to the above method for **2-AMBr** on the same scale using 9-anthracenemethanol in place of 2-anthracenemethanol. **9-AMBr** was obtained as a yellow solid of sufficient purity for the next reaction† (680 mg, 95%). The ^1H NMR spectrum was in agreement with a previous report.[4]

^1H NMR (400 MHz, $CDCl_3$) δ 8.50 (s, 1H), 8.31 (apparent dq, *J* = 8.9, 1.0 Hz, 2H), 8.05 (apparent ddt, *J* = 8.5, 1.4, 0.7 Hz, 2H), 7.65 (ddd, *J* = 8.9, 6.6, 1.4 Hz, 2H), 7.51 (ddd, *J* = 8.5, 6.6, 1.0 Hz, 2H), 5.55 (s, 2H).

^{13}C NMR (101 MHz, $CDCl_3$) δ 131.7, 129.9, 129.4, 129.3, 128.0, 126.9, 125.5, 123.6, 27.1.

MS: ASAP m/z: 273.0 $[M+H]^+$ (^{81}Br), 271.0 $[M+H]^+$ (^{79}Br), 191.1 $[C_{15}H_{11}]^{+‡}$

HRMS: m/z $[M^+]^{+\cdot}$ calculated for $C_{15}H_{11}{}^{79}Br$, 270.0044; found, 270.0059

S1.4.2 – Monosubstitution of diols

BnOC8OH

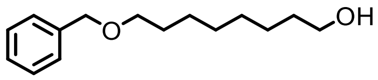

This synthesis was adapted from a literature procedure.[5] Attempts to use the previously published conditions, in which the reaction was not heated, returned only starting materials based on TLC analysis and NMR spectroscopy of the crude product.

Sodium hydride (95 wt. %, 3.37 g, 134 mmol, 2.05 eq.) was dispersed in anhydrous THF (150 mL) in a dry flask under argon and heated to 40 °C. A solution of 1,8-octanediol (10.0 g, 68.4 mmol, 1.05 eq.) in anhydrous THF was prepared in a second dry flask under argon, then added to the NaH dispersion via cannula. The mixture was stirred at 40 °C for 1 h before benzyl bromide (7.75 mL, 65.2 mmol, 1 eq.) was added and the reaction was heated at reflux for 20 h. After cooling to RT, the reaction was quenched by careful addition of sat. $NH_4Cl_{(aq)}$ (150 mL) then extracted with DCM (3 × 150 mL). The combined organic layers were washed with deionized water (150 mL) and brine (150 mL) then dried ($MgSO_4$) before the solvent was removed *in vacuo*. The resulting yellow oil was purified by column chromatography (5 cm Ø, 500 mL SiO_2, eluent: 2:1 hexane/EtOAc) to afford **BnOC8OH** as a pale yellow oil (7.87 g, 51%). NMR spectra were in accordance with those reported previously.[5]

---

* i.e. fragmentation to form a 2-methylanthracene cation

† No further purification was attempted as instability had been observed for the pyrene analogue.

‡ i.e. fragmentation to form a 9-methylanthracene cation

^{1}H NMR (400 MHz, $CDCl_3$) δ 7.39 – 7.30 (m, 4H), 7.31 – 7.26 (m, 1H), 4.50 (s, 2H), 3.63 (td, *J* = 6.6, 5.2 Hz, 2H), 3.46 (t, *J* = 6.6 Hz, 2H), 1.66 – 1.59 (m, 2H), 1.59 – 1.52 (m, 2H), 1.42 – 1.28 (m, 8H), 1.26 (t, *J* = 5.2 Hz, 1H).

^{13}C NMR (101 MHz, $CDCl_3$) δ 138.8, 128.5, 127.8, 127.6, 73.0, 70.6, 63.2, 32.9, 29.9, 29.6, 29.5, 26.3, 25.8.

PyrMOC8OH

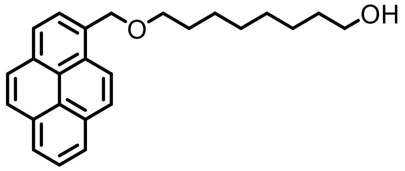

Sodium hydride (95 wt. %, 170 mg, 6.73 mmol, 2.0 eq.) was dispersed in anhydrous THF (15 mL) in a dry flask under argon and heated to 40 °C. A solution of 1,8-octanediol (520 mg, 3.56 mmol, 1.05 eq.) in anhydrous THF was prepared in a second dry flask under argon, then added to the NaH dispersion via cannula. The mixture was heated at reflux for 1 h before 1-pyrenemethyl bromide[2] (1.00 g, 3.39 mmol, 1 eq.) was added and the reaction was heated at reflux for 20 h. After cooling to RT, the reaction was quenched by careful addition of sat. $NH_4Cl_{(aq)}$ (25 mL) then extracted with DCM (3 × 50 mL). The combined organic layers were washed with deionized water (75 mL) and brine (75 mL) then dried ($MgSO_4$) before the solvent was removed *in vacuo*. The resulting mixture of yellow solid and yellow oil was purified by column chromatography (5 cm Ø, 250 mL SiO_2, gradient elution from 1:1 hexane/DCM to DCM,[*] then from DCM to 96:4 DCM/MeOH) to afford **PyrMOC8OH** as a pale yellow solid (599 mg, 49%).

^{1}H NMR (400 MHz, $CDCl_3$) δ 8.38 (d, *J* = 9.2 Hz, 1H), 8.22 – 8.17 (m[†], 2H), 8.17 – 8.12 (m[‡], 2H), 8.05 (s, 2H), 8.04 – 7.98 (m[§], 2H), 5.21 (s, 2H), 3.60 (t, *J* = 6.6 Hz, 2H), 3.57 (t, *J* = 6.7 Hz, 2H), 1.71 – 1.62 (m, 2H), 1.53 – 1.44 (m, 2H), 1.42 – 1.33 (m, 2H), 1.31 – 1.21 (m, 7H).

^{13}C NMR (101 MHz, $CDCl_3$) δ 132.0, 131.4, 131.3, 131.0, 129.5, 127.7, 127.6, 127.4, 127.1, 126.0, 125.29, 125.27, 125.1, 124.9, 124.6, 123.7, 71.6, 70.6, 63.1, 32.9, 29.9, 29.5[**], 26.3, 25.7.

MS: ASAP m/z: 360.2 $[M^+]^{+\cdot}$, 359.2 $[M\text{-}H^-]^+$, 215.1 $[C_{17}H_{11}]^{+}$[††]

HRMS: m/z $[M^+]^{+\cdot}$ calculated for $C_{25}H_{28}O_2$, 360.2089; found, 360.2086

m.p.: 51.5 – 53 °C

[*] This eluted a yellow band, found to be a doubly-substituted by-product based on ^{1}H NMR spectroscopy

[†] This signal appears to be two overlapping triplets each with *J* = ca. 7.4 Hz

[‡] This signal appears to be two overlapping doublets with *J* = ca. 7.7 Hz and *J* = ca. 9.2 Hz

[§] This signal appears to be an overlapping doublet (*J* = ca. 7.8 Hz) and triplet (*J* = ca. 7.6 Hz)

[**] This is believed to be two overlapping peaks

[††] i.e. fragmentation to form a 1-methylpyrene cation

2-AMOC8OH

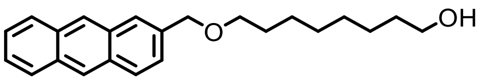

1,8-octanediol (311 mg, 2.13 mmol, 1.05 eq.) was dissolved in anhydrous THF (20 mL) in a dry flask under argon at RT. Sodium hydride (100 mg, 4.17 mmol, 2.05 eq.) was added and the mixture was then heated at reflux for 1 h before **2-AMBr** (550 mg, 2.03 mmol, 1 eq.) was added and the mixture heated at reflux for 20 h. After cooling to RT, the reaction was quenched by careful addition of sat. $NH_4Cl_{(aq)}$ (25 mL) then extracted with DCM (3 × 25 mL). The combined organic layers were washed with deionized water (50 mL) and brine (50 mL) then dried ($MgSO_4$) before the solvent was removed *in vacuo*. The resulting yellow solid was purified by column chromatography (5 cm Ø, 150 mL SiO_2, gradient elution from DCM to 98:2 DCM/MeOH) to afford **2-AMOC8OH** as a yellow solid (325 mg, 48%).

^{1}H NMR (400 MHz, $CDCl_3$) δ 8.41 (d, *J* = 3.0 Hz, 2H), 8.03 – 7.96 (m, 3H), 7.92 (s, 1H), 7.49 – 7.43 (m, 3H), 4.69 (s, 2H), 3.62 (t, *J* = 6.6 Hz, 2H), 3.54 (t, *J* = 6.6 Hz, 2H), 1.71 – 1.62 (m, 2H), 1.58 – 1.51 (m, 2H), 1.45 – 1.37 (m, 2H), 1.37 – 1.28 (m, 7H).

^{13}C NMR (101 MHz, $CDCl_3$) δ 135.8, 132.0, 131.8, 131.6, 131.4, 128.6, 128.32, 128.26 126.28, 126.27, 126.2, 125.7, 125.5, 125.4, 73.2, 70.7, 63.2, 32.9, 29.9, 29.6, 29.5, 26.3, 25.8.

MS: ASAP m/z: 336.2 $[M^+]^{+\cdot}$, 335.2 $[M\text{-}H^-]^+$, 191.1 $[C_{15}H_{11}]^{+}$*

HRMS: m/z $[M\text{-}H^-]^+$ calculated for $C_{23}H_{27}O_2$, 335.2011; found, 335.2018

m.p.: 125 – 127 °C

9-AMOC8OH

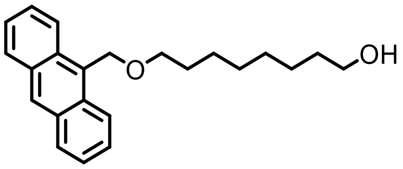

1,8-octanediol (311 mg, 2.13 mmol, 1.05 eq.) was dissolved in anhydrous THF (20 mL) in a dry flask under argon at RT. Sodium hydride (100 mg, 4.17 mmol, 2.05 eq.) was added and the mixture was then heated at reflux for 1 h before **9-AMBr** (550 mg, 2.03 mmol, 1 eq.) was added and the mixture heated at reflux for 20 h. After cooling to RT, the reaction was quenched by careful addition of sat. $NH_4Cl_{(aq)}$ (25 mL) then extracted with DCM (3 × 25 mL). The combined organic layers were washed with deionized water (50 mL) and brine (50 mL) then dried ($MgSO_4$) before the solvent was removed *in vacuo*. The resulting yellow-brown oil (containing some solid) was purified by column chromatography (5 cm Ø, 150 mL SiO_2, gradient elution from DCM to 98:2 DCM/MeOH) to afford **9-AMOC8OH** as a viscous yellow oil (241 mg, 35%).

* i.e. fragmentation to form a 2-methylanthracene cation

^{1}H NMR (400 MHz, $CDCl_3$) δ 8.46 (s, 1H), 8.39 (d[*], *J* = 8.9 Hz, 2H), 8.01 (d[†], *J* = 8.4 Hz, 2H), 7.54 (ddd, *J* = 8.9, 6.5, 1.4 Hz, 2H), 7.47 (ddd, *J* = 8.4, 6.5, 1.1 Hz, 2H), 5.46 (s, 2H), 3.67 (t, *J* = 6.5 Hz, 2H), 3.61 (t, *J* = 6.6 Hz, 2H), 1.68 – 1.59 (m, 2H), 1.56 – 1.48 (m, 2H), 1.39 – 1.32 (m, 2H), 1.31 – 1.23 (m, 7H).

^{13}C NMR (101 MHz, $CDCl_3$) δ 131.6, 131.1, 129.2, 129.1, 128.4, 126.2, 125.1, 124.6, 70.8, 65.1, 63.2, 32.9, 30.0, 29.5[‡], 26.3, 25.8.

MS: ASAP m/z: 336.2 $[M^{+}]^{+\cdot}$, 335.2 $[M\text{-}H^{-}]^{+}$, 191.1 $[C_{15}H_{11}]^{+}$[§]

HRMS: m/z $[M\text{-}H^{-}]^{+}$ calculated for $C_{23}H_{27}O_2$, 335.2011; found, 335.2019

BnOC6OH

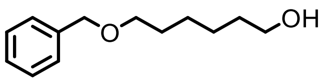

1,6-hexanediol (522 mg, 4.42 mmol, 1.05 eq.) was dissolved in anhydrous THF (30 mL) in a dry flask under argon at RT. Sodium hydride (207 mg, 8.63 mmol, 2.05 eq.) was added and the mixture was then heated at reflux for 1 h before benzyl bromide (0.5 mL, 4.21 mmol, 1 eq.) was added and the mixture heated at reflux for 20 h. After cooling to RT, the reaction was quenched by careful addition of sat. $NH_4Cl_{(aq)}$ (25 mL) then extracted with DCM (3 × 25 mL). The combined organic layers were washed with deionized water (50 mL) and brine (50 mL) then dried ($MgSO_4$) before the solvent was removed *in vacuo*. The resulting yellow oil was purified by column chromatography (5 cm Ø, 150 mL SiO_2, eluent: 2:1 hexane/EtOAc) to afford **BnOC6OH** as a pale yellow oil (430 mg, 49%). NMR spectra were in accordance with those reported previously.[6]

^{1}H NMR (400 MHz, $CDCl_3$) δ 7.37 – 7.32 (m, 4H), 7.31 – 7.26 (m, 1H), 4.50 (s, 2H), 3.63 (t, *J* = 6.5 Hz, 2H), 3.47 (t, *J* = 6.6 Hz, 2H), 1.68 – 1.60 (m, 2H), 1.60 – 1.53 (m, 2H), 1.46 – 1.32 (m, 4H), 1.30 (bs, 1H).

^{13}C NMR (101 MHz, $CDCl_3$) δ 138.8, 128.5, 127.8, 127.6, 73.0, 70.5, 63.1, 32.8, 29.9, 26.1, 25.7.

BnOC10OH

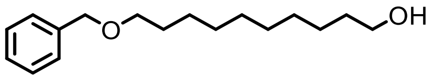

1,10-decanediol (770 mg, 4.42 mmol, 1.05 eq.) was dissolved in anhydrous THF (30 mL) in a dry flask under argon at RT. Sodium hydride (207 mg, 8.63 mmol, 2.05 eq.) was added and the mixture was then heated at reflux for 1 h before benzyl bromide (0.5 mL, 4.21 mmol, 1 eq.) was added and the mixture heated at reflux for 20 h. (An equipment failure meant that the reaction boiled dry during this time, but TLC analysis indicated that the reaction had proceeded as expected.) After cooling to RT, the reaction mixture was redissolved in THF (30 mL), treated with sat. $NH_4Cl_{(aq)}$ (25 mL), then extracted

[*] Additional coupling is evident, but not fully resolved at this frequency – we believe the signal is a ddd with *J* = 8.9, 1.1, and <1 Hz.
[†] Additional coupling is evident, but not fully resolved at this frequency – we believe the signal is a ddd with *J* = 8.4, 1.4, and <1 Hz, possibly with an additional low *J* coupling.
[‡] This is believed to be two overlapping peaks
[§] i.e. fragmentation to form a 9-methylanthracene cation

with DCM (3 × 25 mL). The combined organic layers were washed* with deionized water (50 mL) and brine (50 mL) then dried ($MgSO_4$) before the solvent was removed *in vacuo*. The resulting mixture of yellow oil and white solid was purified by column chromatography (5 cm Ø, 200 mL SiO_2, eluent: 2:1 hexane/EtOAc) to afford **BnOC10OH** as a pale yellow oil (399 mg, 36%). NMR spectra were in accordance with those reported previously.[7, 8]

^{1}H NMR (400 MHz, $CDCl_3$) δ 7.38 – 7.31 (m, 4H), 7.31 – 7.26 (m, 1H), 4.50 (s, 2H), 3.63 (t, *J* = 6.7 Hz, 2H), 3.46 (t, *J* = 6.6 Hz, 2H), 1.66 – 1.51 (m, 4H), 1.40 – 1.26 (m, 12H), 1.25 (bs, 1H).

^{13}C NMR (101 MHz, $CDCl_3$) δ 138.8, 128.5, 127.8, 127.6, 73.0, 70.7, 63.2, 32.9, 29.9, 29.67, 29.65, 29.6, 29.5, 26.3, 25.9.

S1.4.3 - Mesylations

BnOC8OMs

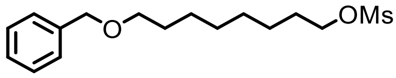

A flask containing **BnOC8OH** (7.87 g, 33.3 mmol, 1 eq.) was dried *in vacuo* overnight then filled directly with argon. Anhydrous DCM (70 mL) was added, affording a solution to which anhydrous Et_3N (4.7 mL, 33.7 mmol, 1.01 eq.) was added before the mixture was cooled to 0 °C with an ice-water bath. MsCl (2.6 ml, 33.6 mmol, 1.01 eq.) was then added, inducing rapid precipitate formation. The mixture was stirred for 17 h, warming slowly to RT, then washed with sat. $NaHCO_{3(aq)}$ (2 × 50 mL), deionised water (2 × 50 mL) and brine (50 mL). The organic layer was dried ($MgSO_4$) before the solvent was removed *in vacuo* to afford **BnOC8OMs** as a yellow-orange oil (10.1 g, 96%) of sufficient purity to use in the following step with no further purification.

^{1}H NMR (400 MHz, $CDCl_3$) δ 7.37 – 7.32 (m, 4H), 7.31 – 7.26 (m, 1H), 4.50 (s, 2H), 4.22 (t, *J* = 6.6 Hz, 2H), 3.46 (t, *J* = 6.6 Hz, 2H), 2.99 (s, 3H), 1.79 – 1.70 (m, 2H), 1.66 – 1.57 (m, 2H), 1.44 – 1.29 (m, 8H).

^{13}C NMR (101 MHz, $CDCl_3$) δ 138.8, 128.5, 127.8, 127.6, 73.0, 70.5, 70.3, 37.5, 29.8, 29.4, 29.2, 29.1, 26.2, 25.5.

MS: ESI$^+$ m/z: 337.2 $[M+Na]^+$, 332.2 $[M+NH_4]^+$, 315.4 $[M+H]^+$

HRMS: m/z: $[M+H]^+$ calculated for $C_{16}H_{27}O_4S$, 315.1630; found, 315.1628

PyrMOC8OMs

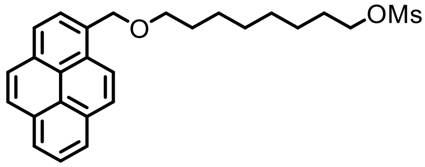

PyrMOC8OH (500 mg, 1.39 mmol, 1 eq.) was dissolved in anhydrous DCM (20 mL) in a dry flask under argon. Anhydrous Et_3N (0.20 mL, 1.43 mmol, 1.03 eq.) was added and the mixture was cooled to 0 °C

* N.B. Emulsion formation meant layer separation took much longer (>10 min) than for the hexyl and octyl analogues.

with an ice-water bath before MsCl (0.11 ml, 1.42 mmol, 1.02 eq.) was added. The mixture was stirred for 22 h, warming slowly to RT, then washed with sat. $NaHCO_{3(aq)}$ (2 × 25 mL), deionised water (2 × 25 mL) and brine (25 mL). The organic layer was dried ($MgSO_4$) before the solvent was removed *in vacuo* to afford **PyrMOC8OMs** as a yellow solid (528 mg, 87%) of sufficient purity to use in the following step with no further purification.

^{1}H NMR (400 MHz, $CDCl_3$) δ 8.39 (d, *J* = 9.2 Hz, 1H), 8.23 – 8.17 (m[*], 2H), 8.17 – 8.12 (m[†], 2H), 8.06 (s, 2H), 8.05 – 7.99 (m[‡], 2H), 5.22 (s, 2H), 4.15 (t, *J* = 6.6 Hz, 2H), 3.60 (t, *J* = 6.5 Hz, 2H), 2.96 (s, 3H), 1.70 – 1.60 (m, 4H), 1.41 – 1.33 (m, 2H), 1.33 – 1.22 (m, 6H[§]).

^{13}C NMR (101 MHz, $CDCl_3$) δ 132.0, 131.40, 131.37 131.0, 129.5, 127.7, 127.6, 127.5, 127.1, 126.1, 125.3[**], 125.1, 124.9, 124.6, 123.7, 71.7, 70.5, 70.3, 37.5, 29.9, 29.3, 29.2, 29.1, 26.2, 25.4.

MS: ASAP m/z: 438.2 $[M^{+}]^{+\cdot}$, 437.2 $[M\text{-}H^{-}]^{+}$, 215.1 $[C_{17}H_{11}]^{+}$[††]

HRMS: m/z $[M^{+}]^{+\cdot}$ calculated for $C_{26}H_{30}O_4S$, 438.1865; found, 438.1864

m.p.: 87.5 – 88.5 °C[‡‡]

2-AMOC8OMs

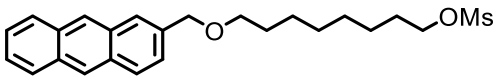

A flask containing **2-AMOC8OH** (292 mg, 0.867 mmol, 1 eq.) was dried *in vacuo* for ca. 2 h then filled directly with argon. Anhydrous DCM (20 mL) was added, affording a solution to which anhydrous Et_3N (0.13 mL, 0.93 mmol, 1.07 eq.) was added before the mixture was cooled to 0 °C with an ice-water bath. MsCl (0.07 ml, 0.90 mmol, 1.04 eq.) was then added and the mixture was stirred for 17 h, warming slowly to RT. After this time the mixture was washed with sat. $NaHCO_{3(aq)}$ (2 × 25 mL), deionised water (2 × 25 mL) and brine (25 mL). The organic layer was dried ($MgSO_4$) before the solvent was removed *in vacuo* to afford **2-AMOC8OMs** as a waxy yellow solid (330 mg, 92%) of sufficient purity to use in the following step with no further purification.

^{1}H NMR (400 MHz, $CDCl_3$) δ 8.41 (d, *J* = 3.7 Hz, 2H), 8.03 – 7.96 (m, 3H), 7.92 (s, 1H), 7.50 – 7.42 (m, 3H), 4.69 (s, 2H), 4.20 (t, *J* = 6.6 Hz, 2H), 3.54 (t, *J* = 6.6 Hz, 2H), 2.98 (s, 3H), 1.77 – 1.69 (m, 2H), 1.69 – 1.61 (m, 2H), 1.45 – 1.31 (m, 8H).

^{13}C NMR (101 MHz, $CDCl_3$) δ 135.7, 132.0, 131.8, 131.6, 131.4, 128.6, 128.32, 128.25, 126.29, 126.27, 126.2, 125.7, 125.54, 125.45, 73.3, 70.6, 70.3, 37.5, 29.9, 29.4, 29.2, 29.1, 26.2, 25.5.

MS: ASAP m/z: 414.2 $[M^{+}]^{+\cdot}$, 413.2 $[M\text{-}H^{-}]^{+}$, 191.1 $[C_{15}H_{11}]^{+}$[§§]

HRMS: m/z $[M+H]^{+}$ calculated for $C_{24}H_{30}O_4S$, 414.1865; found, 414.1858

[*] This signal appears to be two overlapping triplets each with *J* = ca. 7.4 Hz
[†] This signal appears to be two overlapping doublets with *J* = ca. 7.7 Hz and *J* = ca. 9.2 Hz
[‡] This signal appears to be an overlapping doublet (*J* = ca. 7.8 Hz) and triplet (*J* = ca. 7.6 Hz)
[§] The integral of this signal is closer to 7, the remainder is attributed to 'H-grease'
[**] This is believed to be two overlapping peaks
[††] i.e. fragmentation to form a 1-methylpyrene cation
[‡‡] A transition to an intermediate phase is also observed at 78 – 80 °C.
[§§] i.e. fragmentation to form a 2-methylanthracene cation

m.p.: 97 – 99 °C

9-AMOC8OMs

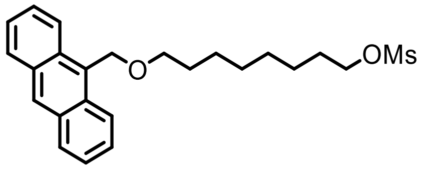

A flask containing **9-AMOC8OH** (209 mg, 0.621 mmol, 1 eq.) was dried *in vacuo* for ca. 2 h then filled directly with argon. Anhydrous DCM (15 mL) was added, affording a solution to which anhydrous Et_3N (0.09 mL, 0.65 mmol, 1.05 eq.) was added before the mixture was cooled to 0 °C with an ice-water bath. MsCl (0.05 ml, 0.65 mmol, 1.05 eq.) was then added and the mixture was stirred for 17 h, warming slowly to RT. After this time the mixture was washed with sat. $NaHCO_{3(aq)}$ (2 × 20 mL), deionised water (2 × 20 mL) and brine (20 mL). The organic layer was dried ($MgSO_4$) before the solvent was removed *in vacuo* to afford **9-AMOC8OMs** as a viscous yellow oil (237 mg, 92%) of sufficient purity to use in the following step with no further purification.

^{1}H NMR (400 MHz, $CDCl_3$) δ 8.46 (s, 1H), 8.39 (d[*], *J* = 8.9 Hz, 2H), 8.01 (d[†], *J* = 8.4 Hz, 2H), 7.54 (ddd, *J* = 8.9, 6.5, 1.4 Hz, 2H), 7.47 (ddd, *J* = 8.4, 6.5, 1.1 Hz, 2H), 5.46 (s, 2H), 4.18 (t, *J* = 6.6 Hz, 2H), 3.67 (t, *J* = 6.5 Hz, 2H), 2.97 (s, 3H), 1.74 – 1.66 (m, 2H), 1.66 – 1.59 (m, 2H), 1.37 – 1.23 (m, 8H[‡]).

^{13}C NMR (101 MHz, $CDCl_3$) δ 131.5, 131.0, 129.1, 129.0, 128.3, 126.1, 124.9, 124.4, 70.6, 70.2, 65.0, 37.4, 29.8, 29.12, 29.06, 28.9, 26.1, 25.3.

MS: ASAP m/z: 414.2 $[M^+]^{+\cdot}$, 413.2 $[M\text{-}H^-]^+$, 191.1 $[C_{15}H_{11}]^{+}$[§]

HRMS: m/z $[M+H]^+$ calculated for $C_{24}H_{30}O_4S$, 414.1865; found, 414.1855

BnOC6OMs

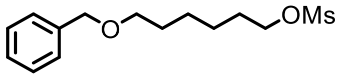

A flask containing **BnOC6OH** (425 mg, 2.04 mmol, 1 eq.) was dried *in vacuo* for ca. 2 h then filled directly with argon. Anhydrous DCM (30 mL) was added, affording a solution to which anhydrous Et_3N (0.30 mL, 2.2 mmol, 1.08 eq.) was added before the mixture was cooled to 0 °C with an ice-water bath. MsCl (0.17 ml, 2.2 mmol, 1.08 eq.) was then added and the mixture was stirred for 20 h, warming slowly to RT. After this time the mixture was washed with sat. $NaHCO_{3(aq)}$ (2 × 30 mL), deionised water (2 × 30 mL) and brine (30 mL). The organic layer was dried ($MgSO_4$) before the solvent was removed *in vacuo* to afford **BnOC6OMs** as a colourless oil (557 mg, 95%) of sufficient purity to use in the following step with no further purification.

[*] Additional coupling is evident, but not fully resolved at this frequency – we believe the signal is a ddd with *J* = 8.9, 1.1, and <1 Hz.

[†] Additional coupling is evident, but not fully resolved at this frequency – we believe the signal is a ddd with *J* = 8.4, 1.4, and <1 Hz, possibly with an additional low *J* coupling.

[‡] The integral of this signal is ca. 10, the remainder is attributed to 'H-grease'

[§] i.e. fragmentation to form a 9-methylanthracene cation

^{1}H NMR (400 MHz, $CDCl_3$) δ 7.37 – 7.31 (m, 4H), 7.31 – 7.26 (m, 1H), 4.50 (s, 2H), 4.22 (t, *J* = 6.5 Hz, 2H), 3.47 (t, *J* = 6.4 Hz, 2H), 2.99 (s, 3H), 1.80 – 1.71 (m, 2H), 1.67 – 1.59 (m, 2H), 1.48 – 1.37 (m, 4H).

^{13}C NMR (101 MHz, $CDCl_3$) δ 138.7, 128.5, 127.8, 127.7, 73.1, 70.3, 70.2, 37.5, 29.7, 29.2, 25.8, 25.4.

MS: ESI^+ m/z: 287.4 $[M+H]^+$

HRMS: m/z: $[M+H]^+$ calculated for $C_{14}H_{23}O_4S$, 287.1317; found, 287.1312

BnOC10OMs

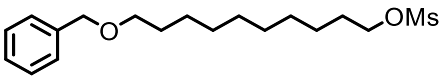

A flask containing **BnOC10OH** (394 mg, 1.49 mmol, 1 eq.) was dried *in vacuo* for ca. 2 h then filled directly with argon. Anhydrous DCM (30 mL) was added, affording a solution to which anhydrous Et_3N (0.22 mL, 1.6 mmol, 1.07 eq.) was added before the mixture was cooled to 0 °C with an ice-water bath. MsCl (0.12 ml, 1.6 mmol, 1.07 eq.) was then added and the mixture was stirred for 20 h, warming slowly to RT. After this time the mixture was washed with sat. $NaHCO_{3(aq)}$ (2 × 30 mL), deionised water (2 × 30 mL) and brine (30 mL). The organic layer was dried ($MgSO_4$) before the solvent was removed *in vacuo* to afford **BnOC10OMs** as a pale yellow oil (489 mg, 96%) of sufficient purity to use in the following step with no further purification.

^{1}H NMR (400 MHz, $CDCl_3$) δ 7.37 – 7.31 (m, 4H), 7.31 – 7.26 (m, 1H), 4.50 (s, 2H), 4.22 (t, *J* = 6.6 Hz, 2H), 3.46 (t, *J* = 6.6 Hz, 2H), 3.00 (s, 3H), 1.78 – 1.70 (m, 2H), 1.65 – 1.56 (m, 2H), 1.44 – 1.26 (m, 12H).

^{13}C NMR (101 MHz, $CDCl_3$) δ 138.8, 128.5, 127.8, 127.6, 73.0, 70.6, 70.3, 37.5, 29.9, 29.57, 29.55, 29.5, 29.3, 29.1, 26.3, 25.5.

MS: ASAP m/z: 343.2 $[M+H]^+$, 342.2 $[M^+]^{+\cdot}$, 341.2 $[M\text{-}H^-]^+$

HRMS: m/z $[M+H]^+$ calculated for $C_{18}H_{31}O_4S$, 343.1943; found, 343.1931

S1.4.4 – Monosubstitution of a dibromide

MeOC8Br

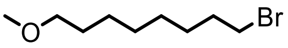

1,8-dibromooctane (2.30 mL, 12.5 mmol, 2.5 eq.) was dissolved in anhydrous THF (30 mL) in a dry flask under argon. Sodium methoxide (0.5 M solution in MeOH, 10.0 mL, 5 mmol, 1 eq.) was added at RT then the reaction was heated at reflux for 2.5 h, over which time a precipitate formed. The reaction was cooled to RT, then quenched with sat. $NH_4Cl_{(aq)}$ (50 mL) and extracted with DCM (3 × 50 mL). The combined organic layers were dried ($MgSO_4$) before the solvent was removed *in vacuo*. The resulting

yellow oil was purified by column chromatography (5 cm Ø, 150 mL SiO_2, eluent: hexane (500 mL*) then 1:1 hexane/DCM) to afford **MeOC8Br** as a colourless oil (826 mg, 74%).

^{1}H NMR (400 MHz, $CDCl_3$) δ 3.40 (t, *J* = 6.9 Hz, 2H), 3.36 (t, *J* = 6.6 Hz, 2H), 3.33 (s, 3H), 1.91 – 1.79 (m, 2H), 1.61 – 1.51 (m, 2H), 1.48 – 1.38 (m, 2H), 1.37 – 1.28 (m, 6H).

^{13}C NMR (101 MHz, $CDCl_3$) δ 73.0, 58.7, 34.2, 32.9, 29.7, 29.4, 28.8, 28.2, 26.2.

MS: ESI$^+$ m/z: 225.1 $[M+H]^+$ (^{81}Br), 223.1 $[M+H]^+$ (^{79}Br)

HRMS: m/z: $[M+H]^+$ calculated for $C_9H_{20}O^{79}Br$, 223.0698; found, 223.0716

S1.4.5 - Thioacetylations

BnOC8SAc

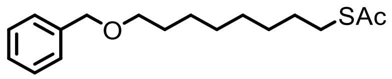

A flask containing **BnOC8OMs** (10.1 g, 32.1 mmol, 1 eq.) was dried *in vacuo* overnight then filled directly with argon. Anhydrous THF (100 mL) was added, affording a solution to which KSAc (4.04 g, 35.4 mmol, 1.1 eq.) was added and the reaction was heated at reflux for 2.5 h, over which time a precipitate formed. After cooling to RT, the reaction was treated with deionised water (100 mL), which dissolved the precipitated salts, then extracted with hexane (2 × 100 mL). The combined organic layers were dried ($MgSO_4$) before the solvent was removed *in vacuo*. The resulting oil was purified by column chromatography (5 cm Ø, 400 mL SiO_2, eluent: 4:1 hexane/EtOAc) to afford **BnOC8SAc** as a yellow oil (7.48 g, 79%).

^{1}H NMR (400 MHz, $CDCl_3$) δ 7.39 – 7.30 (m, 4H), 7.31 – 7.26 (m, 1H), 4.50 (s, 2H), 3.46 (t, *J* = 6.6 Hz, 2H), 2.86 (t, *J* = 7.3 Hz, 2H), 2.32 (s, 3H), 1.66 – 1.50 (m, 4H), 1.41 – 1.24 (m, 8H).

^{13}C NMR (101 MHz, $CDCl_3$) δ 196.2, 138.9, 128.5, 127.8, 127.6, 73.0, 70.6, 30.8, 29.9, 29.6, 29.4, 29.3, 29.2, 28.9, 26.3.

MS: ESI$^+$ m/z: 333.3 $[M+K]^+$, 317.0 $[M+Na]^+$, 312.4 $[M+NH_4]^+$, 295.3 $[M+H]^+$

HRMS: m/z: $[M+H]^+$ calculated for $C_{17}H_{27}O_2S$, 295.1732; found, 295.1740

PyrMOC8SAc

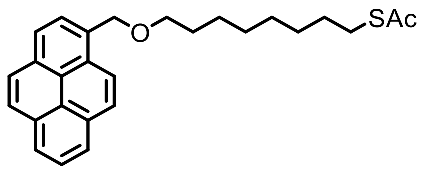

PyrMOC8OMs (475 mg, 1.08 mmol, 1 eq.) was dissolved in anhydrous THF (50 mL) in a dry flask under argon. KSAc (136 mg, 1.19 mmol, 1.1 eq.) was added and the reaction was heated at reflux for 3 h, at which point additional KSAc (25 mg, 0.22 mmol, 0.2 eq.) was added as TLC indicated the reaction was incomplete. The reaction was heated at reflux for a further 17 h, then cooled to RT and treated with

* Allowing recovery of excess, unreacted 1,8-dibromooctane (2.2 g).

deionised water (150 mL). An attempt to extract the mixture with hexane (75 mL) resulted in emulsion formation. Addition of toluene (25 mL) did not resolve the problem, but was possible to wash the mixture using DCM (3 × 100 mL). The combined organic layers were dried ($MgSO_4$) before the solvent was removed *in vacuo*. The resulting yellow solid was purified by column chromatography (3.5 cm Ø, 200 mL SiO_2, eluent: DCM) to afford **PyrMOC8SAc** as a yellow solid (366 mg, 81%).

^{1}H NMR (400 MHz, $CDCl_3$) δ 8.38 (d, *J* = 9.2 Hz, 1H), 8.23 – 8.17 (m[*], 2H), 8.17 – 8.11 (m[†], 2H), 8.06 (s, 2H), 8.05 – 7.98 (m[‡], 2H), 5.22 (s, 2H), 3.60 (t, *J* = 6.6 Hz, 2H), 2.81 (t, *J* = 7.4 Hz, 2H), 2.31 (s, 3H), 1.70 – 1.61 (m, 2H), 1.54 – 1.45 (m, 2H), 1.41 – 1.31 (m, 2H), 1.31 – 1.20 (m, 6H).

^{13}C NMR (101 MHz, $CDCl_3$) δ 196.2, 132.0, 131.4, 131.3, 131.0, 129.5, 127.7, 127.6, 127.5, 127.1, 126.0, 125.31, 125.29, 125.1, 124.9, 124.6, 123.7, 71.7, 70.6, 30.8, 29.9, 29.6, 29.4, 29.3, 29.2, 28.9, 26.3.

MS: ASAP m/z: 418.2 $[M^+]^{+\cdot}$, 417.2 $[M\text{-}H^-]^+$, 215.1 $[C_{17}H_{11}]^+$[§]

HRMS: m/z $[M^+]^{+\cdot}$ calculated for $C_{27}H_{30}O_2S$, 418.1967; found, 418.1964

m.p.: 69 – 70.5 °C

2-AMOC8SAc

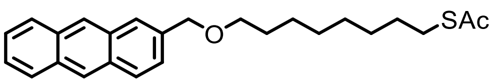

2-AMOC8OMs (283 mg, 0.683 mmol, 1 eq.) was dissolved in anhydrous THF (25 mL) in a dry flask under argon. KSAc (101 mg, 0.884 mmol, 1.3 eq.) was added and the reaction was heated at reflux for 18 h. After cooling to RT, the reaction was treated with deionised water (50 mL) then extracted with DCM (3 × 25 mL), adding brine (25 mL) to aid layer separation. The combined organic layers were dried ($MgSO_4$) before the solvent was removed *in vacuo*. The resulting yellow-brown solid was purified by column chromatography (3.5 cm Ø, 150 mL SiO_2, eluent: DCM) to afford **2-AMOC8SAc** as a yellow solid (187 mg, 69%).

^{1}H NMR (400 MHz, $CDCl_3$) δ 8.41 (d, *J* = 3.0 Hz, 2H), 8.04 – 7.96 (m, 3H), 7.92 (s, 1H), 7.50 – 7.42 (m, 3H), 4.69 (s, 2H), 3.54 (t, *J* = 6.6 Hz, 2H), 2.85 (t, *J* = 7.4 Hz, 2H), 2.31 (s, 3H), 1.70 – 1.61 (m, 2H), 1.61 – 1.49 (m, 2H[**]), 1.44 – 1.27 (m, 8H).

^{13}C NMR (101 MHz, $CDCl_3$) δ 196.3, 135.8, 132.0, 131.8, 131.6, 131.4, 128.6, 128.32, 128.27, 126.28, 126.27, 126.2, 125.7, 125.5, 125.4, 73.2, 70.7, 30.8, 29.9, 29.6, 29.4, 29.3, 29.2, 28.9, 26.3.

MS: ASAP m/z: 394.2 $[M^+]^{+\cdot}$, 393.2 $[M\text{-}H^-]^+$, 191.1 $[C_{15}H_{11}]^+$[††]

HRMS: m/z $[M\text{-}H^-]^+$ calculated for $C_{25}H_{29}O_2S$, 393.1888; found, 393.1884

m.p.: 89 – 91.5 °C

[*] This signal appears to be two overlapping triplets each with *J* = ca. 7.7 Hz
[†] This signal appears to be two overlapping doublets with *J* = ca. 7.7 Hz and *J* = ca. 9.2 Hz
[‡] This signal appears to be an overlapping doublet (*J* = ca. 7.8 Hz) and triplet (*J* = ca. 7.6 Hz)
[§] i.e. fragmentation to form a 1-methylpyrene cation
[**] This peak is overlapped by a residual water peak.
[††] i.e. fragmentation to form a 2-methylanthracene cation

9-AMOC8 SAc

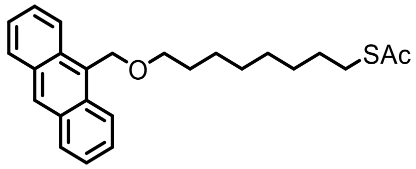

A flask containing **9-AMOC8OMs** (200 mg, 0.482 mmol, 1 eq.) was dried *in vacuo* for 3 h then filled directly with argon. Anhydrous THF (20 mL) was added, affording a solution to which KSAc (72 mg, 0.63 mmol, 1.3 eq.) was added and the reaction was heated at reflux for 18 h. After cooling to RT, the reaction was treated with deionised water (50 mL) then extracted with DCM (3 × 25 mL), adding brine (25 mL) to aid layer separation. The combined organic layers were dried ($MgSO_4$) before the solvent was removed *in vacuo*. The resulting yellow-brown oil was purified by column chromatography (3.5 cm Ø, 150 mL SiO_2, eluent: DCM), which afforded the desired product contaminated with some minor aliphatic impurities based on the ^1H NMR spectrum (128 mg)[*]. Further column chromatography (4 cm Ø, 100 mL SiO_2, gradient elution from 4:1 DCM/hexane to 16:1 DCM/hexane) afforded little improvement in purity. By treating the resulting oil with MeOH (ca. 5 mL), sonicating the mixture, and storing overnight at 3 °C, a small amount of precipitate was obtained. This was removed by filtration and the filtrate was dried *in vacuo* to afford product of improved purity[†]. No additional benefit was gained by repeating this process. Finally, after investigating alternative eluents, further column chromatography (3 cm Ø, 100 mL SiO_2, gradient elution from 1:1 toluene/hexane to toluene) afforded **9-AMOC8SAc** of acceptable purity[‡] as a yellow oil (75 mg, 39%).

^1H NMR (400 MHz, $CDCl_3$) δ 8.46 (s, 1H), 8.39 (d[§], *J* = 8.9 Hz, 2H), 8.01 (d[**], *J* = 8.4 Hz, 2H), 7.54 (ddd, *J* = 8.9, 6.5, 1.4 Hz, 2H), 7.47 (ddd, *J* = 8.4, 6.5, 1.1 Hz, 2H), 5.46 (s, 2H), 3.66 (t, *J* = 6.6 Hz, 2H), 2.84 (t, *J* = 7.3 Hz, 2H), 2.32 (s, 3H), 1.67 – 1.59 (m, 2H), 1.56 – 1.47 (m, 2H[††]), 1.37 – 1.20 (m, 8H[‡‡]).

^{13}C NMR (101 MHz, $CDCl_3$) δ 196.2, 131.6, 131.1, 129.2, 129.1, 128.4, 126.2, 125.1, 124.6, 70.8, 65.1, 30.8, 30.0, 29.6, 29.34, 29.27, 29.2, 28.9, 26.3.

MS: ASAP m/z: 394.2 $[M^+]^{+\cdot}$, 393.2 $[M\text{-}H^-]^+$, 191.1 $[C_{15}H_{11}]^+$[§§]

HRMS: m/z $[M\text{-}H^-]^+$ calculated for $C_{25}H_{29}O_2S$, 393.1888; found, 393.1860

[*] Estimated purity based on ^1H NMR integrals was 90-95%.

[†] Estimated purity based on ^1H NMR integrals was ca. 97%.

[‡] One minor additional aliphatic peak remains (see spectrum below), but we estimate the purity of the compound at >98% based on ^1H NMR integrals. We note that the aromatic region is well-resolved, showing no impurity peaks, and conclude that this minor, inseparable impurity is unlikely to impact on our conductance studies.

[§] Additional coupling is evident, but not fully resolved at this frequency – we believe the signal is a ddd with *J* = 8.9, 1.1, and <1 Hz.

[**] Additional coupling is evident, but not fully resolved at this frequency – we believe the signal is a ddd with *J* = 8.4, 1.4, and <1 Hz, possibly with an additional low *J* coupling.

[††] This signal overlaps a small residual water peak.

[‡‡] The integral of this signal is ca. 10, the remainder is attributed to 'H-grease'.

[§§] i.e. fragmentation to form a 9-methylanthracene cation.

MeOC8SAc

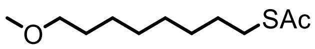

MeOC8Br (650 mg, 2.91 mmol, 1 eq.) was dissolved in anhydrous THF (50 mL) in a dry flask under argon. KSAc (432 mg, 3.78 mmol, 1.3 eq.) was added and the reaction was heated at reflux for 4 h, over which time the reaction became cloudy and turned from colourless to pale yellow., then cooled to RT and treated with deionised water (150 mL). After cooling to RT, the reaction was treated with deionised water (100 mL), which dissolved the precipitated salts, then extracted with hexane (3 × 50 mL). The combined organic layers were dried (MgSO4) before the solvent was removed *in vacuo*. The resulting yellow oil was purified by column chromatography (3.5 cm Ø, 150 mL SiO_2, gradient elution from 1:1 DCM/hexane to DCM) to afford **MeOC8SAc** as a yellow oil (496 mg, 78%).

^{1}H NMR (400 MHz, $CDCl_3$) δ 3.36 (t, *J* = 6.6 Hz, 2H), 3.32 (s, 3H), 2.86 (t, *J* = 7.3 Hz, 2H), 2.32 (s, 3H), 1.61 – 1.50 (m, 4H), 1.40 – 1.24 (m, 8H).

^{13}C NMR (101 MHz, $CDCl_3$) δ 196.2, 73.0, 58.7, 30.8, 29.8, 29.6, 29.4, 29.3, 29.2, 28.9, 26.2.

MS: ESI$^+$ m/z: 241.2 $[M+Na]^+$, 219.7 $[M+H]^+$

HRMS: m/z: $[M+H]^+$ calculated for $C_{11}H_{23}O_2S$, 219.1419; found, 219.1434

BnOC6SAc

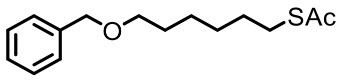

A flask containing **BnOC6OMs** (499 mg, 1.74 mmol, 1 eq.) was dried *in vacuo* for 3 h then filled directly with argon. Anhydrous THF (50 mL) was added, affording a solution to which KSAc (259 mg, 2.27 mmol, 1.3 eq.) was added and the reaction was heated at reflux for 17 h. After cooling to RT, the reaction was treated with deionised water (100 mL), which dissolved the precipitate that had formed, then extracted with hexane (2 × 100 mL). The combined organic layers were washed with brine (100 mL) then dried ($MgSO_4$) before the solvent was removed *in vacuo*. The resulting yellow oil was purified by column chromatography (3.5 cm Ø, 75 mL SiO_2, gradient elution from 6:1 hexane/EtOAc to 4:1 hexane/EtOAc) to afford **BnOC6SAc** as a yellow oil (303 mg, 65%).

^{1}H NMR (400 MHz, $CDCl_3$) δ 7.37 – 7.31 (m, 4H), 7.30 – 7.26 (m, 1H), 4.50 (s, 2H), 3.46 (t, *J* = 6.5 Hz, 2H), 2.86 (t, *J* = 7.4 Hz, 2H), 2.32 (s, 3H), 1.66 – 1.53 (m, 4H*), 1.44 – 1.33 (m, 4H).

^{13}C NMR (101 MHz, $CDCl_3$) δ 196.2, 138.8, 128.5, 127.8, 127.6, 73.0, 70.4, 30.8, 29.7, 29.6, 29.2, 28.8, 25.9.

MS: ASAP m/z: 267.1 $[M+H]^+$, 265.1 $[M-H^-]^+$

HRMS: m/z: $[M+H]^+$ calculated for $C_{15}H_{23}O_2S$, 267.1419; found, 267.1407

* This signal overlaps the residual water peak

BnOC10SAc

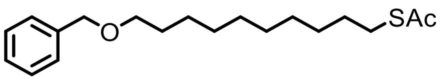

A flask containing **BnOC10OMs** (435 mg, 1.27 mmol, 1 eq.) was dried *in vacuo* for 3 h then filled directly with argon. Anhydrous THF (50 mL) was added, affording a solution to which KSAc (189 mg, 1.65 mmol, 1.3 eq.) was added and the reaction was heated at reflux for 17 h. After cooling to RT, the reaction was treated with deionised water (100 mL), which dissolved the precipitate that had formed, then extracted with hexane (2 × 100 mL). The combined organic layers were washed with brine (100 mL) then dried ($MgSO_4$) before the solvent was removed *in vacuo*. The resulting pale yellow oil was purified by column chromatography (3.5 cm Ø, 75 mL SiO_2, gradient elution from 6:1 hexane/EtOAc to 4:1 hexane/EtOAc) to afford **BnOC10SAc** as a pale yellow oil (238 mg, 58%).

^{1}H NMR (400 MHz, $CDCl_3$) δ 7.37 – 7.31 (m, 4H), 7.31 – 7.26 (m, 1H), 4.50 (s, 2H), 3.46 (t, *J* = 6.6 Hz, 2H), 2.86 (t, *J* = 7.4 Hz, 2H), 2.32 (s, 3H), 1.65 – 1.51 (m, 4H*), 1.40 – 1.25 (m, 12H†).

^{13}C NMR (101 MHz, $CDCl_3$) δ 196.0, 138.6, 128.2, 127.5, 127.3, 72.7, 70.4, 30.5, 29.6, 29.4‡, 29.32, 29.28, 29.04, 28.97, 28.7, 26.1.

MS: ASAP m/z: 323.2 $[M+H]^+$

HRMS: m/z: $[M+H]^+$ calculated for $C_{19}H_{31}O_2S$, 323.2045; found, 323.2051

---

* This signal overlaps the residual water peak

† The integral of this signal is closer to 13, the remainder is attributed to 'H-grease'

‡ This signal is believed to be two overlapping peaks

S1.5 ^{1}H NMR Spectra of Key Species

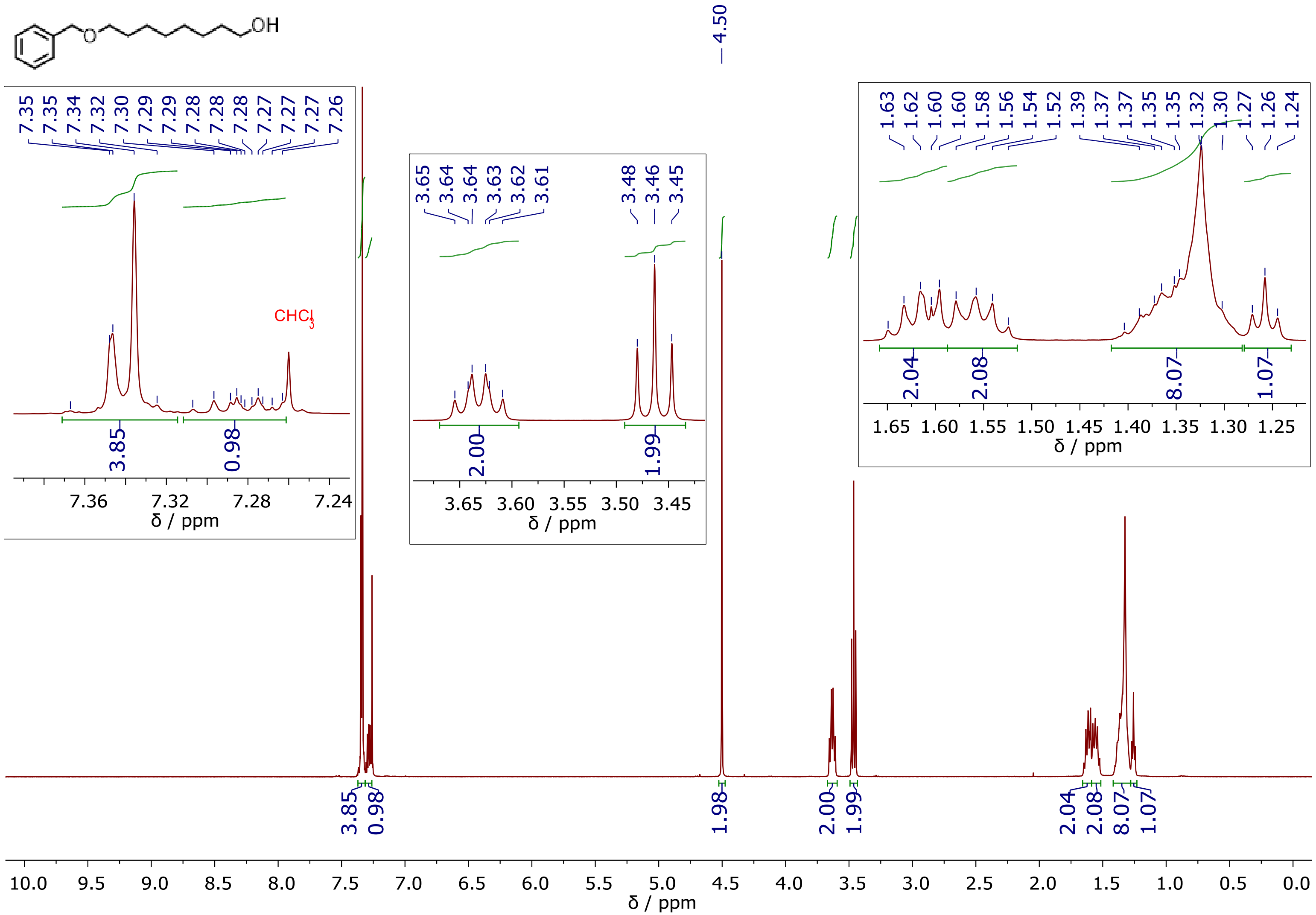

Figure S1: 400 MHz ^{1}H NMR spectrum of **BnOC8OH** in $CDCl_3$.

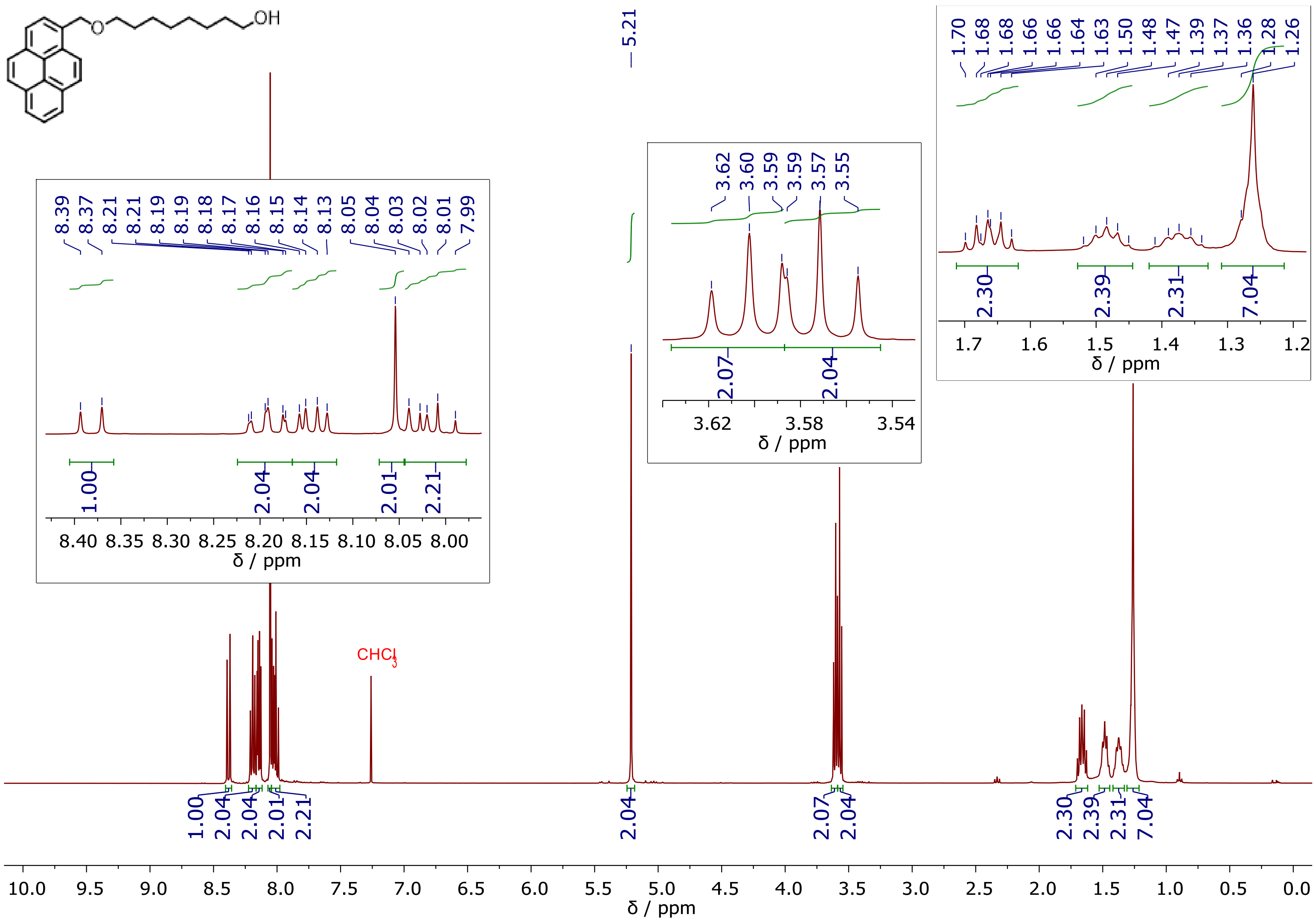

Figure S2: 400 MHz ^{1}H NMR spectrum of **PyrMOC8OH** in $CDCl_3$.

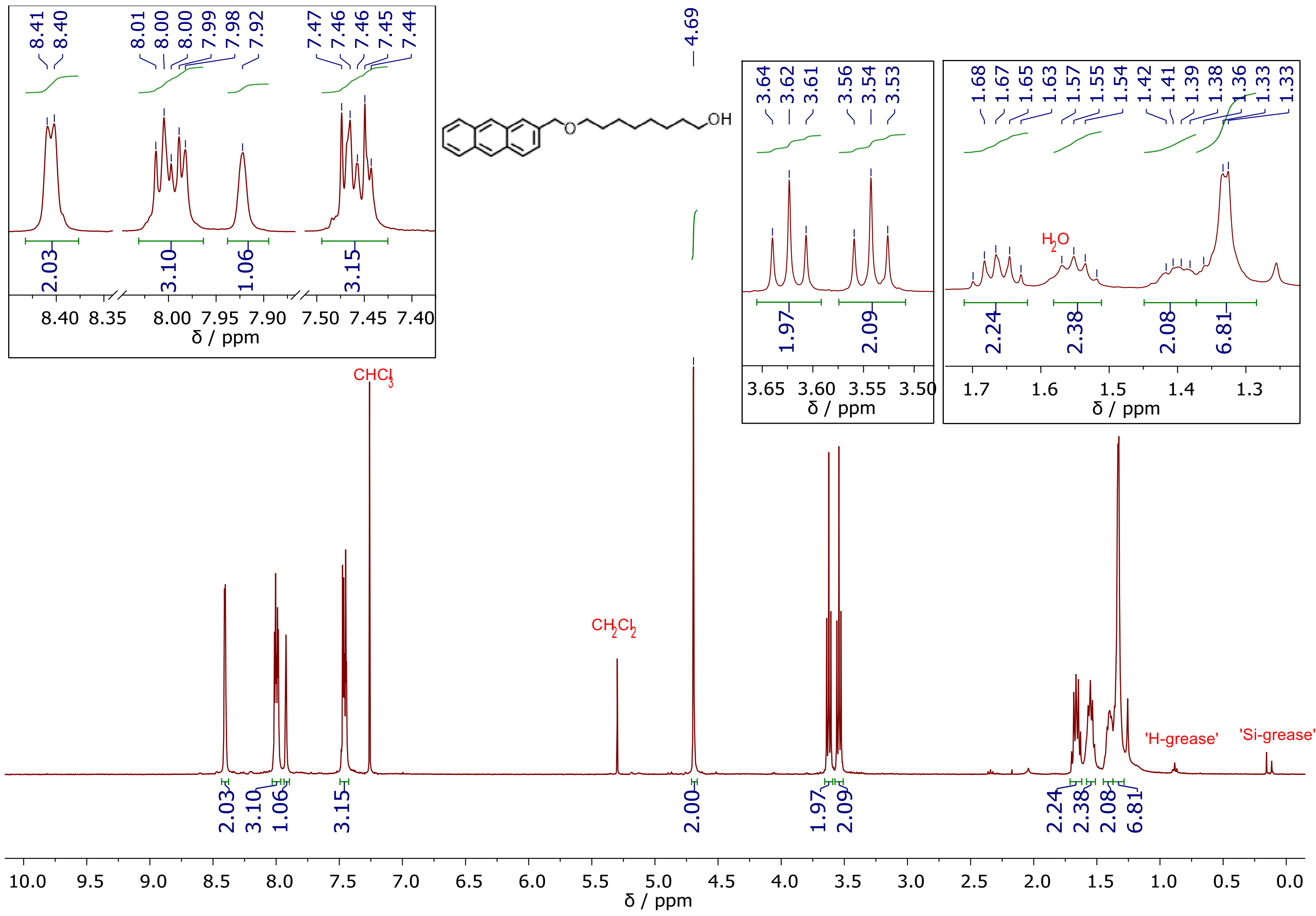

Figure S3: 400 MHz ^{1}H NMR spectrum of **2-AMOC8OH** in $CDCl_3$.

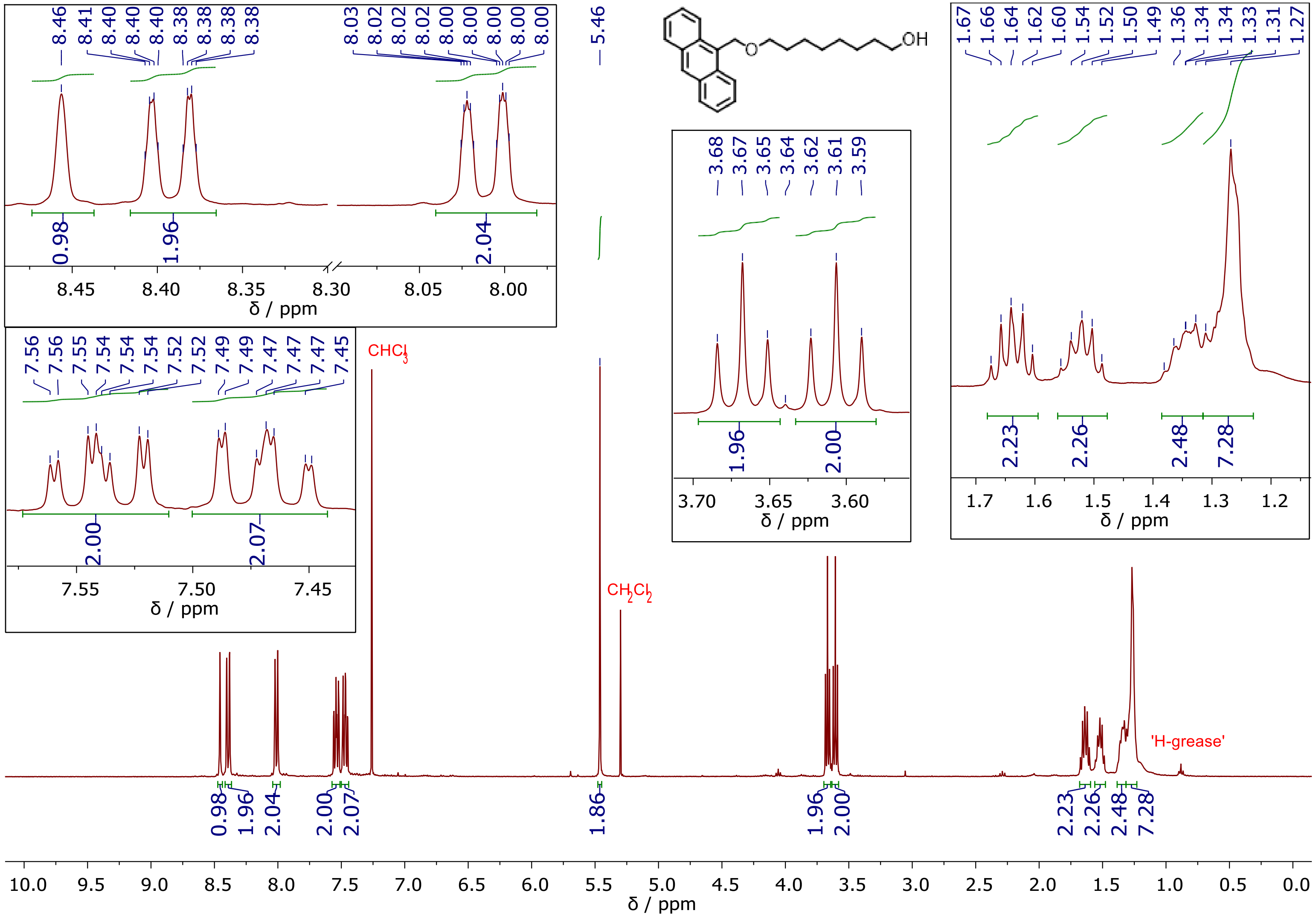

Figure S4: 400 MHz ^{1}H NMR spectrum of **9-AMOC8OH** in $CDCl_3$.

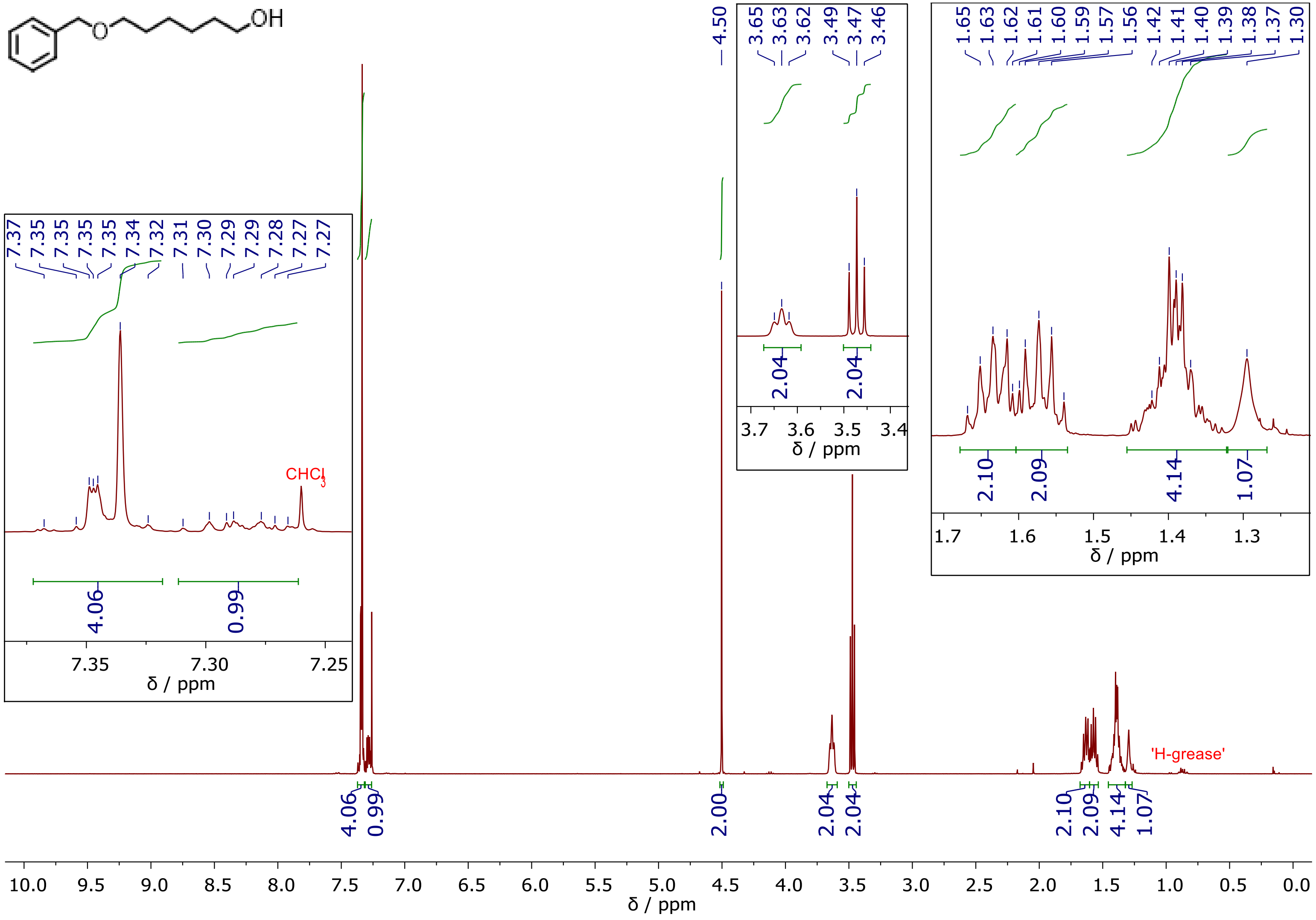

Figure S5: 400 MHz ^{1}H NMR spectrum of **BnOC6OH** in $CDCl_3$.

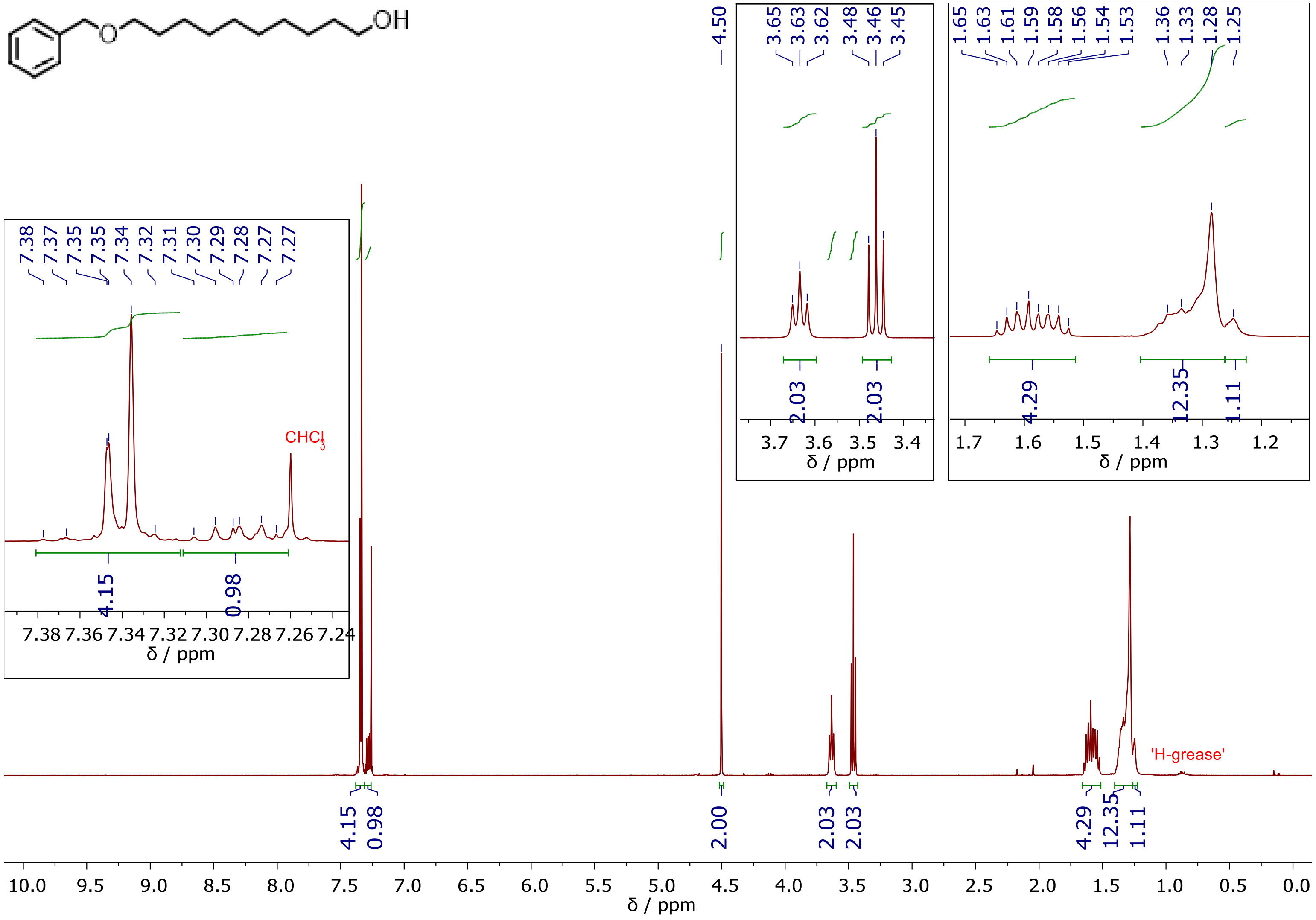

Figure S6: 400 MHz ^{1}H NMR spectrum of **BnOC10OH** in $CDCl_3$.

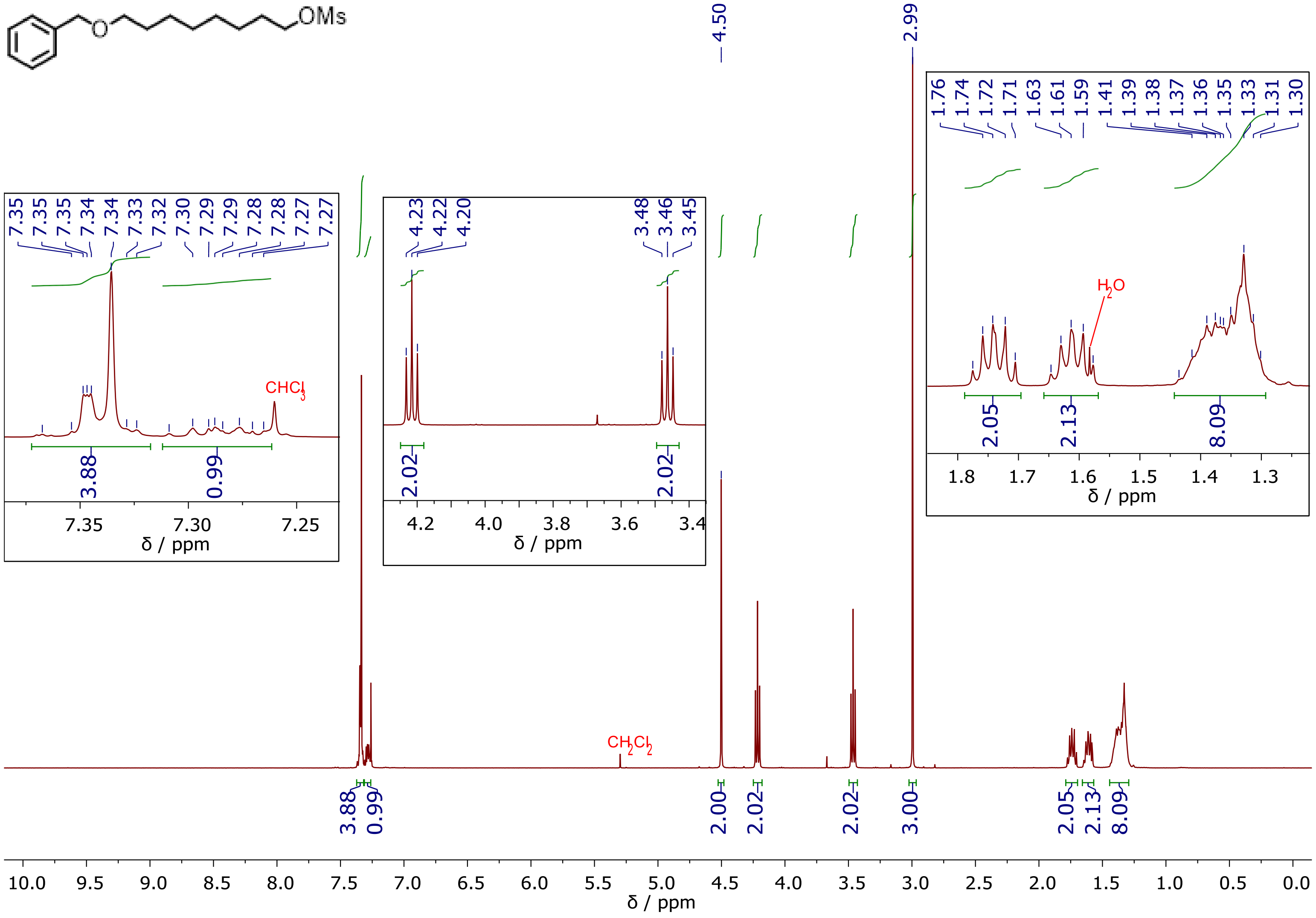

Figure S7: 400 MHz ^{1}H NMR spectrum of **BnOC8OMs** in $CDCl_3$.

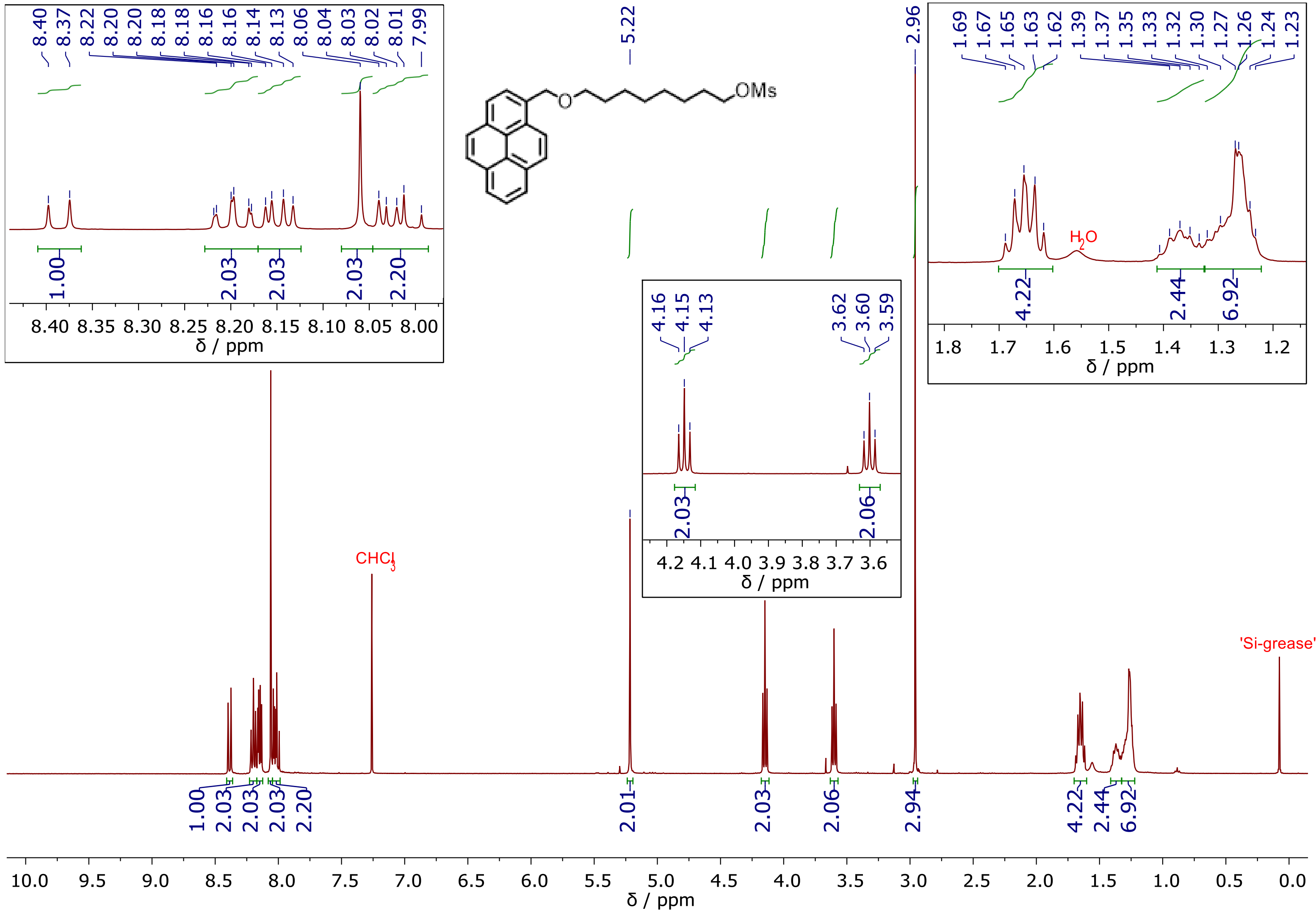

Figure S8: 400 MHz ^{1}H NMR spectrum of **PyrMOC8OMs** in $CDCl_3$.

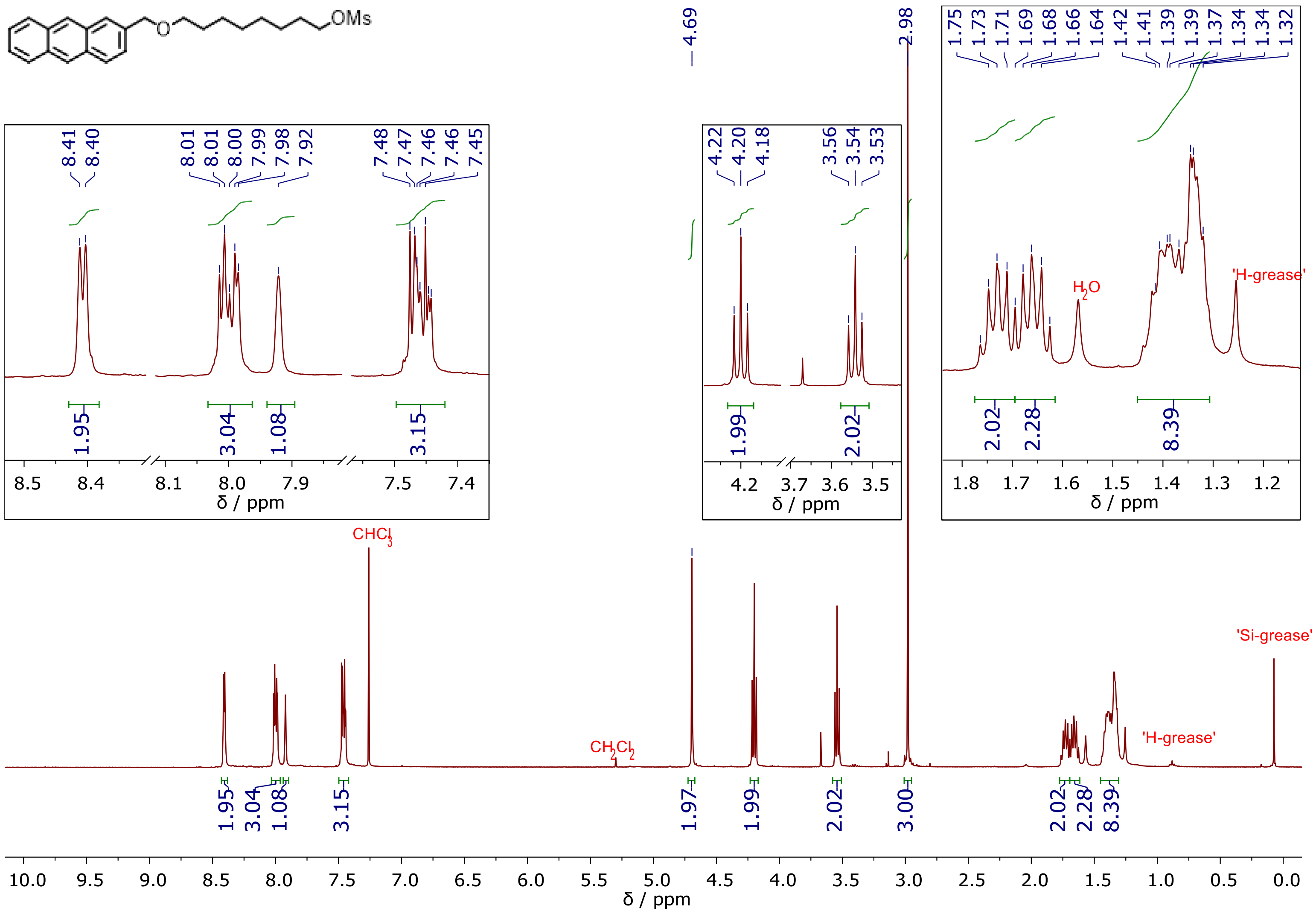

Figure S9: 400 MHz ^{1}H NMR spectrum of **2-AMOC8OMs** in $CDCl_3$.

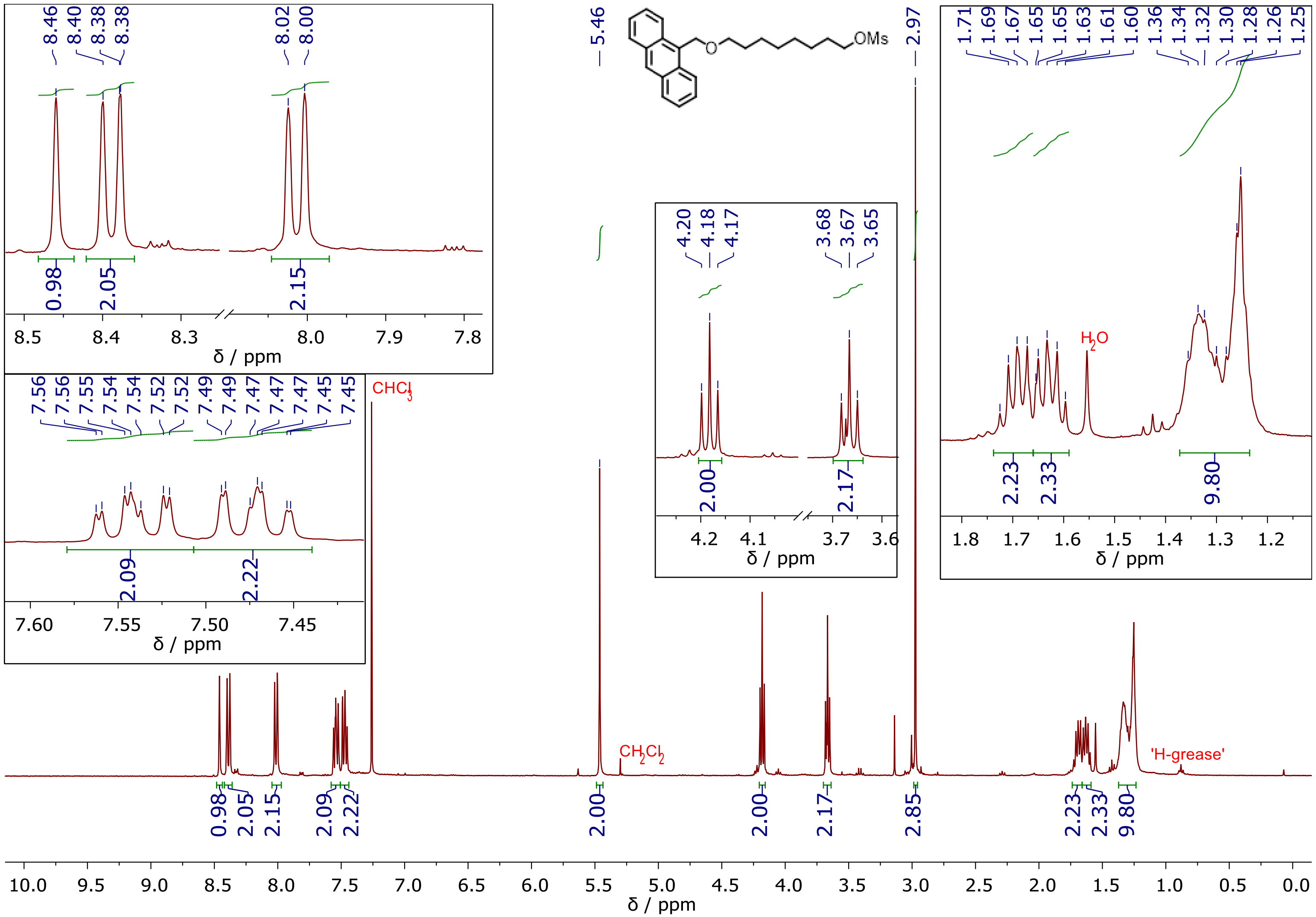

Figure S10: 400 MHz ^{1}H NMR spectrum of **9-AMOC8OMs** in $CDCl_3$.

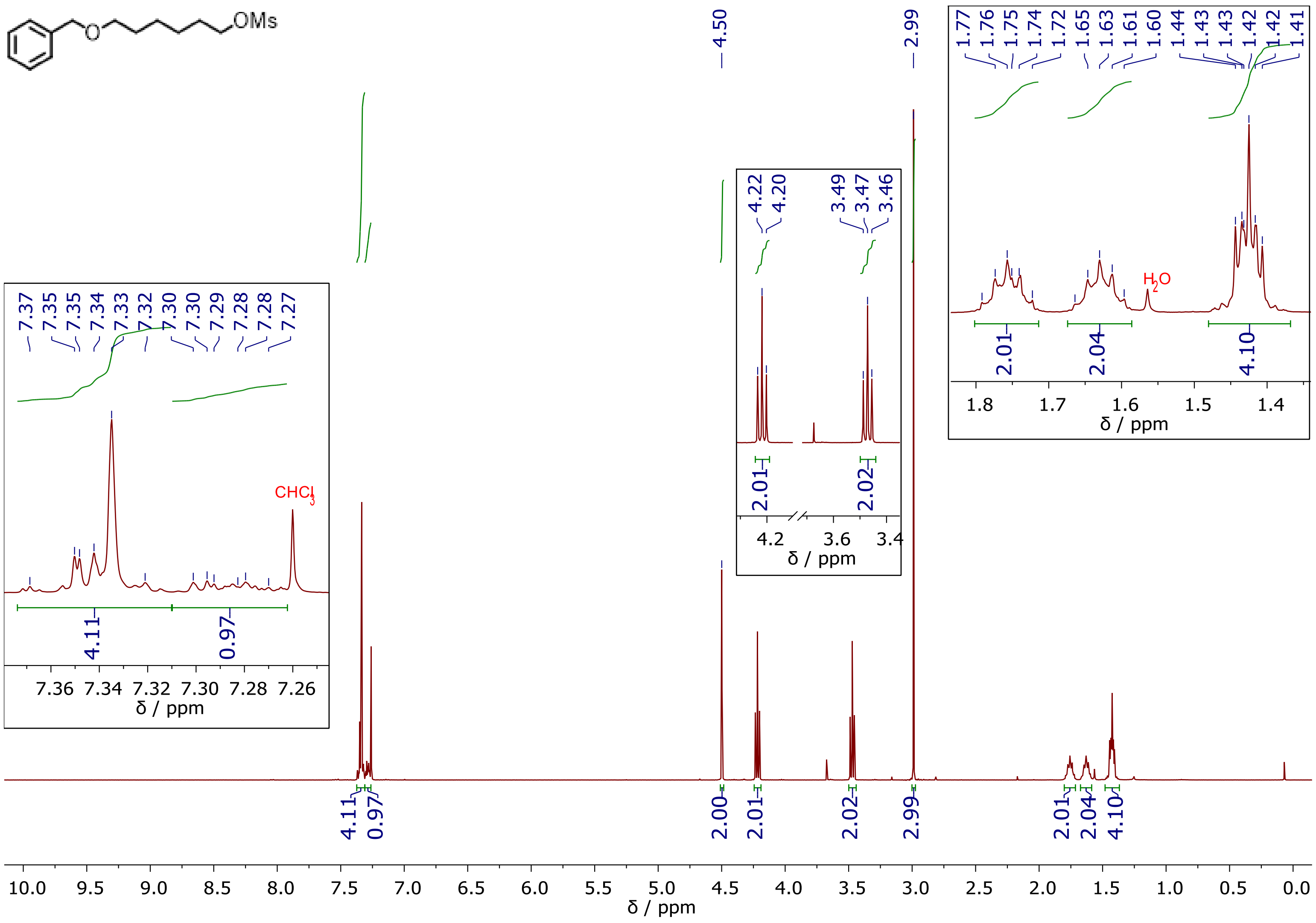

Figure S11: 400 MHz ^{1}H NMR spectrum of **BnOC6OMs** in $CDCl_3$.

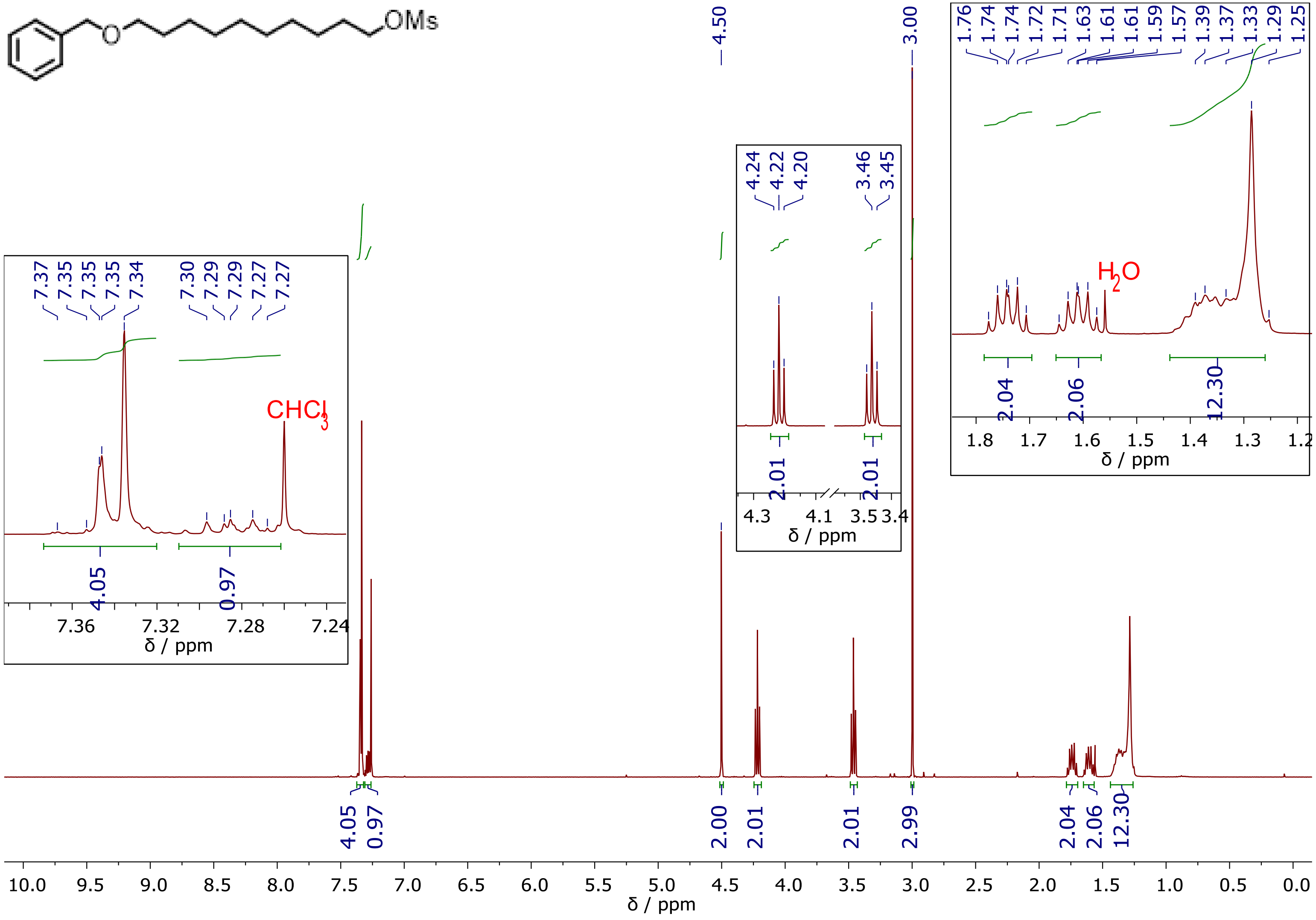

Figure S12: 400 MHz ^{1}H NMR spectrum of **BnOC10OMs** in $CDCl_3$.

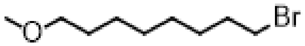

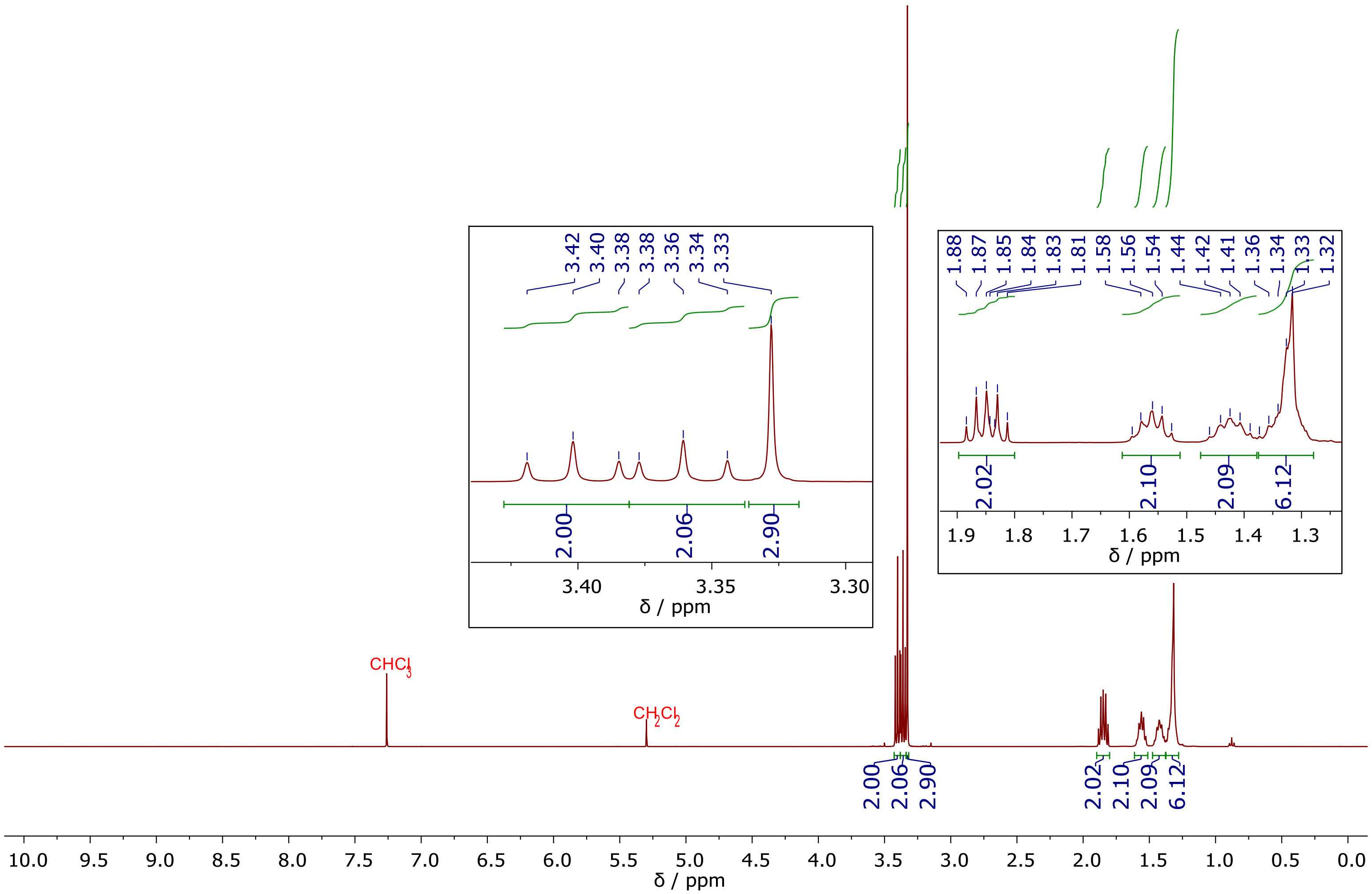

Figure S13: 400 MHz ^{1}H NMR spectrum of **MeOC8Br** in $CDCl_3$.

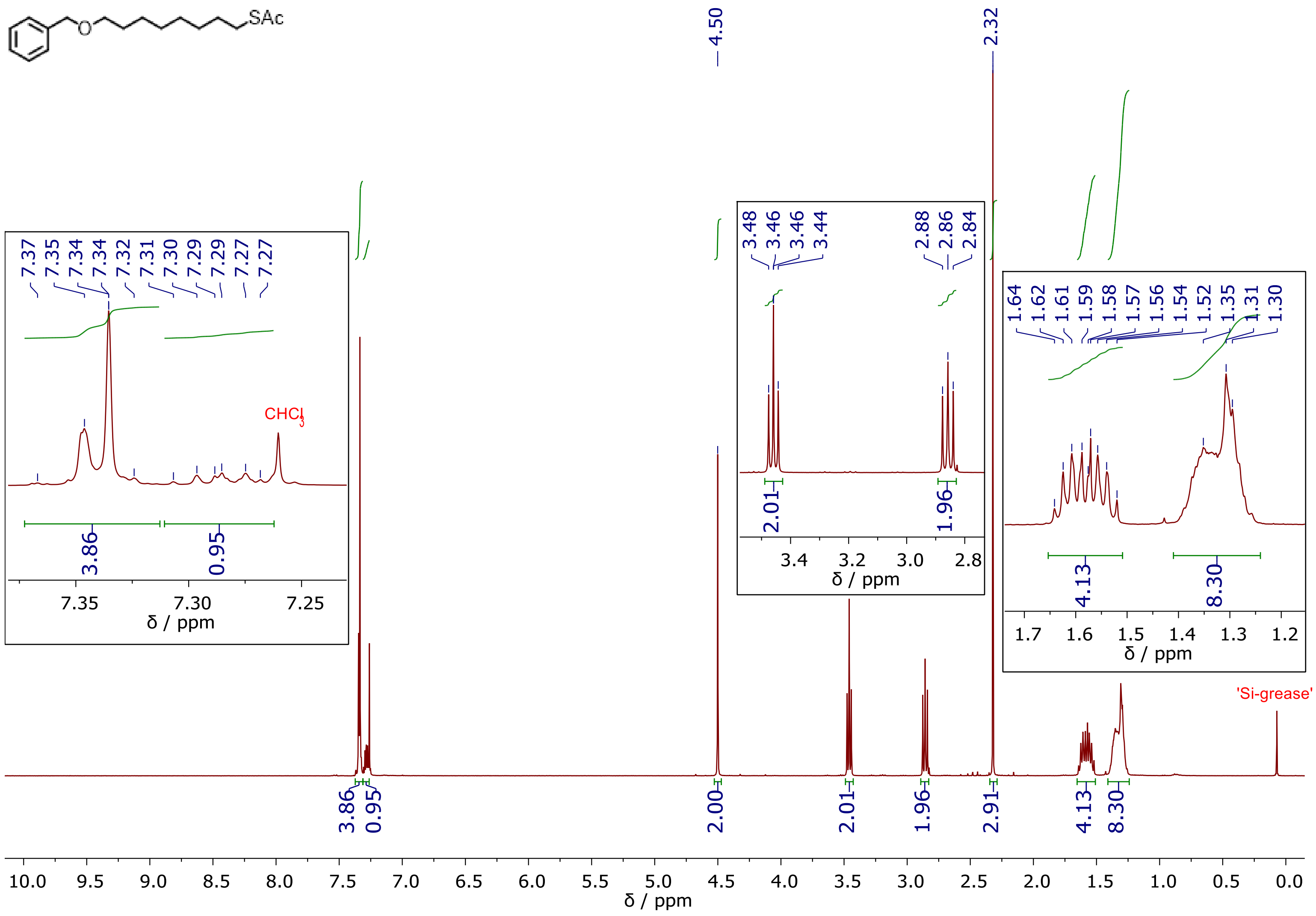

Figure S14: 400 MHz ^{1}H NMR spectrum of **BnOC8SAc** in $CDCl_3$.

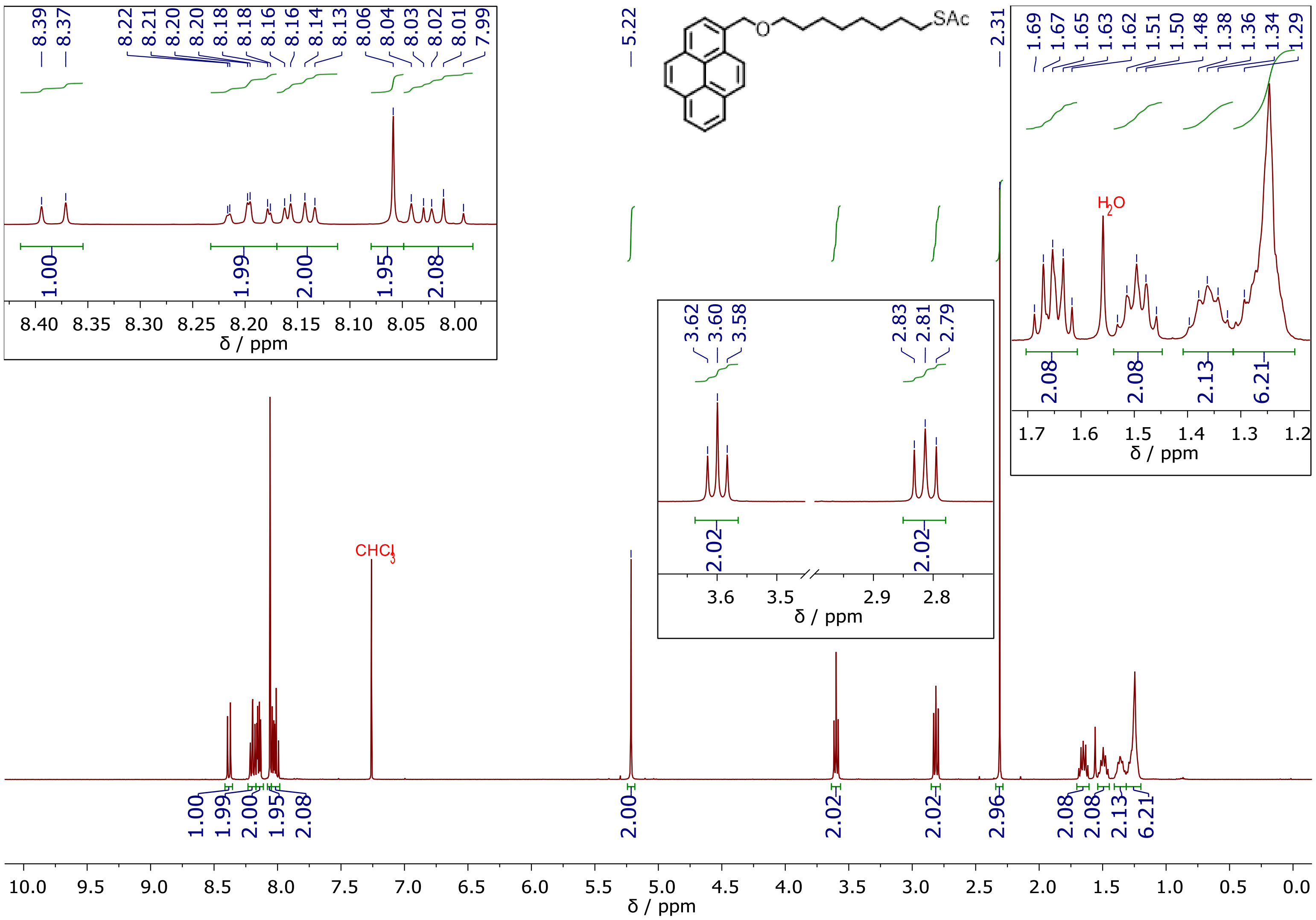

Figure S15: 400 MHz ^{1}H NMR spectrum of **PyrMOC8SAc** in $CDCl_3$.

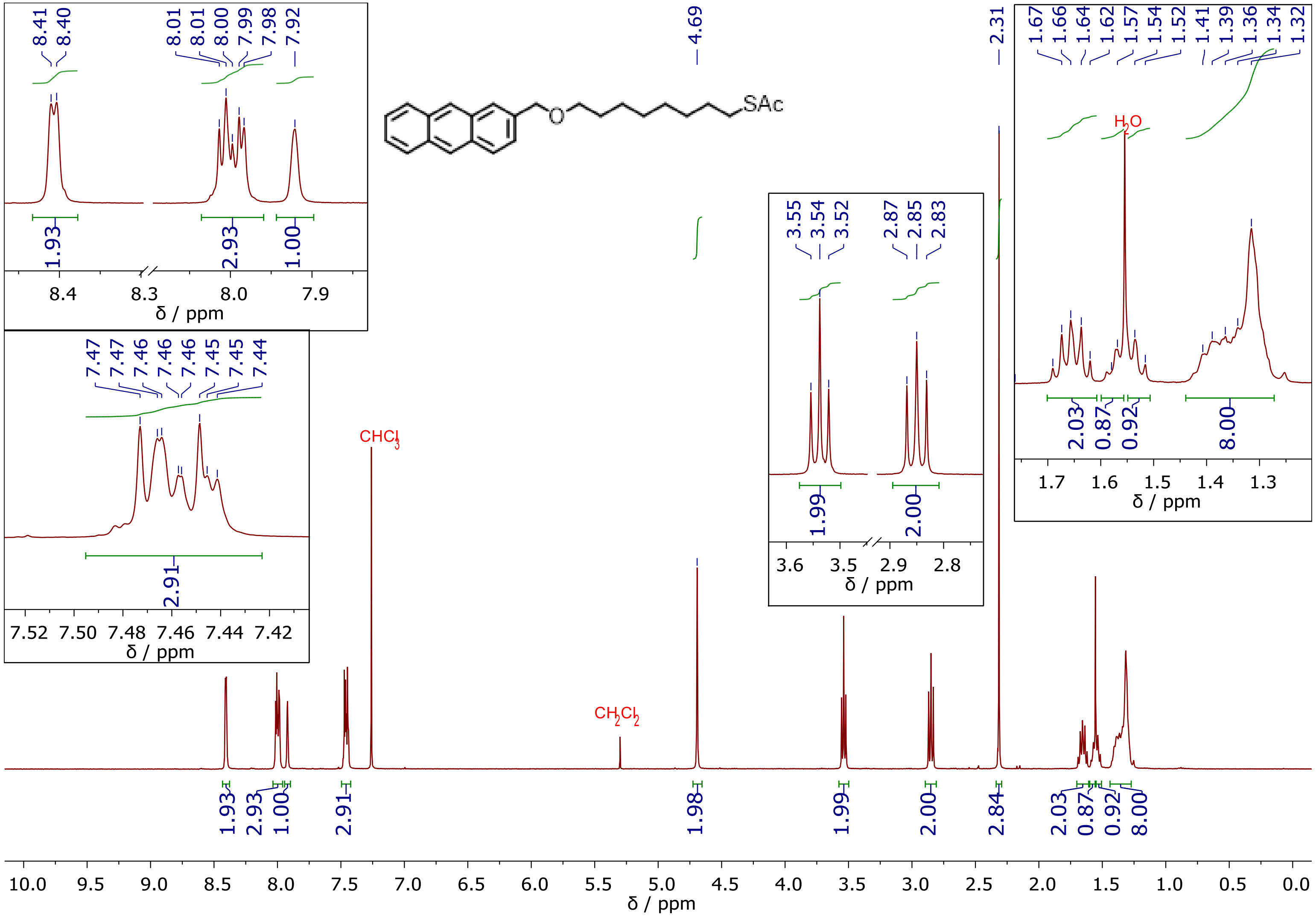

Figure S16: 400 MHz ^{1}H NMR spectrum of **2-AMOC8SAc** in $CDCl_3$.

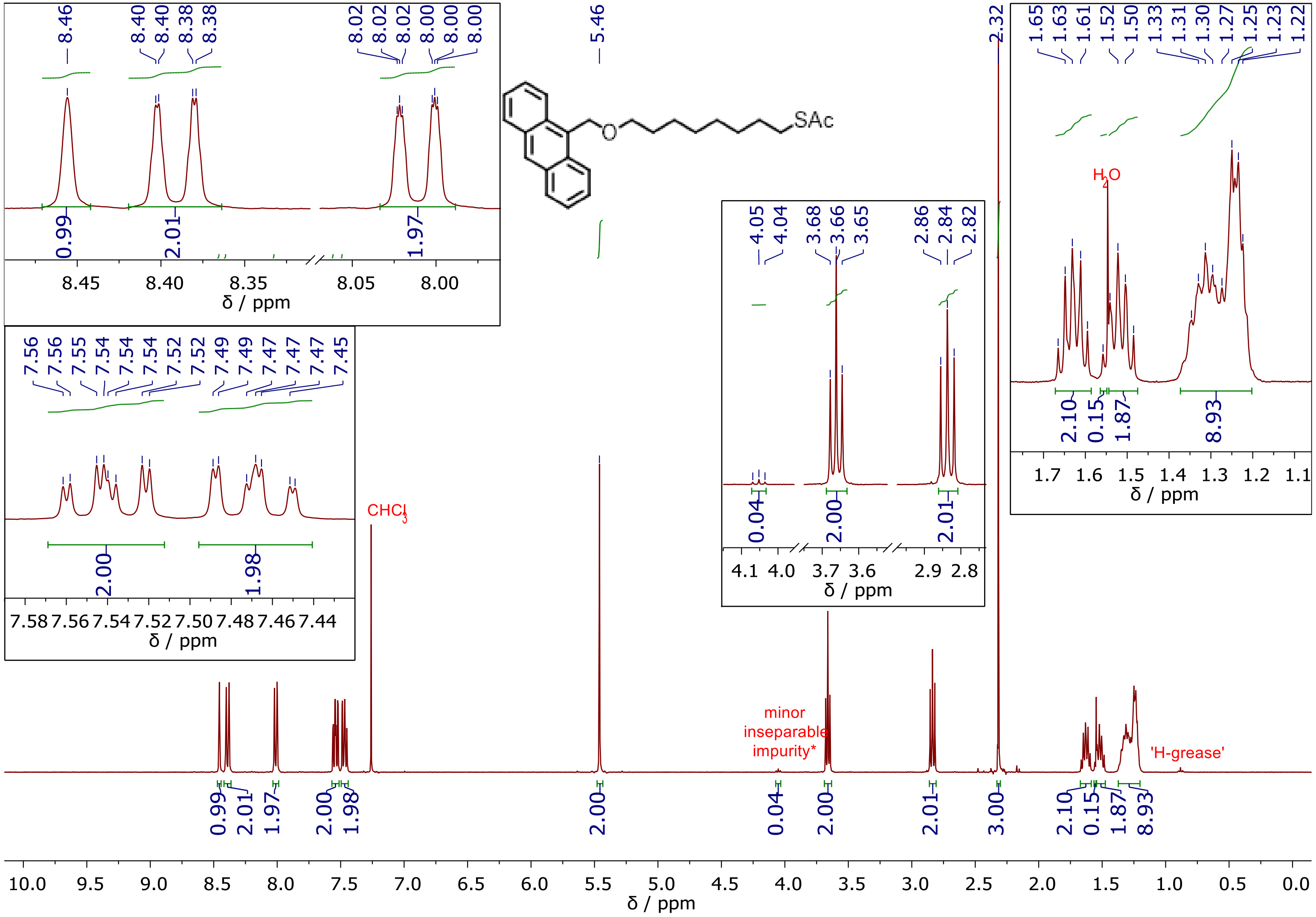

Figure S17: 400 MHz ^{1}H NMR spectrum of **9-AMOC8SAc** in $CDCl_3$.

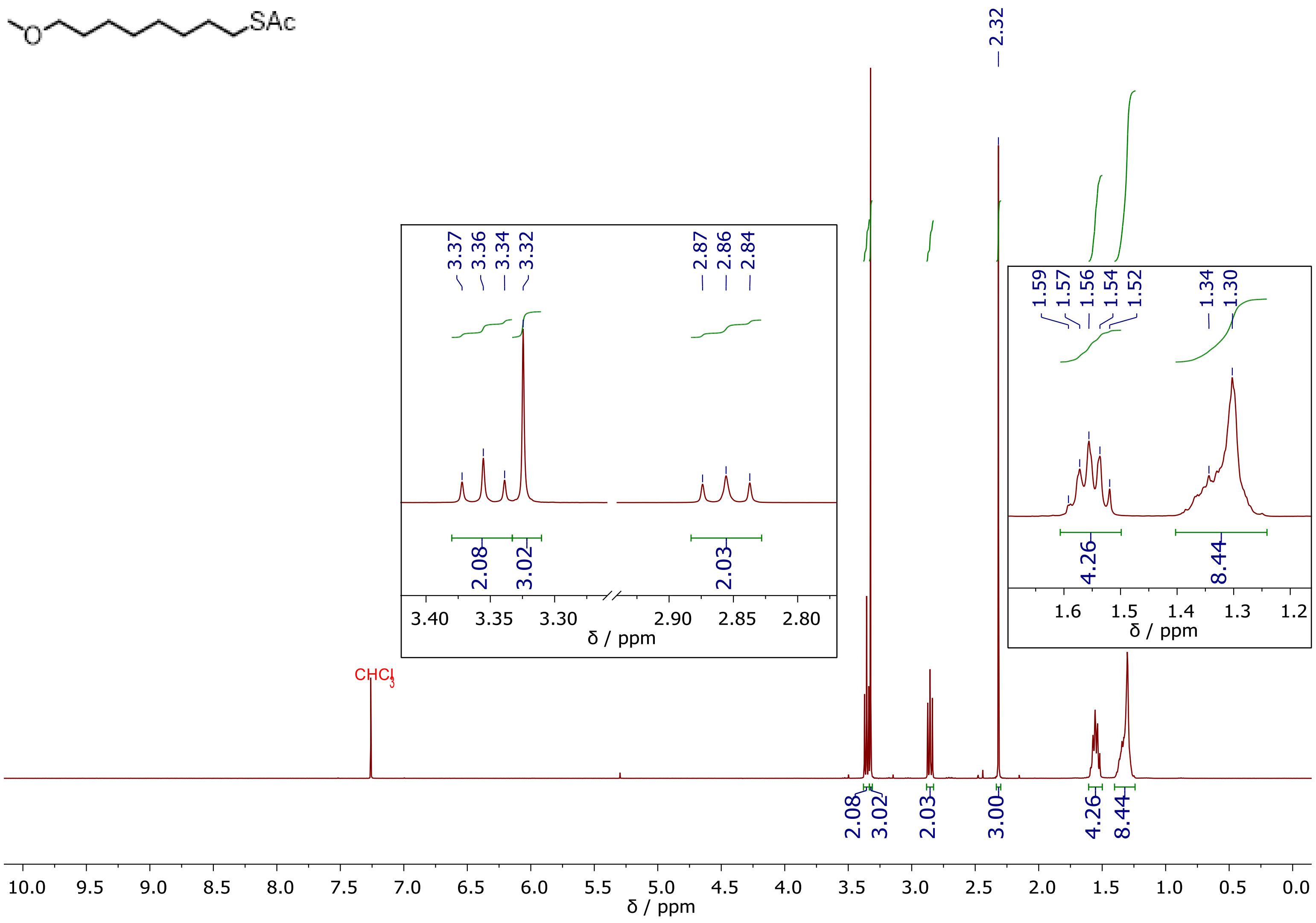

Figure S18: 400 MHz ^{1}H NMR spectrum of **MeOC8SAc** in $CDCl_3$.

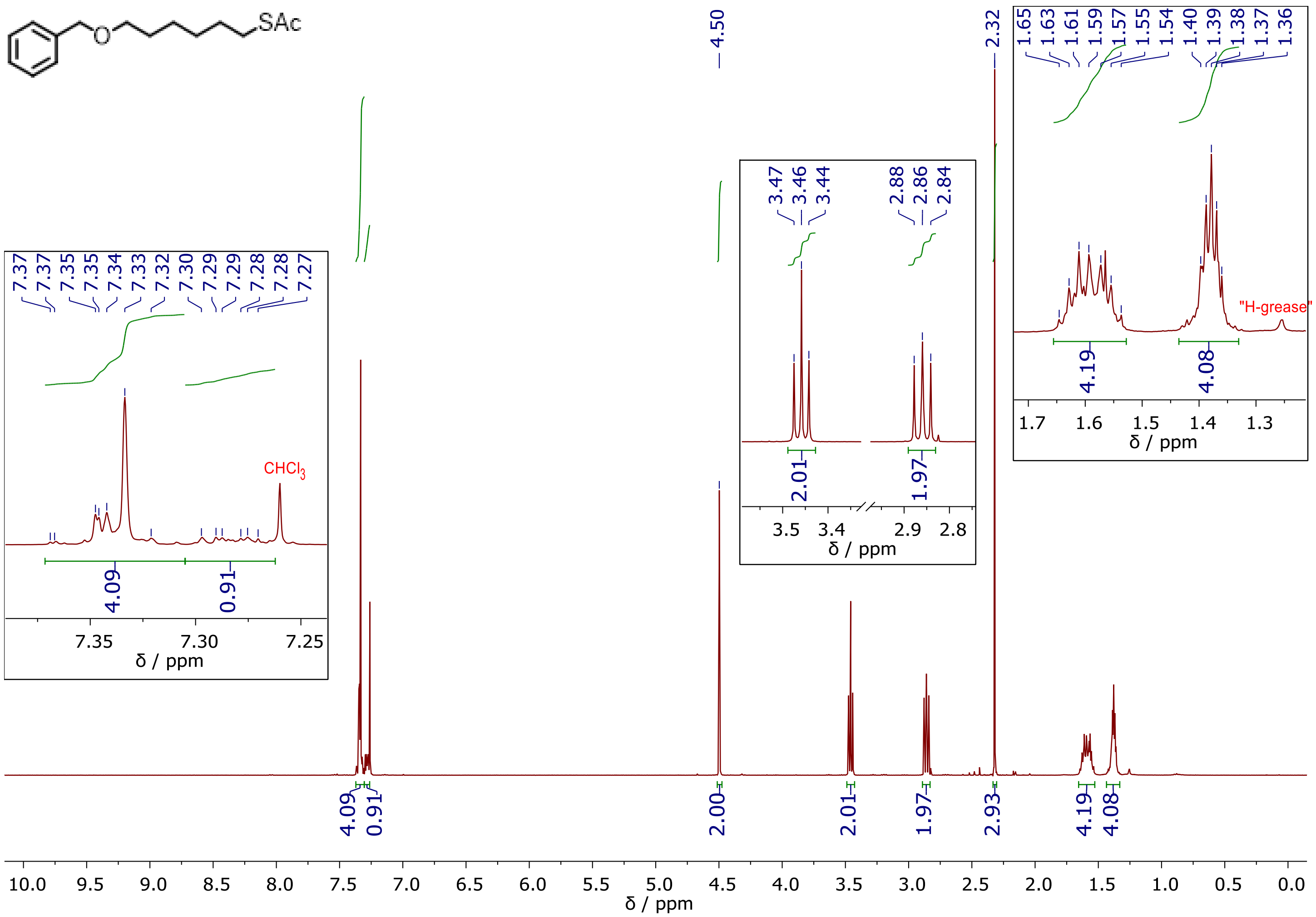

Figure S19: 400 MHz ^{1}H NMR spectrum of **BnOC6SAc** in $CDCl_3$.

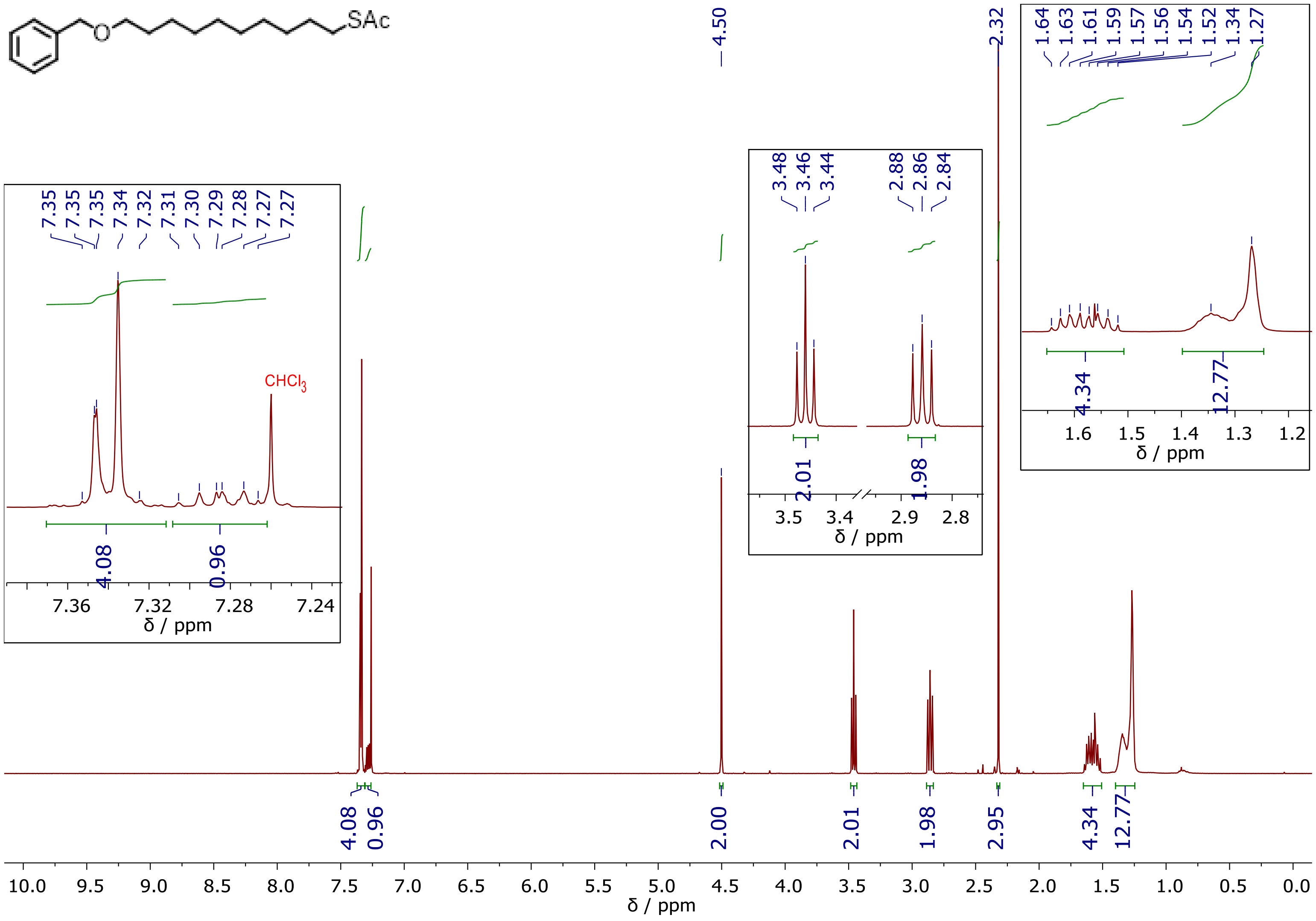

Figure S20: 400 MHz ^{1}H NMR spectrum of **BnOC10SAc** in $CDCl_3$.

1.6 ^{13}C NMR Spectra of Key Species

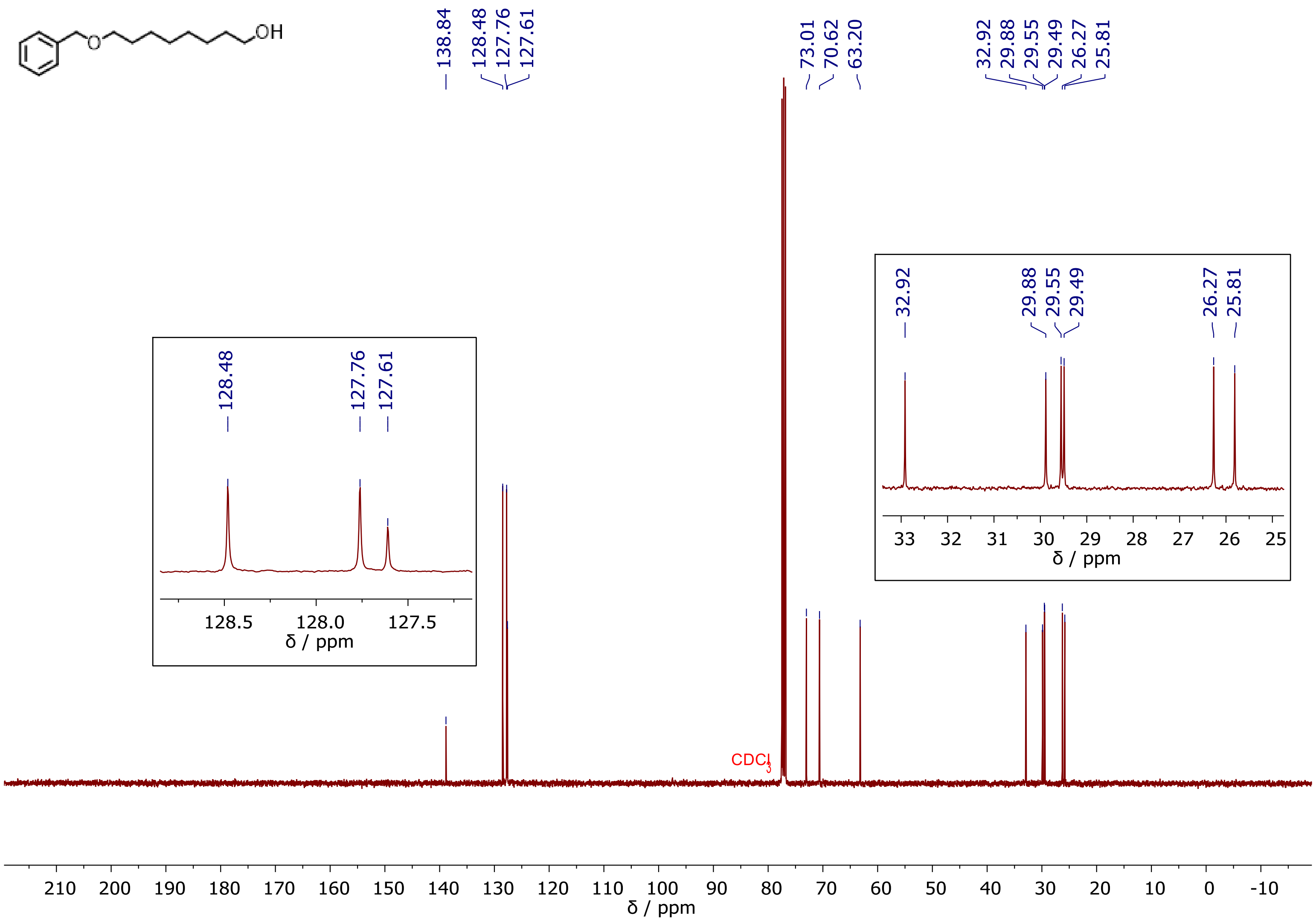

Figure S21: 101 MHz ^{13}C NMR spectrum of **BnOC8OH** in $CDCl_3$.

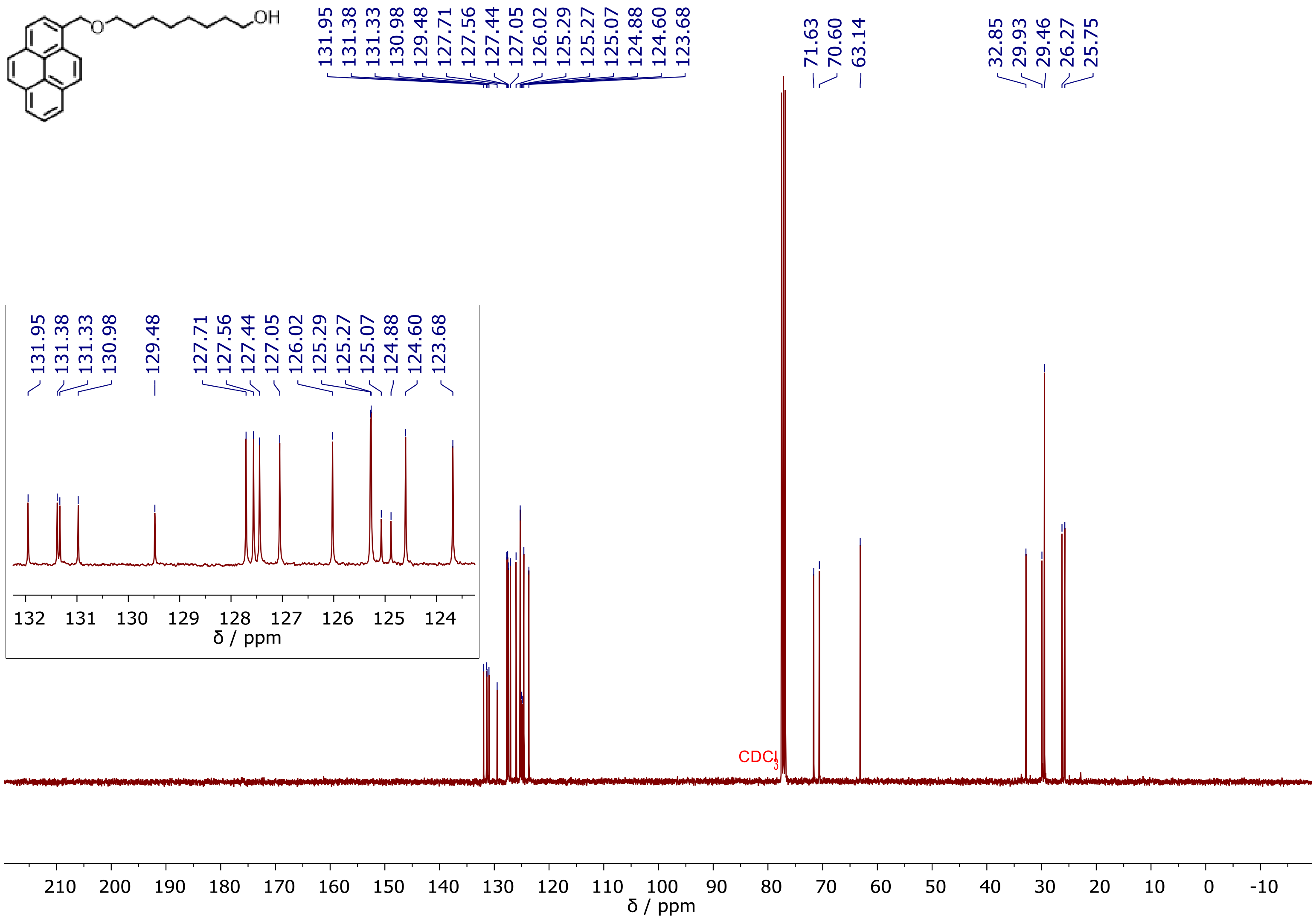

Figure S22: 101 MHz ^{13}C NMR spectrum of **PyrMOC8OH** in $CDCl_3$.

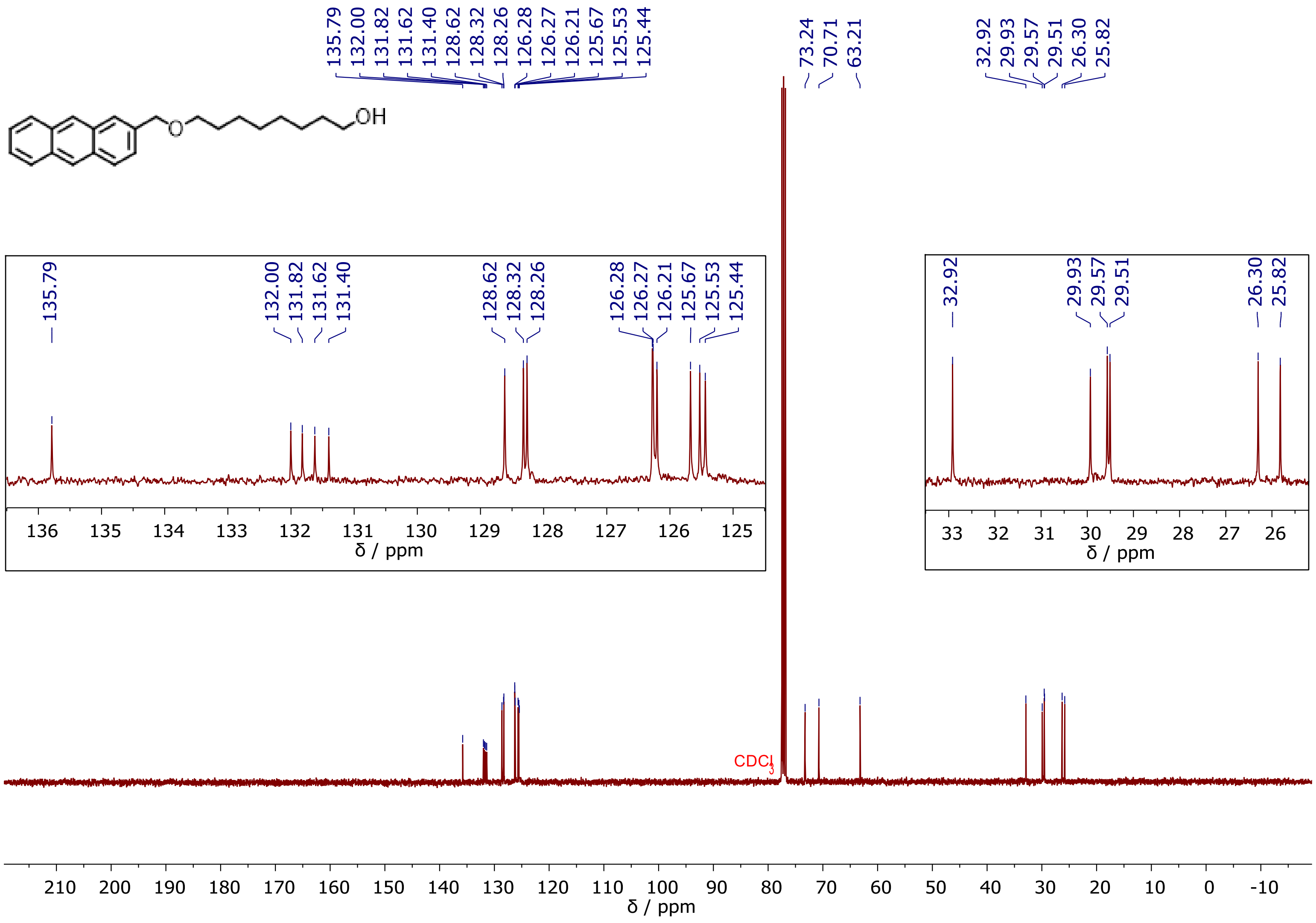

Figure S23: 101 MHz ^{13}C NMR spectrum of **2-AMOC8OH** in $CDCl_3$.

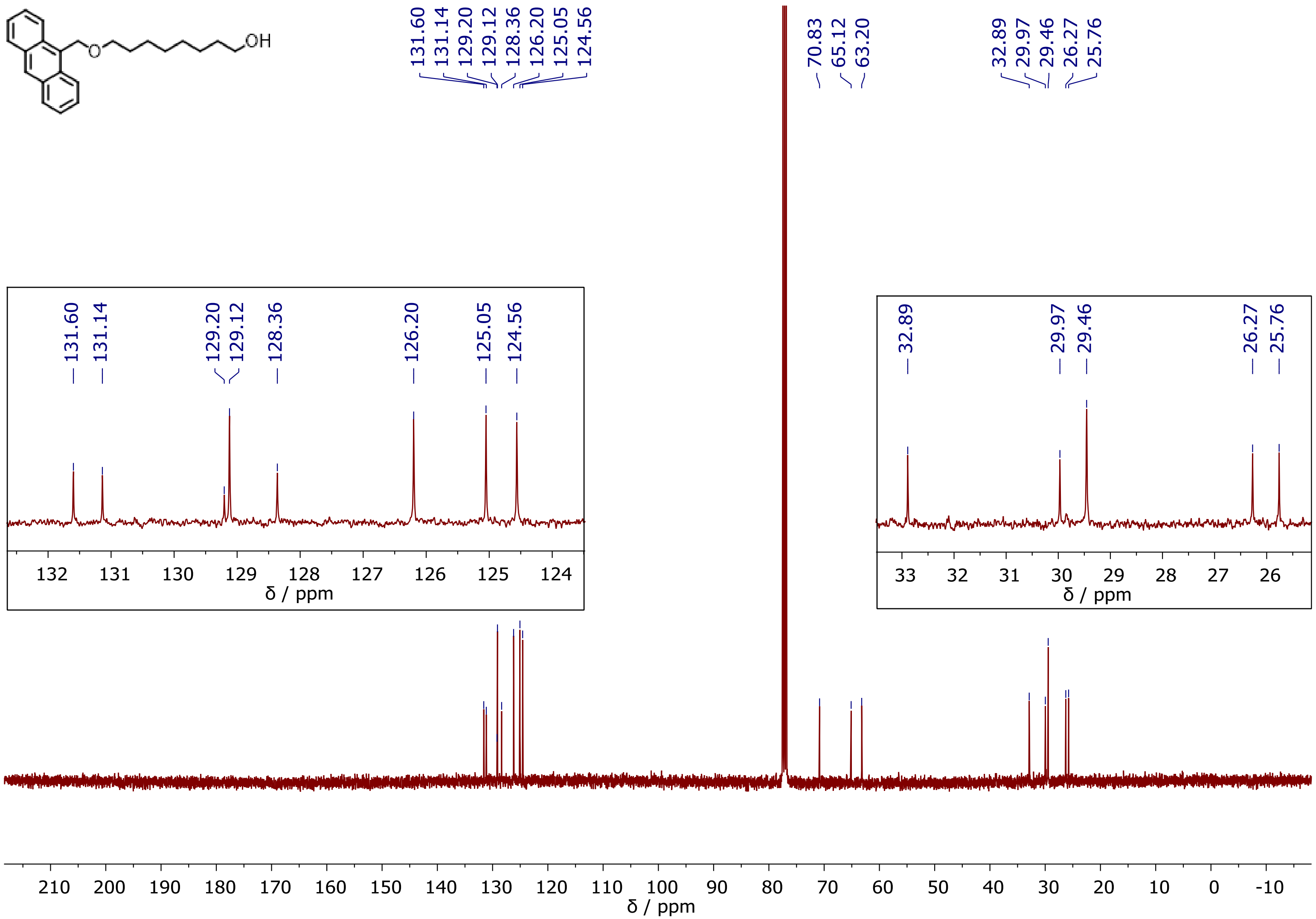

Figure S24: 101 MHz ^{13}C NMR spectrum of **9-AMOC8OH** in $CDCl_3$.

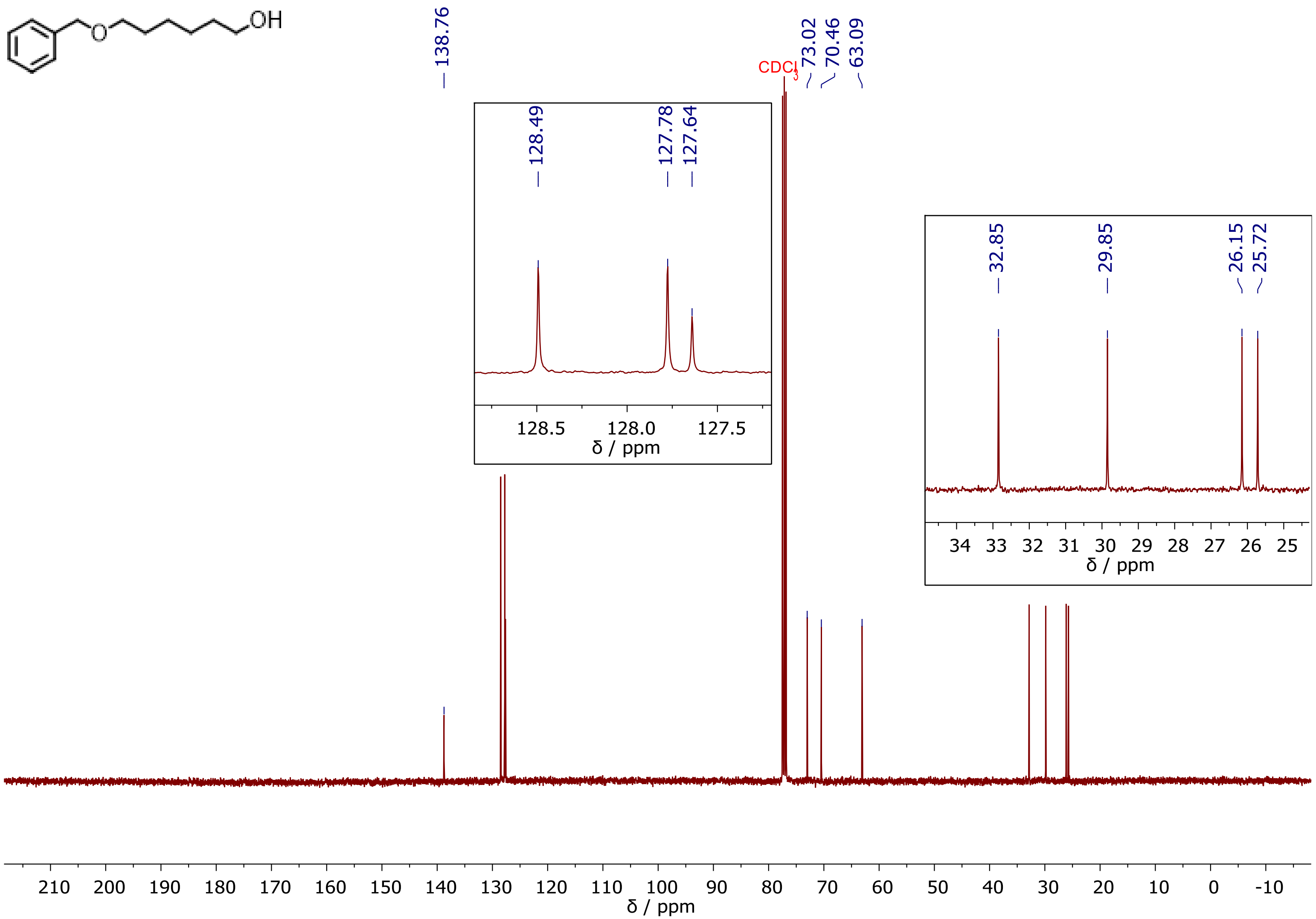

Figure S25: 101 MHz ^{13}C NMR spectrum of **BnOC6OH** in $CDCl_3$.

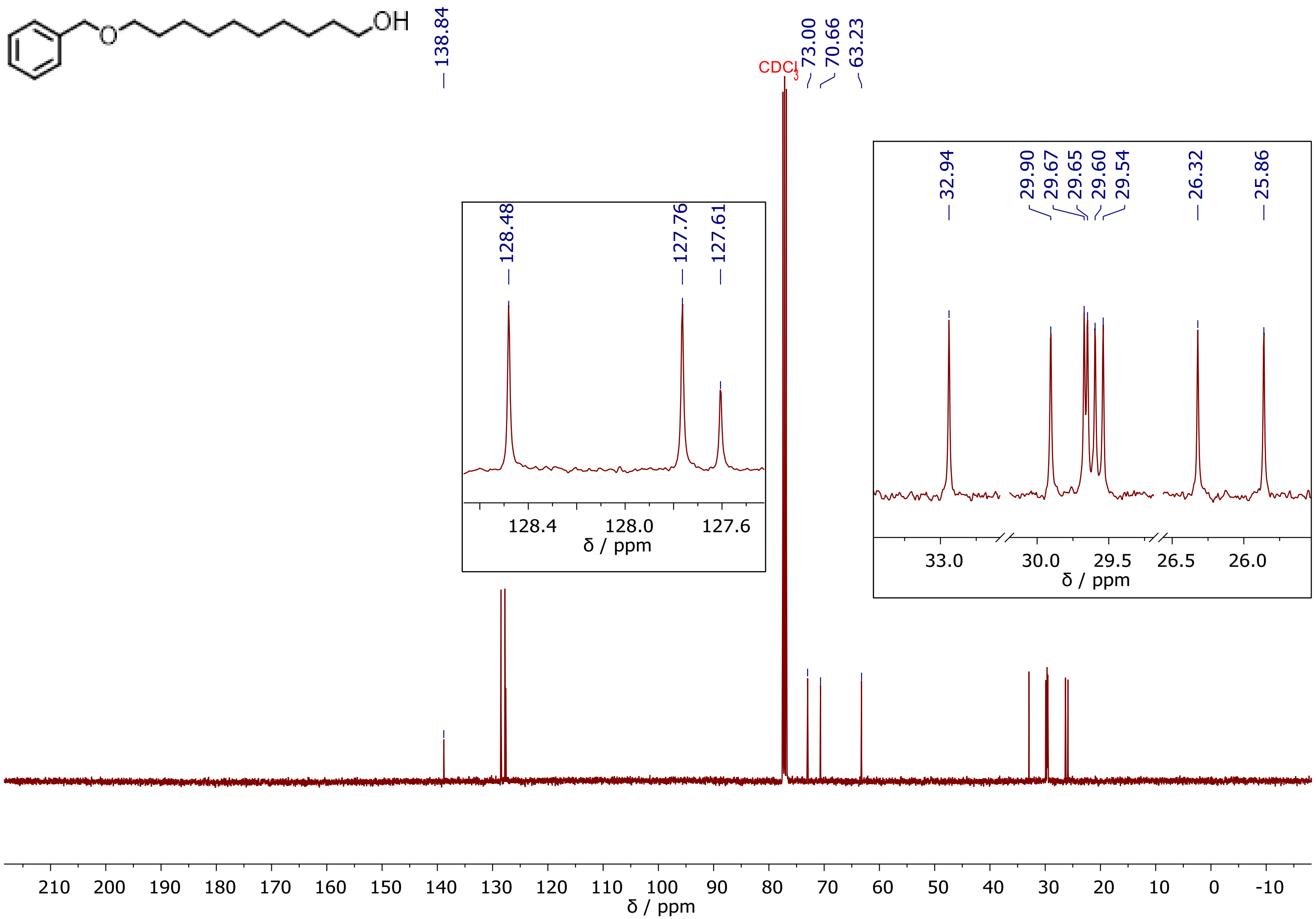

Figure S26: 101 MHz ^{13}C NMR spectrum of **BnOC10OH** in $CDCl_3$.

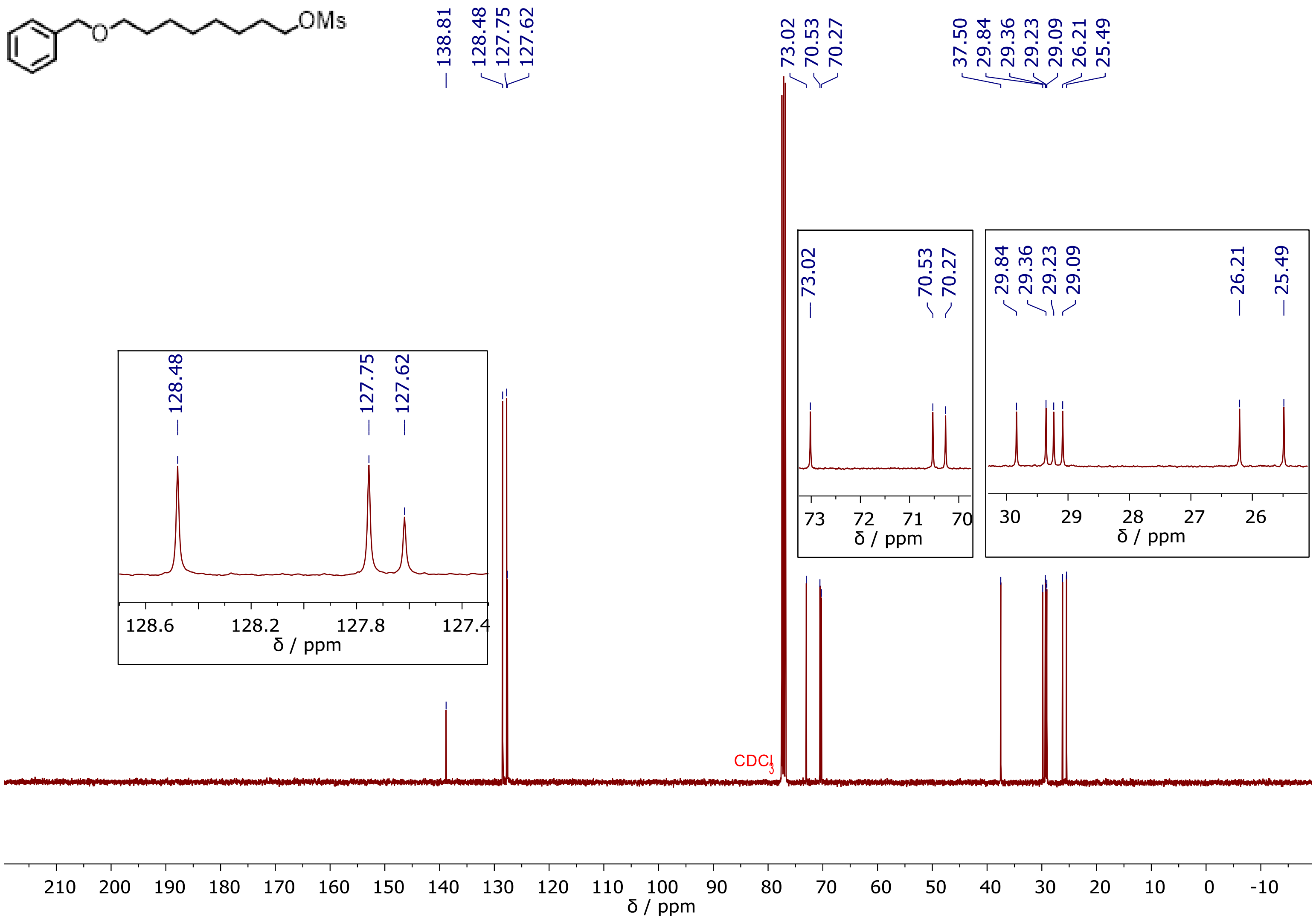

Figure S27: 101 MHz ^{13}C NMR spectrum of **BnOC8OMs** in $CDCl_3$.

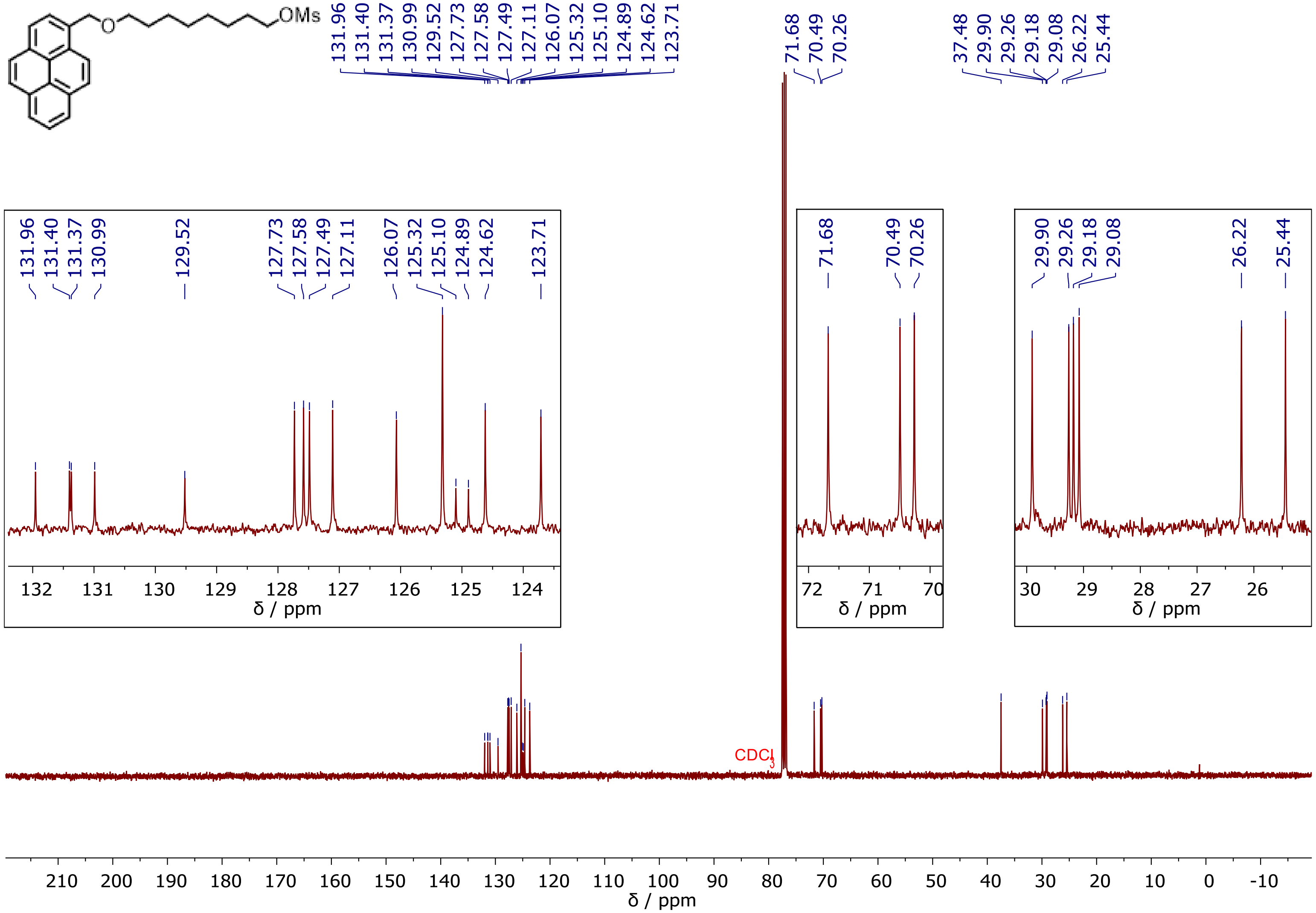

Figure S28: 101 MHz ^{13}C NMR spectrum of **PyrMOC8OMs** in $CDCl_3$.

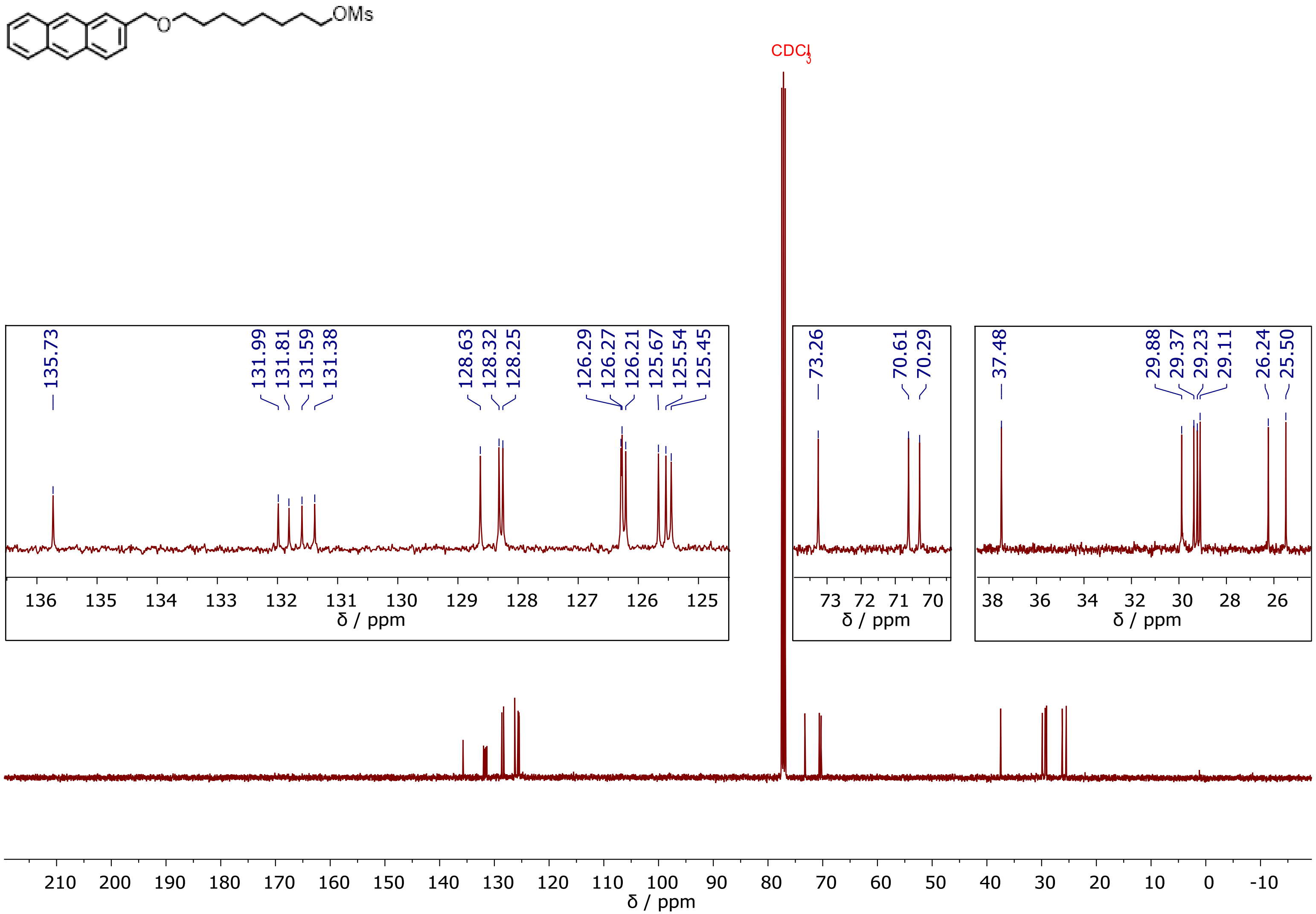

Figure S29: 101 MHz ^{13}C NMR spectrum of **2-AMOC8OMs** in $CDCl_3$.

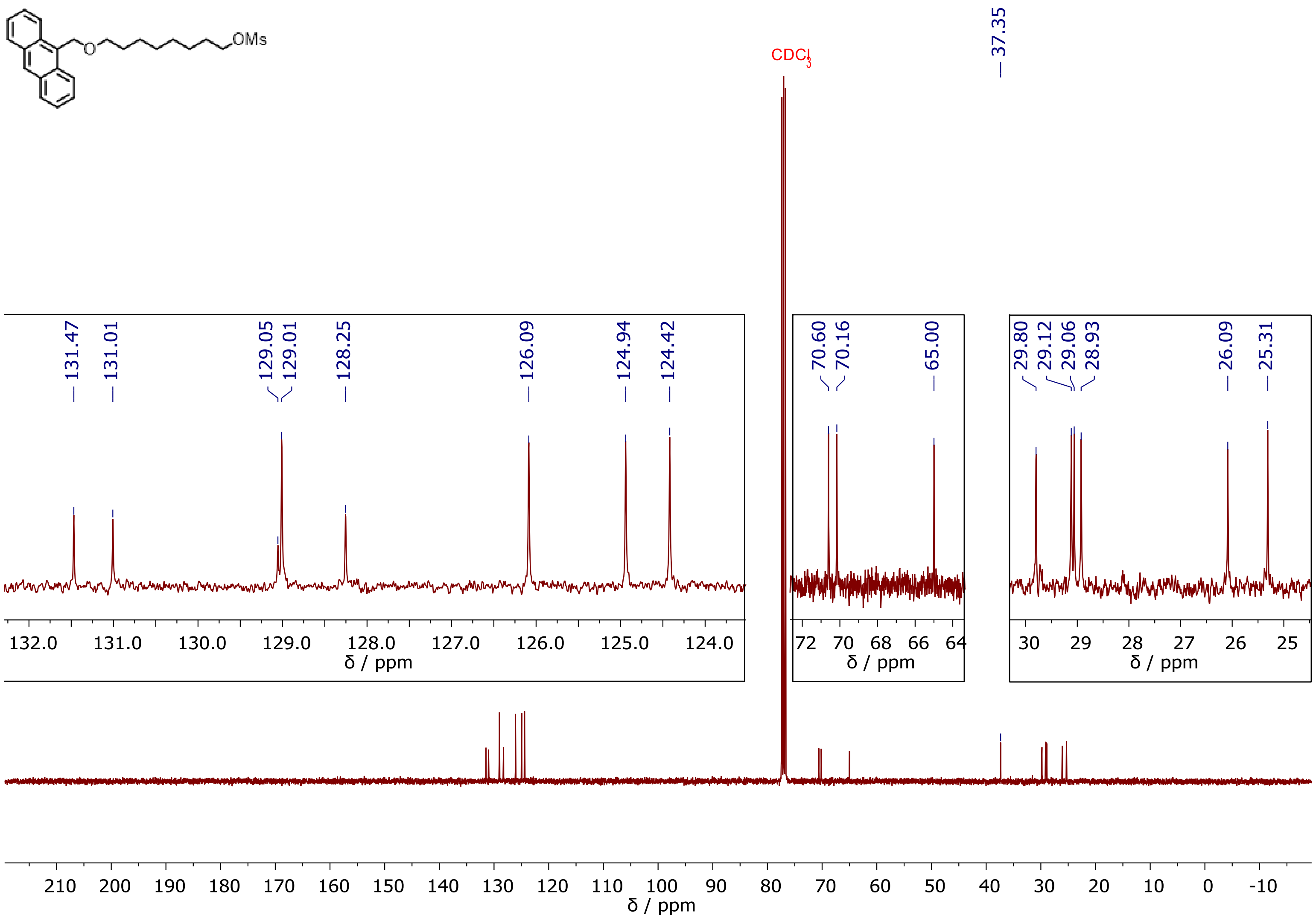

Figure S30: 101 MHz ^{13}C NMR spectrum of **9-AMOC8OMs** in $CDCl_3$.

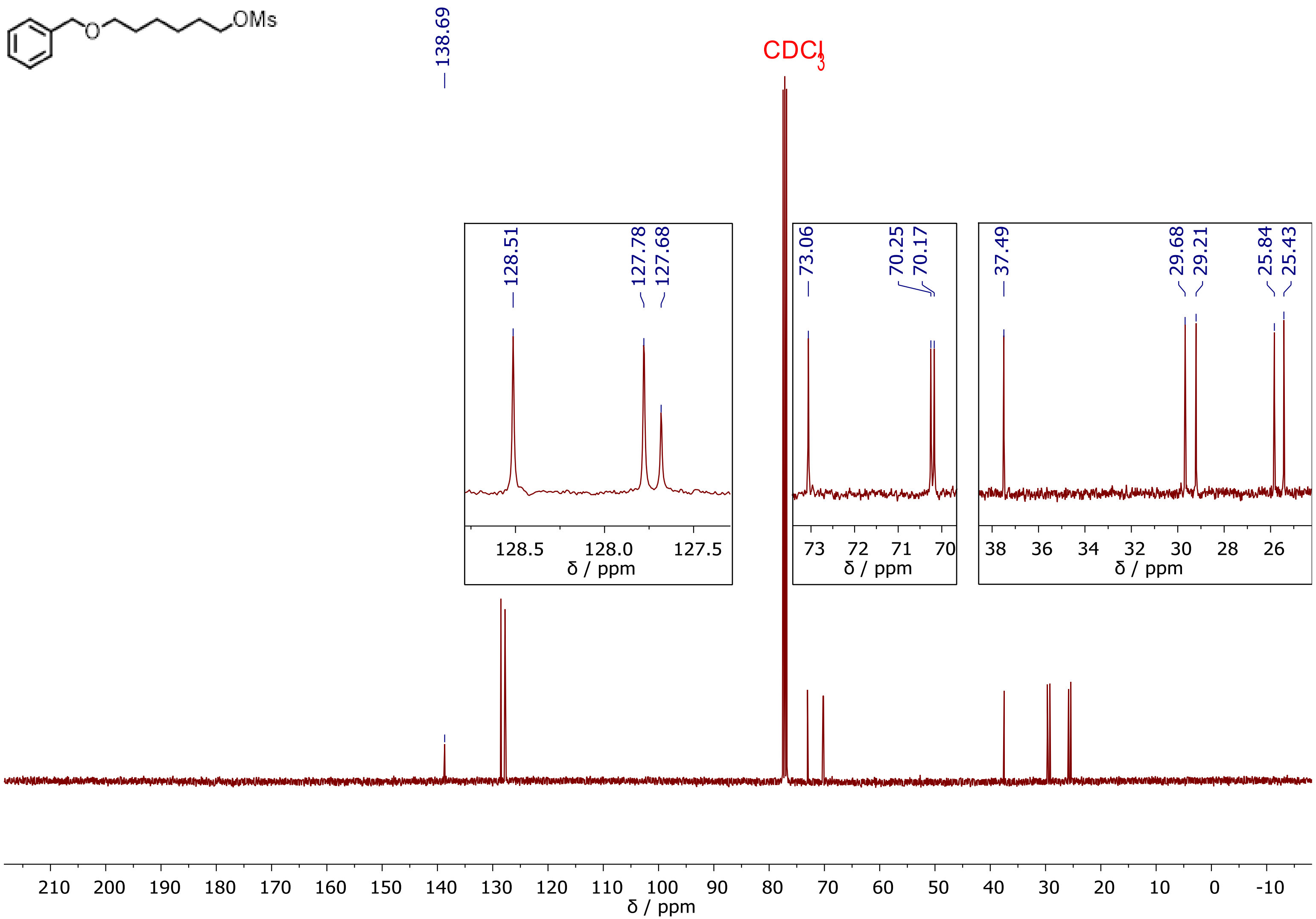

Figure S31: 101 MHz ^{13}C NMR spectrum of **BnOC6OMs** in $CDCl_3$.

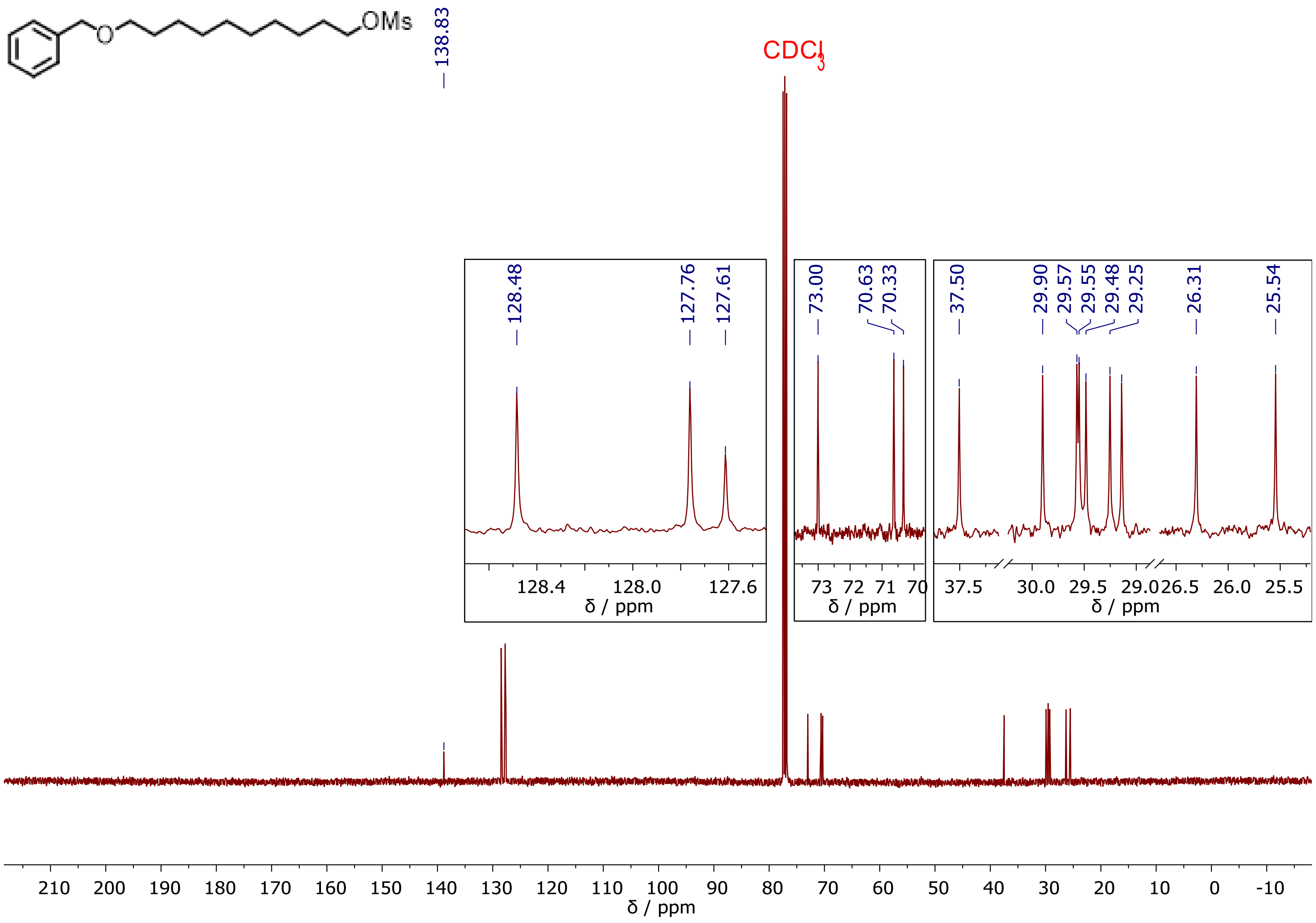

Figure S32: 101 MHz ^{13}C NMR spectrum of **BnOC10OMs** in $CDCl_3$.

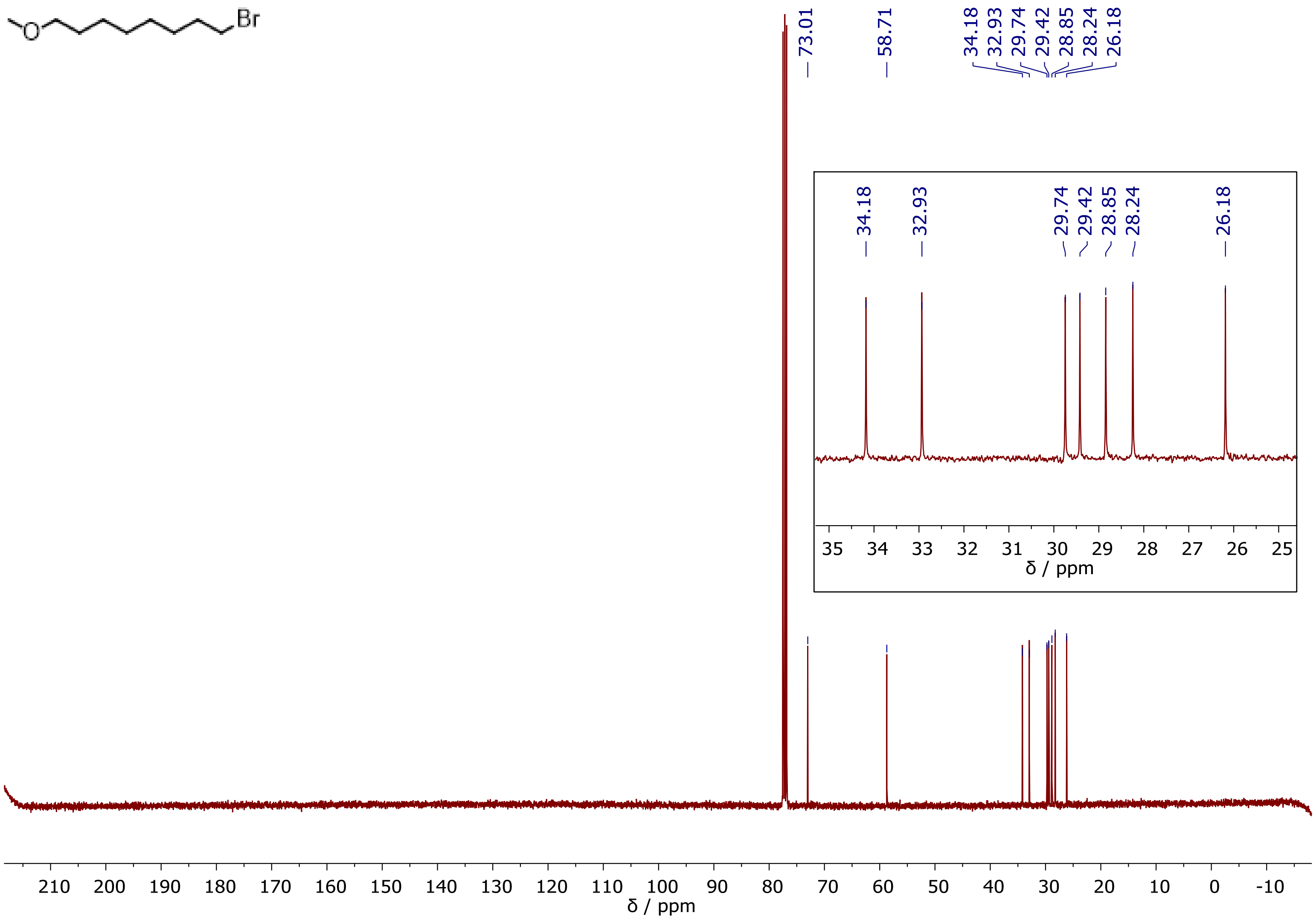

Figure S33: 101 MHz ^{13}C NMR spectrum of **MeOC8Br** in $CDCl_3$.

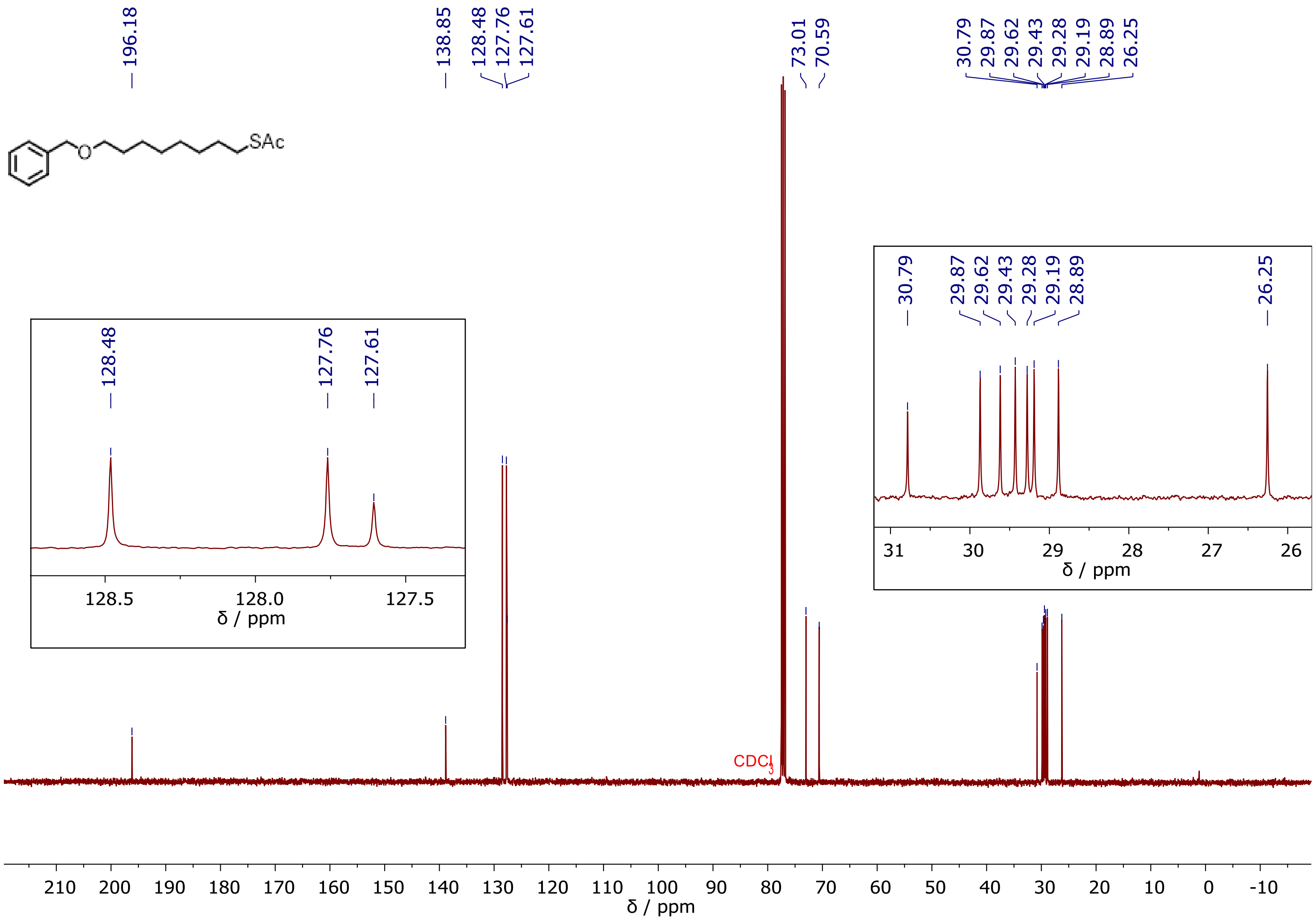

Figure S34: 101 MHz ^{13}C NMR spectrum of **BnOC8SAc** in $CDCl_3$.

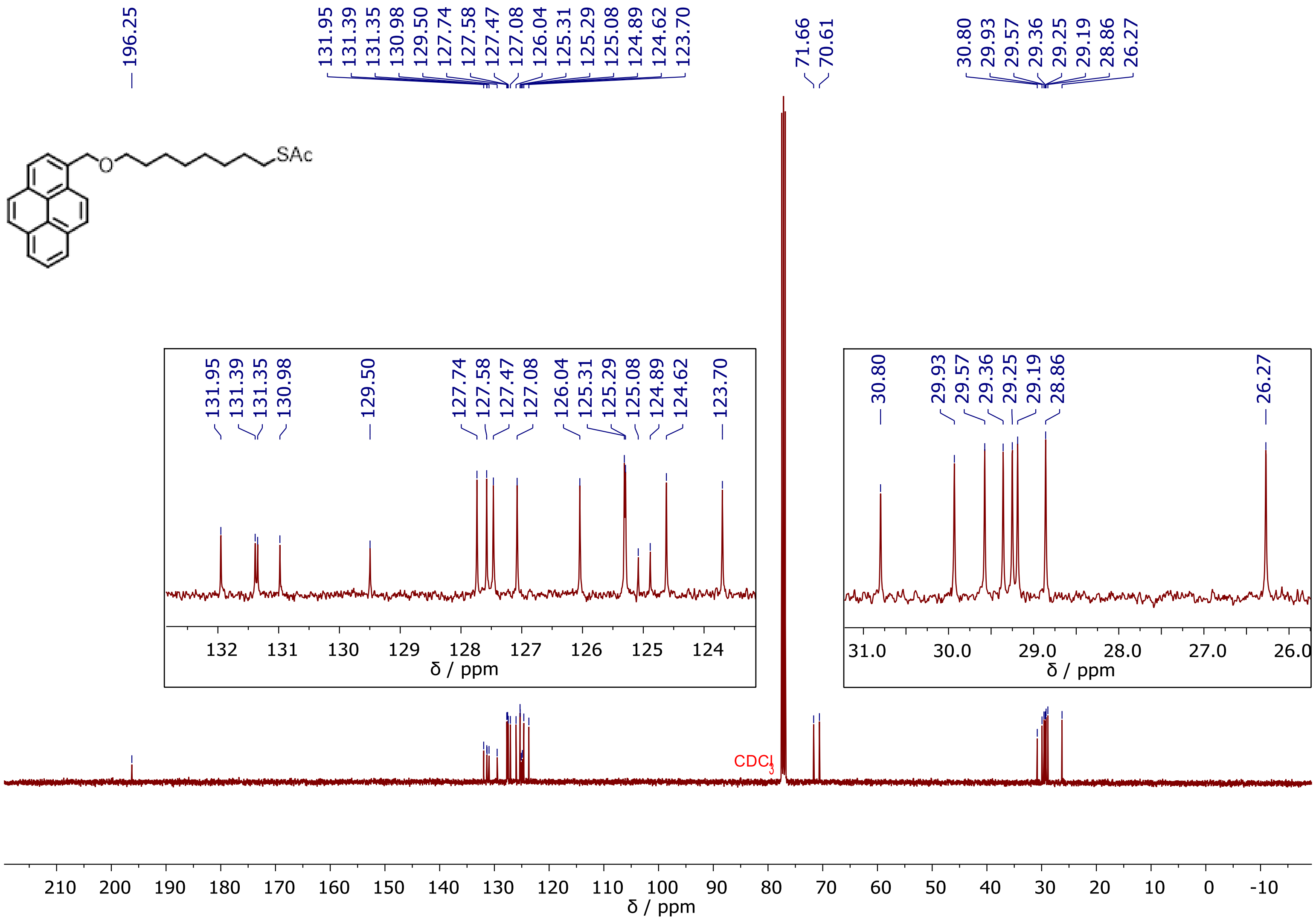

Figure S35: 101 MHz ^{13}C NMR spectrum of **PyrMOC8SAc** in $CDCl_3$.

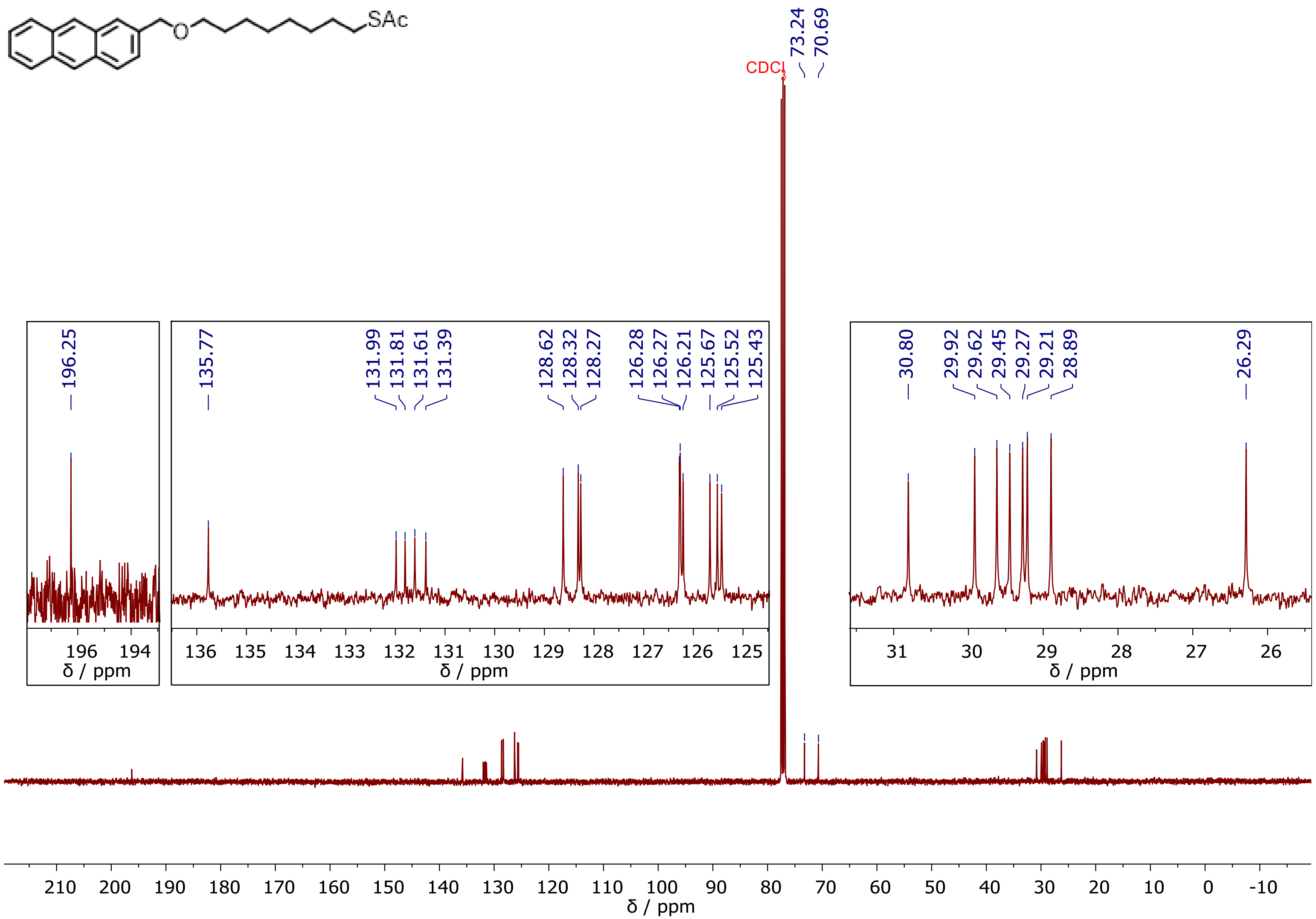

Figure S36: 101 MHz ^{13}C NMR spectrum of **2-AMOC8SAc** in $CDCl_3$.

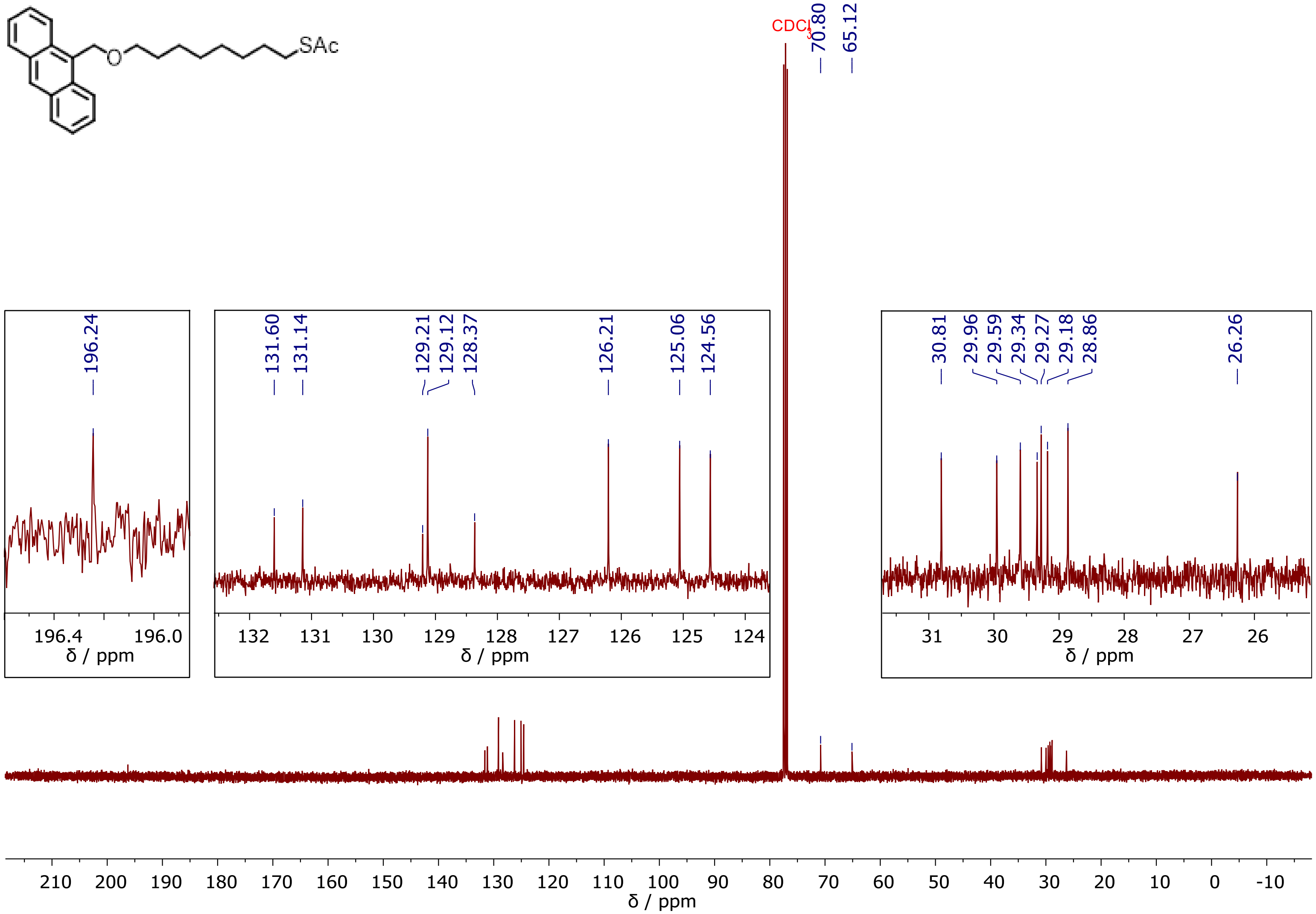

Figure S37: 101 MHz ^{13}C NMR spectrum of **9-AMOC8SAc** in $CDCl_3$.

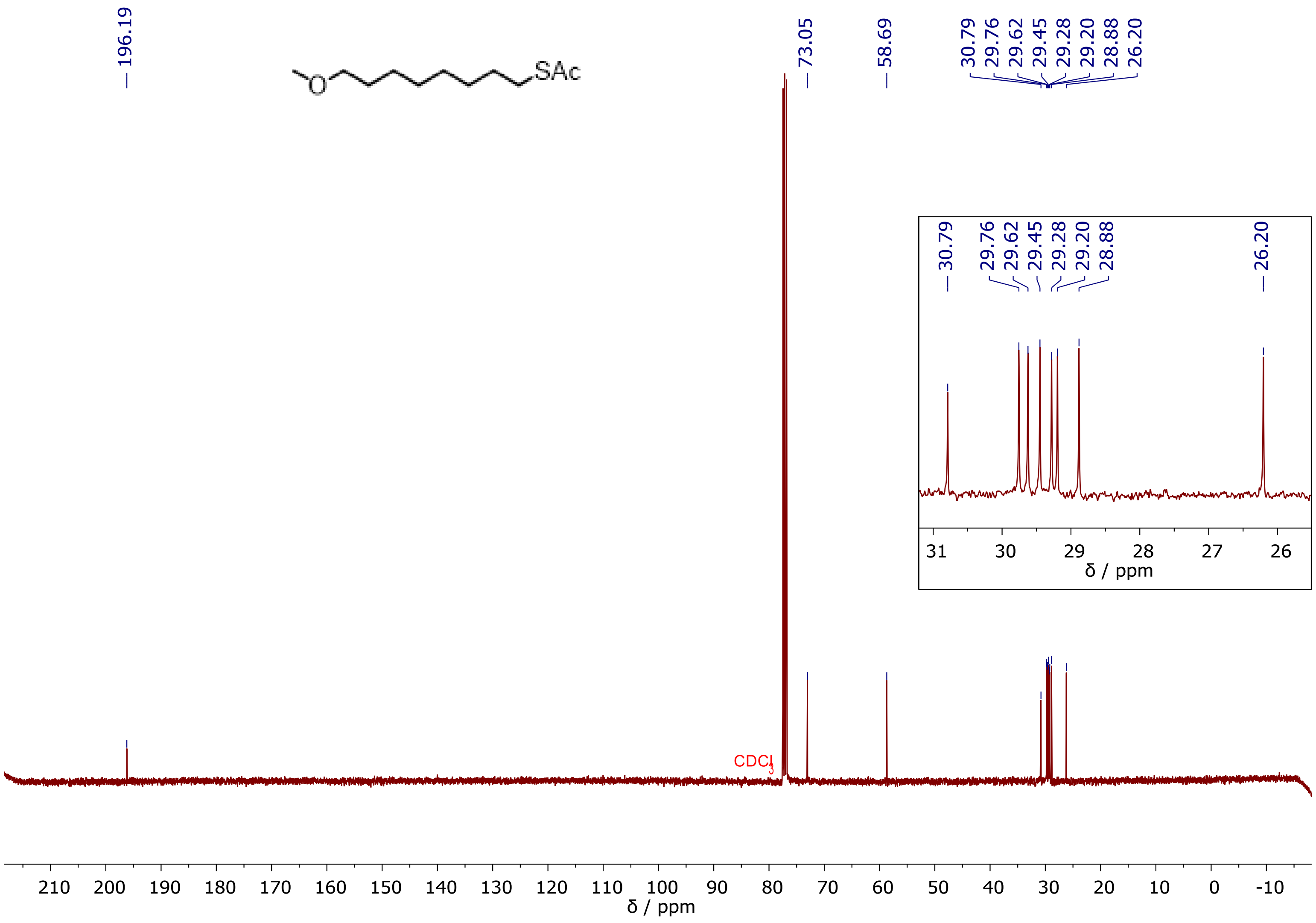

Figure S38: 101 MHz ^{13}C NMR spectrum of **MeOC8SAc** in $CDCl_3$.

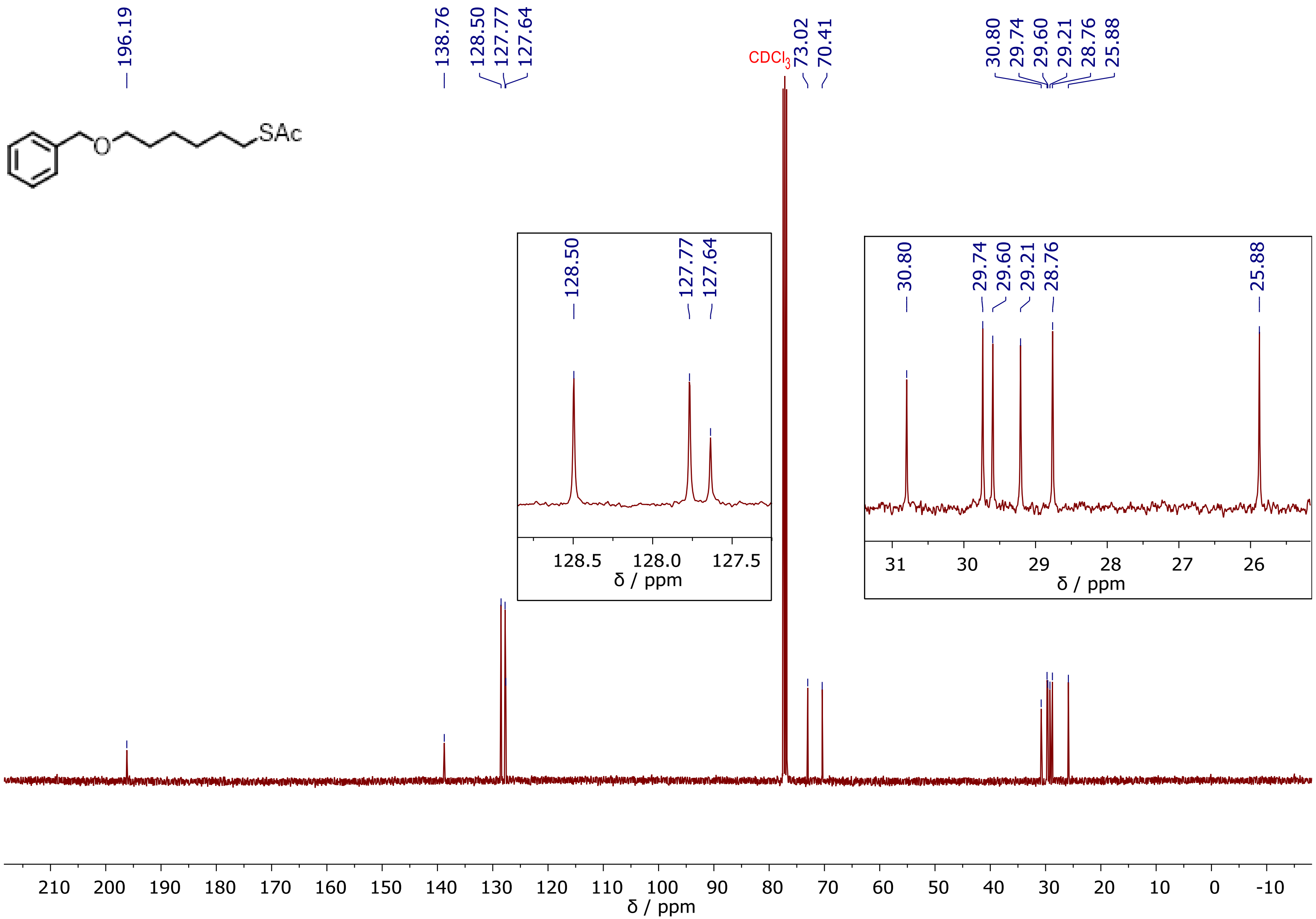

Figure S39: 101 MHz ^{13}C NMR spectrum of **BnOC6SAc** in $CDCl_3$.

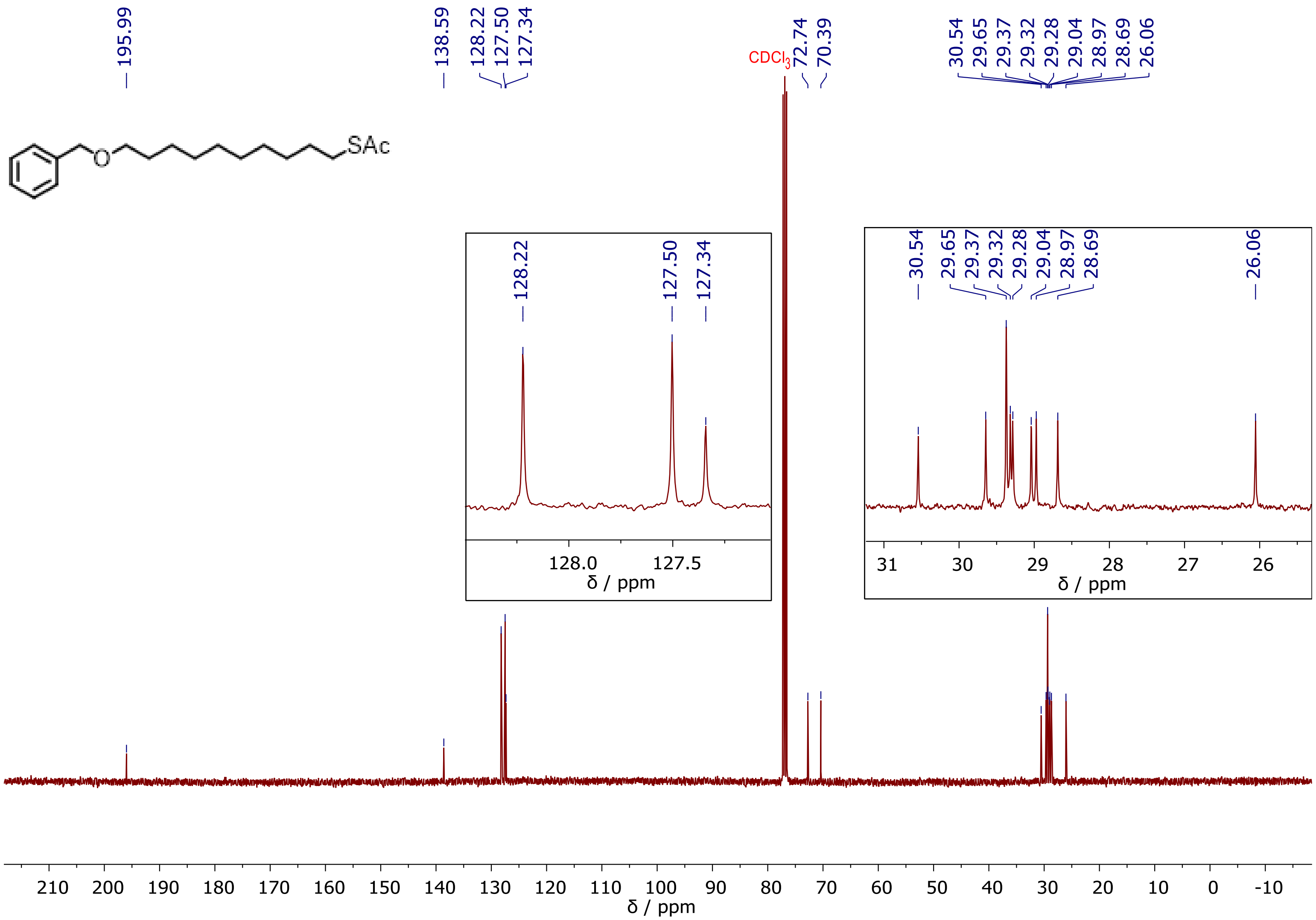

Figure S40: 101 MHz ^{13}C NMR spectrum of **BnOC10SAc** in $CDCl_3$.

S2. Materials Characterisation and Properties

S2.1 SAM Preparation and Characterisation

S2.1.1 – Au^{TS} preparation

Ultra-flat template stripped (TS) gold was prepared on silicon by modifying the methods reported by Whitesides and Pinkhassik.[9, 10] A SiO_2 wafer (1 cm × 1 cm) was washed in an ultrasonic bath sequentially with acetone, methanol and isopropanol and then cleaned with oxygen plasma for 5 minutes. A layer of Au (100 nm) was deposited onto the cleaned wafer by thermal evaporation. A Si wafer was cleaved into small pieces (5 mm x 5 mm) which were cleaned in the same manner. To prepare Au^{TS}, a Si wafer was glued on to the gold surface using EPO-TEK 353ND epoxy adhesive to form a Si/glue/Au/SiO_2 sandwich structure. The glue was cured at 150 °C for 40 min before the ultra-flat TS gold was obtained by cleaving off the SiO_2 with a knife. Each sample of Au^{TS} prepared in this way was imaged using AFM in 3-5 random locations as a quality test, and only those with average roughness lower than 0.2 nm were used for SAM growth.

S2.1.2 – SAM growth

1 mM solutions of the molecules of interest were prepared in ethanol (in the case of **BnOC6SAc**, **BnOC8SAc**, **BnOC10SAc** and **MeOC8SAc**) of DMF (in the case of **PyrMOC8SAc**, **9-AMOC8SAc** and **2-AMOC8SAc)** then purged with nitrogen for 10 minutes to eliminate oxygen. The freshly cleaved Au^{TS} was immersed into the solution without any further treatment and incubated for 24 hours under nitrogen atmosphere. After SAM growth, the sample was rinsed with ethanol and isopropanol several times to remove any physiosorbed molecules, then dried under a stream of nitrogen, and incubated in a vacuum oven (35 °C, 10^{-2} mbar) overnight to remove any residual solvent.

S2.1.3 – Quartz Crystal Microbalance measurement

Quartz crystal microbalance (QCM) measurements used an openQCM Q-1 (Novaetech Srl, Italy) system. A new QCM crystal (5 mm diameter, f0 = 5 MHz, icryst) was cleaned with oxygen plasma for 10 minutes, immersed in hot DMF (100 °C) for 2 hours, transferred into room temperature DMF overnight, then washed with ethanol and isopropanol before drying in a vacuum oven for 20 hours at 35 °C. The initial resonance frequency of the cleaned substrate was recorded, and the substrate was used for SAM growth, using the same procedure as for growth on Au^{TS}. The frequency of the QCM crystal after SAM growth was then recorded. The difference between the frequency before and after SAM growth, Δf, was used to determine the number of molecules, N_{molecule}, adsorbed on the substrate via the Sauerbrey equation:

$$N_{\text{molecule}} = \frac{k \times \Delta f}{M_w \times A} \times N_A$$

$$k = -\frac{\sqrt{\mu * \rho}}{2 * f_0^2}$$

Where A is the crystal area, Δf is the frequency change, M_w is the molecular weight, N_A is Avogadro's number, μ is the shear modulus of quartz, ρ is the density of quartz and f_0 is the initial frequency. The results are reported in Table S1.

S2.1.4 – Analysis of SAM quality

SAM samples on Au^{TS} were imaged using AFM (Multi-Mode 8, Brucker) in peak force mode using a Budget Sensors multi-75 probe (radius ~10 nm at apex part). The roughness of the sample surface was determined using nano-scope 9.0 software. The thickness of the SAMs was determined by nano-scratching. A small region of the molecular thin film was scratched by a stiff AFM probe at high force (50 nN), then peak force mode was used to scan a larger region (1-2 μm × 1-2 μm) around the scratched area. The scratched window can be observed in large scale scans as exemplified in Figure S41. The height difference between the scratched window and surrounding SAM indicates thickness of the film. Repeated measurements allowed an average film thickness to be determined (Figure S42). SAM characterisation is summarised in Table S1.

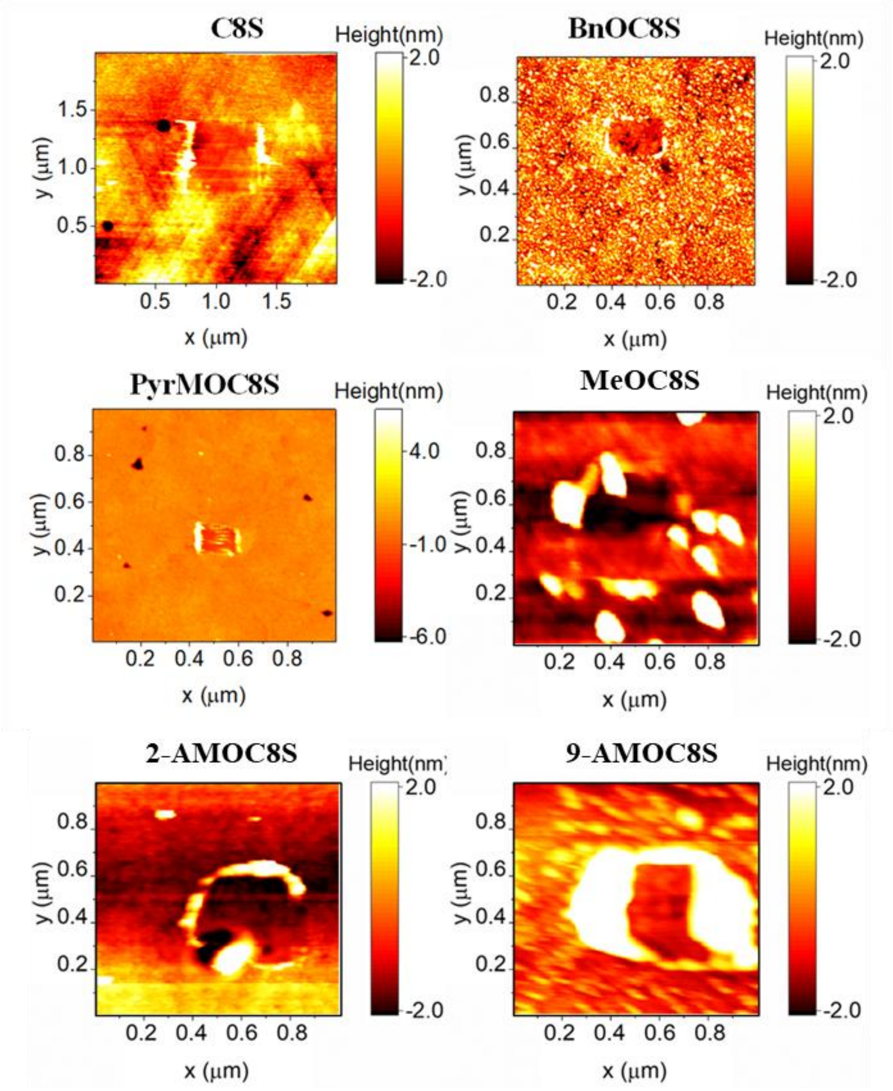

Figure S41: AFM images of SAMs of molecules **C8S**, **BnOC8S**, **PyrMOC8S, MeOC8S, 2-AMOC8S** and **9-AMOC8S** after nano-scratching.

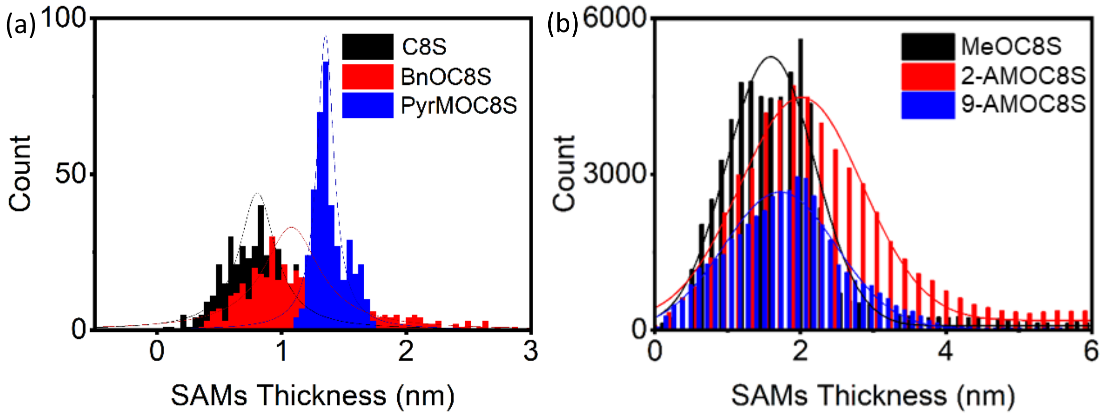

Figure S42: Distributions of SAM thickness for molecules (a) **C8S**, **BnOC8S** and **PyrMOC8S** and (b) **MeOC8S, 2-AMOC8S** and **9-AMOC8S** determined by nano-scratching.

Table S1. Data obtained when characterising SAMs of **C8S**, **BnOC8S** and **PyrMOC8S**.

Molecule	Thickness (nm)	std (nm)	Δf (Hz)	Mw (g/mol)	$N_{molecule}$ (m^{-2})	$A_{molecule}$ ($Å^2$)
**C8SH**	0.6	0.2	19	146	6.90E+18	14.5
**BnOC8S**	1.1	0.5	22	252	4.60E+18	21.7
**PyrMOC8S**	1.4	0.2	36	378	5.10E+18	19.6
**MeOC8SAc**	1.6	0.2	12	218	5.99E+18	16.7
**2-AMOC8SAc**	2.0	0.3	18	395	4.85E+18	20.6
**9-AMOC8SAc**	1.8	0.3	12	395	3.34E+18	22.9

The roughness for Figure S42 (a) was determined by using a 256 x 256 pixel AFM topography image with 1 µm x 1 µm scanning size and for Figure S42 (b) using a 1024 x 1024 pixel image. In both cases, the roughness was obtained from the averaged variation from each pixel in the z direction through the plane. The $N_{molecule}$ values are consistent with a densely packed monolayer.

S2.2 Electrical Characterisation of SAMs

S2.2.1 – cAFM studies

The electrical conductivity of the films was characterised using a conductive AFM setup based on a Multi-mode 8 AFM instrument (Bruker Nano Surfaces). The bottom gold substrate was used as the source, and a Pt/Cr (Multi-75G, Budget Sensors, k = 3 Nm^{-1}) or a graphene coated probe[11] was used as the drain. The force between the probe and the film was controlled by the deflection error set point. A triangular shaped AC bias was added between the source and drain by a voltage generator (Aglient

33500B) and the source to drain current was acquired by a current pre-amplifier (DLPCA200, Femto) providing current-to-voltage conversion. The I-V characteristics were obtained by a Nanoscope 8 controller simultaneously collecting drive bias and current, with subsequent correlation of these values at each time point. Figures S43 and S44 show differential conductance curves for the studied molecules.

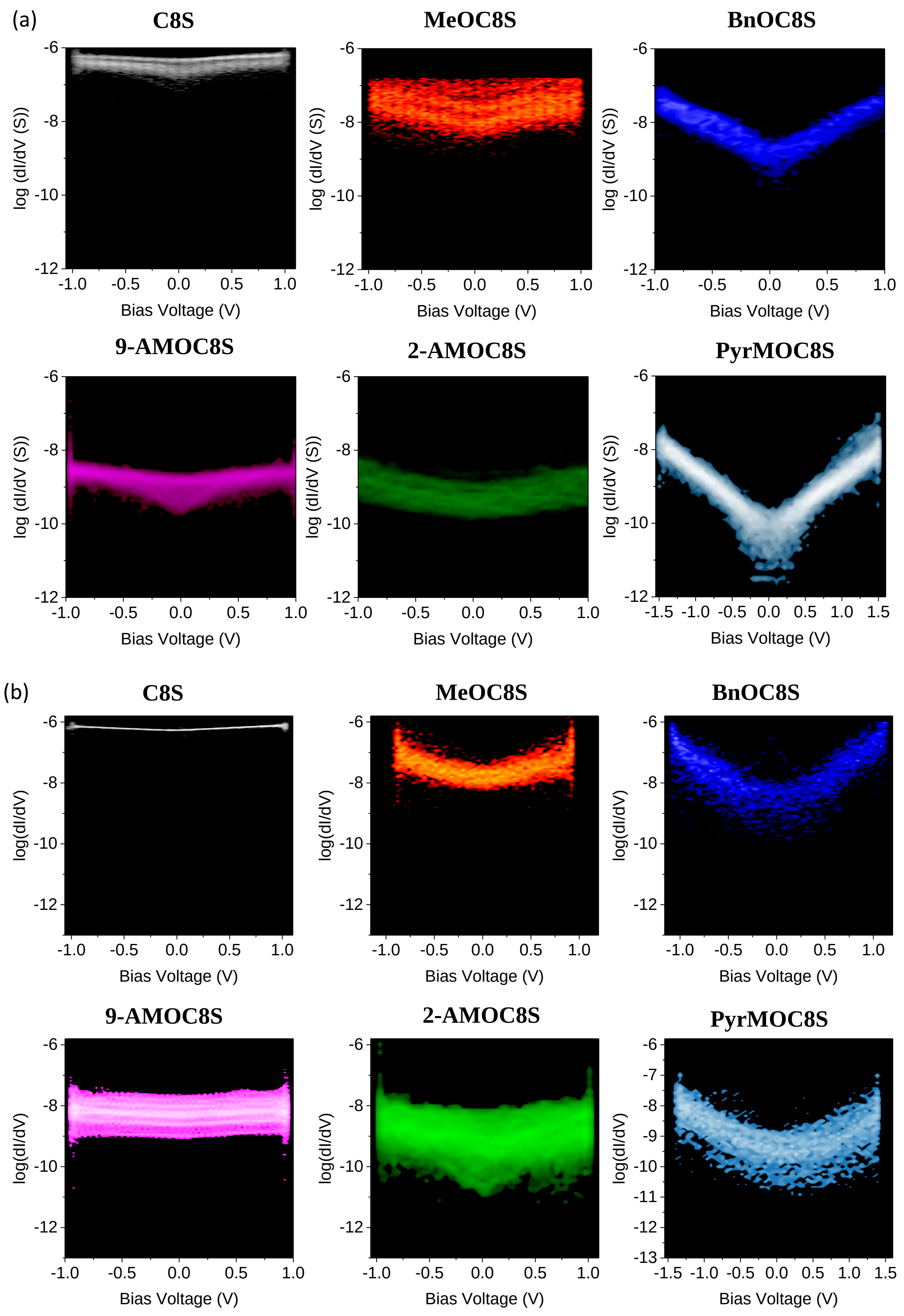

Figure S43: The conductance (dI/dV) of SAMs of [molecule names] at different bias voltage measured using (a) Pt and (b) graphene coated probes

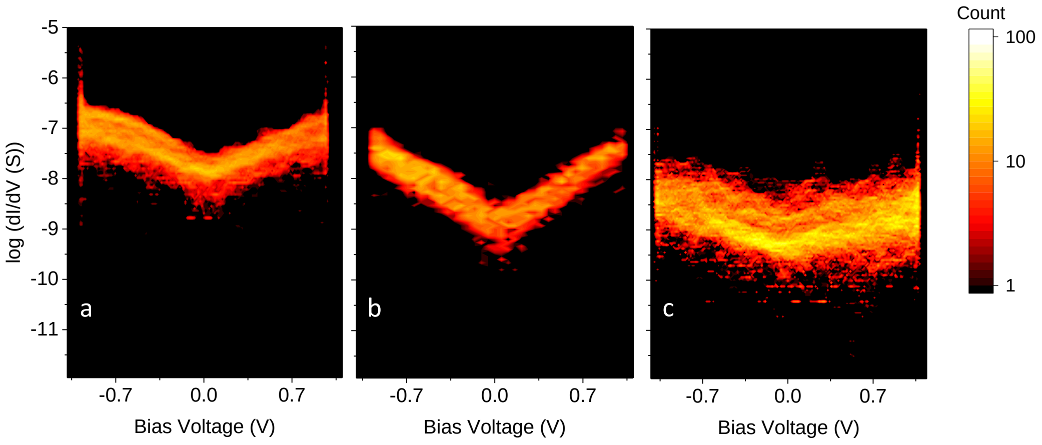

Figure S44: The conductance (dI/dV) of SAMs of (a) **BnOC6S**, (b) **BnOC8S** and (c) **BnOC10S** at different bias voltage.

S2.2.2 – Contact area estimation

The contact area between the probe and the sample was estimated via the JKR model,[12-14] in which the contact radius, r, is calculated from the equations:

$$r = \left(F \times R \times \frac{1}{Y}\right)^{\frac{1}{3}}$$

$$\frac{1}{Y} = \frac{3}{4} \times \left(\frac{1 - v_1^2}{E_1} + \frac{1 - v_2^2}{E_2}\right)$$

where F is the loading force from probe to sample, R is the radius of the probe, v_1 and v_2 are the Poisson ratio of the probe and SAM, respectively, and E_1 and E_2 are the Young's modulus of the probe and SAM, respectively. The radius of the probe was obtained from SEM imaging and estimated to be 25 nm. The Young's modulus was obtained from AFM in peakforce mode. Estimating the contact area using the equations above we calculated the number of molecules in the probe-sample junction based on the single molecule occupation area calculated by QCM (Table S1), this is summarised in Table S2 below.

Table S2. Calculated number of molecules in the probe-sample junction for each molecule investigated.

SAMs	Yong's modulus (MPa):	Molecules in junction
C8S	450	404
MeOC8S	650	340
BnOC8S	3300	151
9AMOC8S	3000	180
2AMOC8S	3000	200
PyrMOC8S	2600	211
BnOC6S	3300	169
BnOC10S	3100	202

S3. Computational Studies

S3.1 Charge Transport Simulations

Electronic structure calculations were conducted using the SIESTA[15] implementation of density functional theory (DFT) with the PBE[16] exchange-correlation functional. For the Au atoms a double zeta basis set was used; for other atoms the basis set was double zeta polarised. The mesh cutoff was 200 Rydberg and structural relaxations were performed to a force tolerance of 0.01 eV/Å.

Molecular junctions were constructed using two leads with a single molecule between two gold electrodes. Each lead consisted of five Au(111) layers periodic in the x and y directions. The whole structure was periodic in the z direction (transport direction). The distance between the molecule and each lead in the z direction was determined by separate relaxations of a single molecule with each lead. The junction was then assembled and the molecule was allowed to further relax, resulting in the S atoms lying in the hollows of the Au surface lattice.

From the Hamiltonian and overlap matrices of the DFT calculation of the junction, Gollum[17] calculates the transmission coefficient $T_{nm}(E)$ between scattering channels n, m in the electrodes, from which the transmission coefficient $T(E) = \sum_{nm} T_{nm}(E)$ is obtained. As discussed in Chapter 17 of Quantum Transport in Nanostructures and Molecules,[18] this is equivalent to the expression

$$T(E) = 4\mathrm{Tr}\left(\Gamma_1 G \Gamma_2 G^{\dagger}\right)$$

where G is the (retarded) Green's function of the junction and Γ_{i} is the imaginary part of the self energy of electrode i . The electrical conductance is obtained from

$$G = G_0 \int_{\infty}^{\infty} \mathrm{T}(E)\left(-\frac{\partial}{\partial E} f(E)\right) dE$$

where E_F is the Fermi energy of the device, $f(E) = \left(e^{(E-E_F)/k_B T} + 1\right)^{-1}$ is the Fermi distribution function and $G_o = \frac{2e^2}{h}$ is the conductance quantum. At low enough temperatures, this is approximated by $G = G_0 T(E_F)$.

The Au|molecule|Au DFT simulations were undertaken to demonstrate that the angle of the head group is a key factor in determining the electrical conductance. It would be computationally expensive to model the distribution of angles revealed by the MD simulations below and furthermore, the distribution of angles in the experiments is also partially dependent on unknown surface roughness. Therefore, the Au|molecule|Au DFT calculations were carried out simply for two extreme angles. The distribution of angles for Au|molecule|Pt junctions is likely to differ from that of Au|molecule|Au junctions, but since neither distribution is known, we believe that a Au|molecule|Au simulation using the two chosen angles is sufficient to illustrate the effect.

The electrode separation is chosen to correspond to the most stable configuration. We first find the ground state geometry and molecule-Au distance when molecules are close to one gold electrode. We then use these distances as the starting point for the geometry optimization of the molecules between two electrodes. Indeed, this does determine the orientation of the head group. If the electrode-molecule distance were increased in the MD simulations below, the molecules would experience more space to move, which could potentially lead to further disorder. As discussed above, this depends on the surface roughness and the distribution of angles, which are unknown and therefore, we take two extreme conditions into account.

S3.2 Molecular Dynamics Simulations

To illustrate how the presence of a top electrode affects the distribution of contact angles with the aromatic head groups of the studied molecules, the dynamics of a SAM of **BnOC8S** molecules were investigated with a molecular dynamics simulation using the DL_POLY classic[19] package, with the OPLS 2005[20, 21] force field. DL_FIELD was used to prepare input files.[22] The sulfur atoms were frozen, mimicking the effect of bonding to a Au surface (in the absence of the Au surface in the simulation, the sulfur atoms were hydrogen-terminated to give neutral species). The simulation consisted of 1 ps of equilibration at 293 K followed by a 100 ps production run in the microcanonical ensemble, with a timestep of 1 fs. A snapshot of the simulation is shown in Figure S45. There were 25 **BnOC8SH** molecules in the simulation cell, placed at intervals of 5.0 Å in a $(\sqrt{3} \times \sqrt{3})R30$ pattern with periodic boundary conditions.[23] The sulfur atoms were placed in the $z = 0$ plane. The distribution of angles θ which the head groups made with that plane are shown in Figure S47, in the presence of a graphene top contact (left) and without any top contact (right).

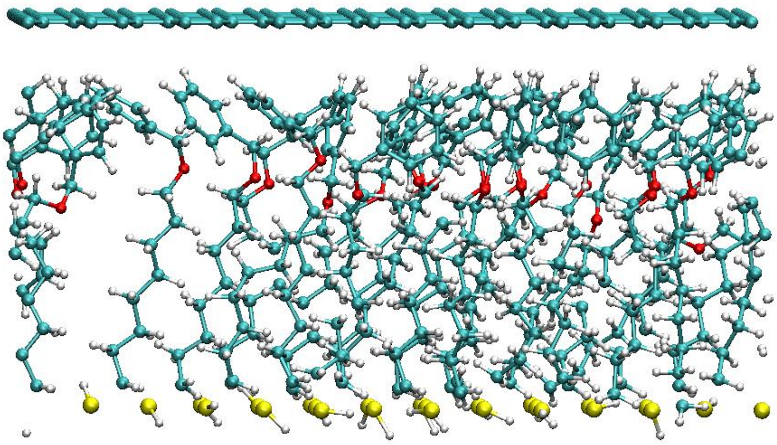

Figure S45: A snapshot of the molecular dynamics simulation for a SAM of **BnOC8SH** molecules under a graphene top contact. Some bonds are not visible.

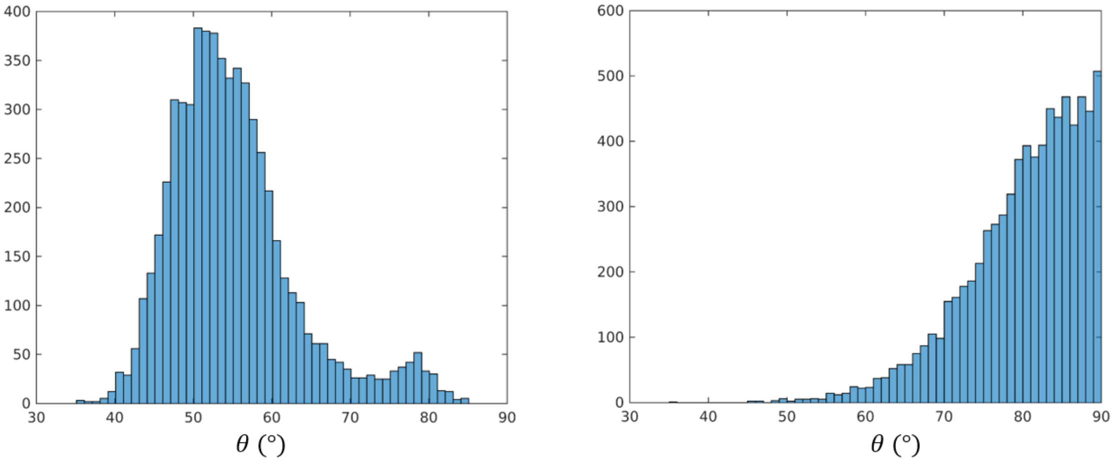

Figure S46: Distribution of head group angles of **BnOC8S** molecules from MD simulation, measured from plane of top graphene contact, with graphene present (left) and without the presence of graphene (right).

Further calculations for other molecules from the studied series showed a similar tendency to large values of θ in the absence of a top contact electrode, as shown in Figure S47.

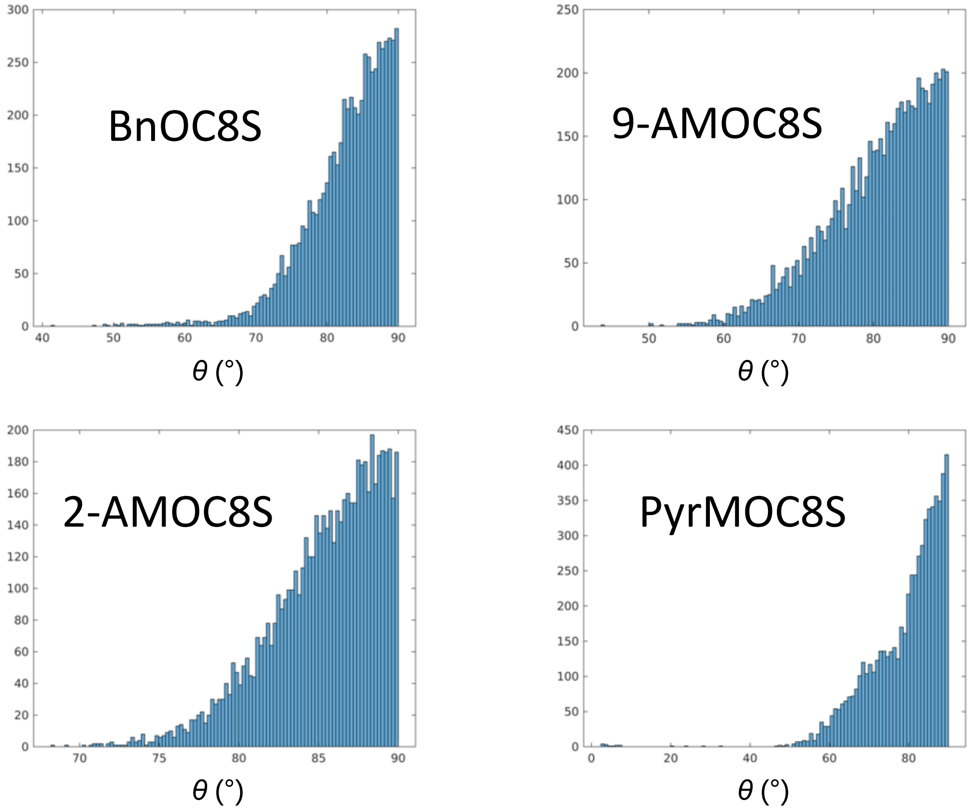

Figure S47: Distribution of head group angles from MD without graphene present.

References

1. H. E. Gottlieb, V. Kotlyar and A. Nudelman, *J. Org. Chem.*, 1997, **62**, 7512-7515.
2. L. J. O'Driscoll, D. J. Welsh, S. W. D. Bailey, D. Visontai, H. Frampton, M. R. Bryce and C. J. Lambert, *Chem. Eur. J.*, 2015, **21**, 3891-3894.
3. S. Ito, Y. Torihata and K. Mikami, *ChemistrySelect*, 2016, **1**, 3310-3315.
4. I. Cade, N. J. Long, A. J. P. White and D. J. Williams, *J. Organomet. Chem.*, 2006, **691**, 1389-1401.
5. B. V. Subba Reddy, B. Anusha, U. V. Subba Reddy, J. S. Yadav and C. Suresh Reddy, *Helv. Chim. Acta*, 2013, **96**, 1983-1990.
6. R. S. Narayan and B. Borhan, *J. Org. Chem.*, 2006, **71**, 1416-1429.
7. O. Zenasni, M. D. Marquez, A. C. Jamison, H. J. Lee, A. Czader and T. R. Lee, *Chem. Mater.*, 2015, **27**, 7433-7446.
8. M. Malkoch, E. Malmström and A. Hult, *Macromolecules*, 2002, **35**, 8307-8314.
9. E. A. Weiss, G. K. Kaufman, J. K. Kriebel, Z. Li, R. Schalek and G. M. Whitesides, *Langmuir*, 2007, **23**, 9686-9694.
10. L. T. Banner, A. Richter and E. Pinkhassik, *Surf. Interface Anal.*, 2009, **41**, 49-55.

11. L. J. O'Driscoll, X. Wang, M. Jay, A. S. Batsanov, H. Sadeghi, C. J. Lambert, B. J. Robinson and M. R. Bryce, *Angew. Chem. Int. Ed.*, 2020, **59**, 882-889.
12. N. A. Burnham, R. J. Colton and H. M. Pollock, *Phys. Rev. Lett.*, 1992, **69**, 144-147.
13. K. L. Johnson, K. Kendall, A. D. Roberts and D. Tabor, *Proc. R. Soc. London, Ser. A*, 1971, **324**, 301-313.
14. H. Song, H. Lee and T. Lee, *J. Am. Chem. Soc.*, 2007, **129**, 3806-3807.
15. J. M. Soler, E. Artacho, J. D. Gale, A. García, J. Junquera, P. Ordejón and D. Sánchez-Portal, *J. Phys.: Condens. Matter*, 2002, **14**, 2745.
16. J. P. Perdew, K. Burke and M. Ernzerhof, *Phys. Rev. Lett.*, 1996, **77**, 3865-3868.
17. J. Ferrer, C. J. Lambert, V. M. García-Suárez, D. Z. Manrique, D. Visontai, L. Oroszlany, R. Rodríguez-Ferradás, I. Grace, S. W. D. Bailey, K. Gillemot, S. Hatef and L. A. Algharagholy, *New J. Phys.*, 2014, **16**, 093029.
18. C. J. Lambert, *Quantum Transport in Nanostructures and Molecules*, IOP Publishing, 2021.
19. W. Smith and T. R. Forester, *J. Mol. Graph.*, 1996, **14**, 136-141.
20. J. L. Banks, H. S. Beard, Y. Cao, A. E. Cho, W. Damm, R. Farid, A. K. Felts, T. A. Halgren, D. T. Mainz, J. R. Maple, R. Murphy, D. M. Philipp, M. P. Repasky, L. Y. Zhang, B. J. Berne, R. A. Friesner, E. Gallicchio and R. M. Levy, *J. Comput. Chem.*, 2005, **26**, 1752-1780.
21. W. L. Jorgensen, D. S. Maxwell and J. Tirado-Rives, *J. Am. Chem. Soc.*, 1996, **118**, 11225-11236.
22. C. W. Yong, *J. Chem. Inf. Model.*, 2016, **56**, 1405-1409.
23. E. Pensa, C. Vericat, D. Grumelli, R. C. Salvarezza, S. H. Park, G. S. Longo, I. Szleifer and L. P. Méndez De Leo, *Phys. Chem. Chem. Phys.*, 2012, **14**, 12355-12367.
